# Supplementary material for: The haplotype-resolved chromosome pairs of a heterozygous diploid African cassava cultivar reveal novel pan-genome and allele-specific transcriptome features
Source: Gigascience. 2022 Mar 24;11:giac028. doi: 10.1093/gigascience/giac028 (PMC8952263; doi:10.1093/gigascience/giac028)
Supplement: giac028_GIGA-D-21-00333_Revision_2 [file giac028_giga-d-21-00333_revision_2.pdf]

GigaScience

The haplotype-resolved chromosome pairs of a heterozygous diploid African cassava cultivar reveal novel pan-genome and allele-specific transcriptome features

--Manuscript Draft--

**Manuscript Number:**	GIGA-D-21-00333R2
**Full Title:**	The haplotype-resolved chromosome pairs of a heterozygous diploid African cassava cultivar reveal novel pan-genome and allele-specific transcriptome features
**Article Type:**	Research
**Funding Information:**	Bill and Melinda Gates Foundation (INV-008213) — Proffessor Wilhelm Gruissem
**Abstract:**	Background<br>Cassava (Manihot esculenta) is an important clonally propagated food crop in tropical and sub-tropical regions worldwide. Genetic gain by molecular breeding has been limited, partially because cassava is a highly heterozygous crop with a repetitive and difficult to assemble genome.<br>Findings<br>Here we demonstrate that Pacific Biosciences high-fidelity (HiFi) sequencing reads, in combination with the assembler hifiasm, produced genome assemblies at near complete haplotype resolution with higher continuity and accuracy compared to conventional long sequencing reads. We present two chromosome scale haploid genomes phased with Hi-C technology for the diploid African cassava variety TME204. With consensus accuracy above QV46, contig N50 above 18 Mbp, BUSCO completeness of 99%, and 35 K phased gene loci, it is the most accurate, continuous, complete and haplotype-resolved cassava genome assembly so far. Ab initio gene prediction with RNA-seq data and Iso-Seq transcripts identified abundant novel gene loci, with enriched functionality related to chromatin organization, meristem development and cell responses. During tissue development, differentially expressed transcripts of different haplotype origins were enriched for different functionality. In each tissue, 20-30% of transcripts showed allele-specific expression (ASE) differences. ASE bias was often tissue-specific and inconsistent across different tissues. Direction-shifting was observed in less than 2% of the ASE transcripts. Despite high gene synteny, the HiFi genome assembly revealed extensive chromosome re-arrangements and abundant intra-genomic and inter-genomic divergent sequences, with large structural variations mostly related to LTR-retrotransposons. We use the reference-quality assemblies to build a cassava pan-genome and demonstrate its importance in representing the genetic diversity of cassava for downstream reference-guided omics analysis and breeding.<br>Conclusions<br>The phased and annotated chromosome pairs allow a systematic view of the heterozygous diploid genome organization in cassava with improved accuracy, completeness and haplotype resolution. They will be a valuable resource for cassava breeding and research. Our study may also provide insights into developing cost-effective and efficient strategies for resolving complex genomes with high resolution, accuracy and continuity.
**Corresponding Author:**	Weihong Qi<br>Functional Genomics Center Zürich: Functional Genomics Center Zurich<br>Zurich, SWITZERLAND
**Corresponding Author Secondary Information:**	
**Corresponding Author's Institution:**	Functional Genomics Center Zürich: Functional Genomics Center Zurich
**Corresponding Author's Secondary Institution:**	
**First Author:**	Weihong Qi
**First Author Secondary Information:**	
**Order of Authors:**	Weihong Qi

Powered by Editorial Manager® and ProduXion Manager® from Aries Systems Corporation

	Yi-Wen Lim
	Andrea Patrignani
	Pascal Schläpfer
	Anna Bratus-Neuenschwander
	Simon Grüter
	Christelle Chanez
	Nathalie Rodde
	Elisa Prat
	Sonia Vautrin
	Margaux-Alison Fustier
	Diogo Pratas
	Ralph Schlapbach
	Wilhelm Gruissem
**Order of Authors Secondary Information:**	
**Response to Reviewers:**	Answers to reviewer reports:<br><br>Reviewer #1: All my concerns have been solved. Thus, I recommend to accept this manuscript.<br><br>Answer: Thank you for the positive feedback. The reviewer’s comments were helpful for improving the manuscript.<br><br>However, before final publication, I suggest the authors to carefully check the typos throughout the whole manuscript. Here I just show you a few examples:<br><br>Answer: Thank you for finding small mistakes. Spelling and grammar in all documents have been checked in Microsoft Words again and by a native English speaker.<br><br>1) Line 128 and 131, "a fold change (FC) > |4|" is not right. Should be log2|FC| >2? Please check.<br>Similar errors were found in Lines 153-155.<br><br>Answer: The reviewer is correct. All have been corrected.<br><br>2) Line 217-218, 'p-vale' should be 'p-value'<br><br>Answer: Corrected.<br><br>3) Fig.6a, in the right sub-figure, DE might mean "differentially expressed", what does EXP mean? expression?<br><br>Answer: Yes. “DE” represents “differentially expressed”. “EXP” represents “expressed”. The explanations have been added into the figure legends of Fig 6a and Fig 6d.<br><br>4) Fig.6c, which color (yellow or purple) represents high and low expression level, respectively?<br><br>Answer: The color scale of the heatmap has been added into Fig 6c.<br><br>Reviewer #2: The revised manuscript is greatly improved; the authors addressed all of my previous concerns.<br><br>Answer: Thank you for the positive feedback. The reviewer’s comments were helpful

	for improving the manuscript.<br><br>Abstract Line 3-4: The lack of genetic gain in cassava is due to many factors, not solely the lack of the ability to generate a reference genome due to its heterozygosity. Rephrase.<br><br>Answer: The sentence “Genetic gain by molecular breeding is limited because cassava has a highly heterozygous, repetitive and difficult to assemble genome.” has been rephrased to the following: “Genetic gain by molecular breeding has been limited, partially because cassava is a highly heterozygous crop with a repetitive and difficult to assemble genome.”<br><br>I think it would be better to use "variable" or "diverged" to refer to ASE rather than inconsistent which implies an experimental problem.<br><br>Answer: The terminology of “consistent ASE bias” and “inconsistent ASE bias” have been established and used in multiple publications (Muzzey et al, Crowley et al, Shao et al; see references below). As defined by Shao et al., “genes showing ASE (ASEGs) in various ways, which can be classified into two patterns: consistent ASEGs such that the ASE was biased toward one parental allele in all tissues/conditions, and inconsistent ASEGs such that ASE was found in some but not all tissues/conditions, including direction-shifting ASEGs in which the ASE was biased toward one parental allele in some tissues/conditions while toward the other parental allele in other tissues/conditions.” We therefore follow the established terminology of previous publications and use “inconsistent” to described ASE biases in our manuscript.<br><br>Muzzey D, et al. Extensive and coordinated control of allele-specific expression by both transcription and translation in Candida albicans. Genome Res. 2014 Jun;24(6):963-73. doi: 10.1101/gr.166322.113.<br><br>Crowley JJ, et al. Analyses of allele-specific gene expression in highly divergent mouse crosses identifies pervasive allelic imbalance [published correction appears in Nat Genet. 2015 Jun;47(6):690]. Nat Genet. 2015;47(4):353-360. doi:10.1038/ng.3222.<br><br>Shao L, et al. Patterns of genome-wide allele-specific expression in hybrid rice and the implications on the genetic basis of heterosis. Proc Natl Acad Sci U S A. 2019 Mar 19;116(12):5653-5658. doi: 10.1073/pnas.1820513116.<br><br>Line 80: Explain how being clonally propagated can increase the number of effective alleles. What is an effective allele? F1 hybrids are heterozygous so how is clonal propagation unique?<br><br>Answer: In the manuscript we wrote that “Clonally propagation can increase the effective number of alleles and heterozygosity in the population.” We explain this statement below supported by relevant literature references. We are not further elaborating the statement in our current manuscript because this will be further explained and substantiated by results in another manuscript that is currently in preparation.<br><br>“Clonal propagation leads to accumulation of somatic mutations [1,2]. As mutations accumulate over time, the growing plant can become a genetic mosaic [3], with cell lineages differing in somatic mutations. A cell lineage with a mutation conferring an advantage in terms of cell proliferation can replace other lineages. Such diplontic selection at the cellular level can purge deleterious mutations, allowing favorable new alleles to accumulate in the plant [4,5]. Gametes can bear the somatic mutations of the cell lineage from which they are derived. When different somatic mutations from different cell lineages are inherited, the clonal descendants of the plant can be genetically heterogeneous. Because continued diplontic selection leads to accumulation of favorable mutations, the gametes are not a random set but are also biased towards favorable mutations. As a source of genetic variation, it has been shown that inherited somatic mutations could be quantitatively comparable to mutations occurring during meiosis [6].”

	1. Whitham TG, Slobodchikoff CN. Evolution by individuals, plant-herbivore interactions, and mosaics of genetic variability: The adaptive significance of somatic mutations in plants. Oecologia. 1981; doi: 10.1007/BF00347587.<br>2. McKey D, Elias M, Pujol B, Duputié A. The evolutionary ecology of clonally propagated domesticated plants. New Phytol. 2010; doi: 10.1111/j.1469-8137.2010.03210.x.<br>3. Gill DE, Chao L, Perkins SL, Wolf JB. Genetic Mosaicism in Plants and Clonal Animals. Annu Rev Ecol Syst. 1995; doi: 10.1146/annurev.es.26.110195.002231.<br>4. Otto SP, Hastings IM. Mutation and selection within the individual. Genetica. 102–103:507–241998;<br>5. Pineda-Krch M, Lehtila K. Cell lineage dynamics in stratified shoot apical meristems. J Theor Biol. 2002; doi: 10.1006/jtbi.2002.3139.<br>6. Orive ME. Somatic mutations in organisms with complex life histories. Theor Popul Biol. 2001; doi: 10.1006/tpbi.2001.1515.<br><br>Clarify which version of hifiasm was used in Supp table 1 vs Table 2. The metrics for the alternate assembly in Supp Table 1 seem poor.<br><br>Answer: The version numbers have been added in Supp table 1 and the header of Table 2. The alternate assembly in Supp Table 1 was without Hi-C phasing. The alternate assembly in Table 2 was phased with Hi-C.<br><br>Line 298 and others: It should be non-mitochondrial not none-mitochondrial<br><br>Answer: Corrected.<br><br>If the pseudochromsomes were assigned randomly to H1 and H2, how can you explain differential GO enrichment in the ASE? What would happen to the enrichment if the pseudochromsomes were reshuffled among the haplotypes?<br><br>Answer: We have now explained this in the discussion and added the new Supplementary figure 10: “Between allelic chromosome pairs, the number of ASE transcripts biased towards each haplotype was similar across the genome (Figure 7 d). Finding ASE transcripts will thus still hold true after reshuffling of pseudochromosomes between haplotypes. The number of DETs between allelic chromosome pairs was also similar (Supplementary figure 10 a). Functional enrichment analysis of DETs per chromosome identified chromosomes with uniquely enriched BP terms (Supplementary figure 10 a and b). The existence of such chromosomes suggests that haplotype-specific enriched functions in DETs will hold true no matter how the chromosomes are shuffled between haplotypes, although the specific set of DETs and actual enriched GO terms per haploid genome will change.”
**Additional Information:**	
**Question**	**Response**
Are you submitting this manuscript to a special series or article collection?	No
**Experimental design and statistics**<br><br>Full details of the experimental design and statistical methods used should be given in the Methods section, as detailed in our Minimum Standards Reporting Checklist. Information essential to interpreting the data presented should be made available in the figure legends.<br><br>Have you included all the information	Yes

requested in your manuscript?	
**Resources**<br><br>A description of all resources used, including antibodies, cell lines, animals and software tools, with enough information to allow them to be uniquely identified, should be included in the Methods section. Authors are strongly encouraged to cite Research Resource Identifiers (RRIDs) for antibodies, model organisms and tools, where possible.<br><br>Have you included the information requested as detailed in our Minimum Standards Reporting Checklist?	Yes
**Availability of data and materials**<br><br>All datasets and code on which the conclusions of the paper rely must be either included in your submission or deposited in publicly available repositories (where available and ethically appropriate), referencing such data using a unique identifier in the references and in the "Availability of Data and Materials" section of your manuscript.<br><br>Have you have met the above requirement as detailed in our Minimum Standards Reporting Checklist?	Yes

Manuscript

Click here to access/download;Manuscript;333-editorV1-ETHZ.docx

The haplotype-resolved chromosome pairs of a heterozygous diploid African cassava cultivar reveal novel pan-genome and allele-specific transcriptome features

Weihong Qi[1,2,4*•], Yi-Wen Lim[2,•], Andrea Patrignani[1], Pascal Schläpfer[2], Anna Bratus-Neuenschwander[1], Simon Grüter[1], Christelle Chanez[2], Nathalie Rodde[5], Elisa Prat[5], Sonia Vautrin[5], Margaux-Alison Fustier[5], Diogo Pratas[6,7], Ralph Schlapbach[1], Wilhelm Gruissem[2,3*]

1. Functional Genomics Center Zurich, ETH Zurich and University of Zurich, Winterthurerstrasse 190, 8057, Zurich, Switzerland
2. Department of Biology, Institute of Molecular Plant Biology, ETH Zurich, Universitätstrasse 2, 8092, Zurich, Switzerland
3. Biotechnology Center, National Chung Hsing University, 145 Xingda Road, Taichung, 40227, Taiwan
4. SIB Swiss Institute of Bioinformatics, 1202 Geneva, Switzerland
5. INRAE, CNRGV French Plant Genomic Resource Center, F-31320, Castanet Tolosan, France
6. Department of Electronics, Telecommunications and Informatics and Institute of Electronics and Informatics Engineering of Aveiro, University of Aveiro, Campus Universitário de Santiago, 3810-193 Aveiro, Portugal
7. Department of Virology, University of Helsinki, Haartmaninkatu 3, 00014 Helsinki, Finland

• Equal contributions.

* Corresponding authors: Weihong Qi and Wilhelm Gruissem

weihong.qi@fgcz.ethz.ch

wilhelm_gruissem@ethz.ch

Author ORCIDs and email addresses:

Qi, W. 0000-0001-8581-908X, weihong.qi@fgcz.ethz.ch

Lim, Y.W. 0000-0003-2213-5777, yi-wen.lim@biol.ethz.ch

Patrignani A. 0000-0001-8019-3911, andrea.patrignani@fgcz.ethz.ch

Schlaepfer, P. 0000-0002-0828-8681, pascal.schlaepfer@biol.ethz.ch

Bratus-Neuenschwander A. 0000-0001-9254-9110, anna.bratus@fgcz.ethz.ch

Grüter S. 0000-0003-0124-8625, simon.oliver.grueter@fgcz.ethz.ch

Chanez, C. C. 0000-0002-3432-4375, christelle.chanez@biol.ethz.ch

Rodde N. 0000-0003-3361-4730, nathalie.rodde@inrae.fr

Prat E. 0000-0001-5640-4264, elisa.prat@inrae.fr

Vautrin S. 000-0003-4446-1426, sonia.vautrin@inrae.fr

Fustier M. 0000-0002-1348-6164, margaux.fustier@inrae.fr

Pratas D. 0000-0003-1176-552, diogo.pratas@helsinki.fi

Schlapbach R. 0000-0002-7488-4262, ralph.schlapbach@fgcz.ethz.ch

Gruissem, W. 0000-0002-1872-2998, wilhelm_gruissem@ethz.ch

Abstract

Background

Cassava (*Manihot esculenta*) is an important clonally propagated food crop in tropical and sub-tropical regions worldwide. Genetic gain by molecular breeding has been limited, partially because cassava is a highly heterozygous crop with a repetitive and difficult to assemble genome.

Findings

Here we demonstrate that Pacific Biosciences high-fidelity (HiFi) sequencing reads, in combination with the assembler hifiasm, produced genome assemblies at near complete haplotype resolution with higher continuity and accuracy compared to conventional long sequencing reads. We present two chromosome scale haploid genomes phased with Hi-C technology for the diploid African cassava variety TME204. With consensus accuracy above QV46, contig N50 above 18 Mbp, BUSCO completeness of 99%, and 35 K phased gene loci, it is the most accurate, continuous, complete and haplotype-resolved cassava genome assembly so far. *Ab initio* gene prediction with RNA-seq data and Iso-Seq transcripts identified abundant novel gene loci, with enriched functionality related to chromatin organization, meristem development and cell responses. During tissue development, differentially expressed transcripts of different haplotype origins were enriched for different functionality. In each tissue, 20-30% of transcripts showed allele-specific expression (ASE) differences. ASE bias was often tissue-specific and inconsistent across different tissues. Direction-shifting was observed in less than 2% of the ASE transcripts. Despite high gene synteny, the HiFi genome assembly revealed extensive chromosome re-arrangements and abundant intra-genomic and inter-genomic divergent sequences, with large structural variations mostly related to LTR-retrotransposons. We use the reference-quality assemblies to build a cassava pan-genome and demonstrate its importance in representing the genetic diversity of cassava for downstream reference-guided omics analysis and breeding.

Conclusions

The phased and annotated chromosome pairs allow a systematic view of the heterozygous diploid genome organization in cassava with improved accuracy, completeness and haplotype resolution. They will be a valuable resource for cassava breeding and research. Our study may also provide insights into developing cost-effective and efficient strategies for resolving complex genomes with high resolution, accuracy and continuity.

Keywords

phased chromosome pairs, haplotype heterozygosity, pan-genome, allele-specific expression

Background

High quality reference genomes are fundamental for genomic analyses, which have revolutionized the fields of biology and medicine. Most plant genomes are challenging to assemble with a high level of accuracy, continuity, and completeness because they vary in size, levels of ploidy and heterozygosity [1]. Particularly, many plant species, including cassava, can be clonally propagated, which can increase the effective number of alleles and heterozygosity [2–4]. Meanwhile, plant genomes are highly repetitive and contain abundant ancient and novel transposable elements [1,5]. Intra-genomic heterozygosity and repeat elements are major sources of genome assembly errors [5,6] . The cassava (*Manihot esculenta*, NCBI:txid3983) genome has a haploid genome size around 750 Mbp [7–9], and is one of the most heterozygous [10] and repetitive [8] of currently sequenced plant genomes [11]. Despite continuous sequencing efforts using different technologies over the last decade, unresolved gaps and haplotypes persist in all chromosomes of currently available cassava genomes [7–10,12].

Cassava is an important staple crop that is clonally propagated in tropical and sub-tropical regions worldwide. The starchy storage roots are an important staple food for nearly a billion people and used for industrial purposes. In Africa, cassava is cultivated mainly by smallholder farmers because the crop produces appreciable yields under a wide array of environmental conditions. However,

production is constrained by weeds, drought, pests, and most crucially, viral diseases. Therefore, breeding of more robust and productive cassava varieties is of high importance. Since conventional breeding of cassava is time-consuming, complete haplotype-resolved reference genomes with high accuracy will be a valuable resource for applications of genomic selection, genome editing and improving genetic gains in cassava breeding.

Continuous long reads (CLRs) produced by Pacific Biosciences (PacBio) Single Molecule, Real-Time (SMRT) sequencing technology and other long read sequencing technologies have been essential for generating reference quality genome assemblies cost effectively in the last decade [13]. The African cassava cultivars TME3 and 60444 have been sequenced and assembled using 70-fold PacBio CLRs (read N50 12 Kbp), producing genome assemblies with contig N50 of 98 and 117 Kbp, respectively [8], which were lower than the continuity metric of a high-quality genome proposed by the Vertebrate Genome Project (VGP) consortium (contig N50 > 1 Mbp) [14]. More than 18 K gene loci in TME3 and 60444 were resolved with two haplotype alleles [8], but collapsed regions still persist throughout both assemblies, due to the fact that assembly of error-prone long sequencing reads (hereafter referred to as long reads) homogenized sequences from different haplotype alleles, paralogous loci and repeat elements [15]. The recently introduced PacBio high-fidelity (HiFi) sequencing technology is able to produce long (10-25 Kbp) and highly accurate (>99.9%) sequencing reads (hereafter referred to as HiFi reads). For several human and animal genomes, equivalent or higher continuities have been achieved with HiFi reads [15–18]. Novel genome assemblers have been developed to leverage the full potential of HiFi reads [15,19], where the combined performance of HiFi reads and HiFi-specific genome assemblers was benchmarked in assembling human and animal genomes. Their potential in assembling plant genomes is less well studied, but is gaining momentum [19,20]. In comparison to the strawberry reference genome reconstructed from a combination of short Illumina sequencing reads and PacBio CLR [21], the HiFi assembly of Fragaria x ananass has contig N50 values that are 10 times higher. HiFi reads also enabled the assembly of

the 35.6 Gbp California redwood genome [19]. The recently published haplotype-resolved potato genome [20] was generated using a combination of multiple sequencing strategies, including HiFi reads.

Data description

In this study, we collected PacBio CLRs (ERR5487554 - ERR5487559), HiFi reads (ERR5485301), Illumina paired-end (PE) sequencing reads (hereafter referred to as Illumina PE reads) (ERR5484652), and Hi-C data (ERR5484651) for the African cassava cultivar TME204. It belongs to a group of cassava cultivars carrying the dominant monogenic CMD2 resistance locus, which provides resistance to Cassava Mosaic Diseases (CMD) caused by African Cassava Mosaic Viruses [22]. We benchmarked the performance of CLRs and HiFi reads in assembling this highly complex and heterozygous genome. Assembly continuity, accuracy, and haplotype resolution of different genome drafts produced by four CLR/HiFi assemblers [15,23,24] were evaluated using genome quality metrics proposed by the VGP consortium [25] with Illumina PE reads from the same sample. Our results demonstrate that HiFi reads are valuable in assembling a high-quality heterozygous and repetitive plant genome. The high base accuracy and long sequencing read length provide superior resolution and accuracy in resolving allele differences between haplotypes, paralogous genes and repeat elements. By combining HiFi reads with Hi-C data we produced a highly accurate, chromosome-scale, phased assembly for a diploid African cassava cultivar. The two haploid assemblies (PRJNA758616 and PRJNA758615) revealed extensive haplotype heterozygosity within a cassava diploid genome and provided a systematic view of the cassava diploid genome organization with improved accuracy, completeness and haplotype resolution. To improve genome annotation, we further generated PacBio Iso-Seq reads (ERR5489420 - ERR5489422) from different tissues. In combination with public TME204 RNA-seq data from nine tissues, *ab initio* gene prediction with experimental evidence identified 20 K chromosomal novel gene loci, with enriched functionality related to chromatin organization. The close to complete haplotype resolved, annotated genome

also enabled pan-genome and allele-specific expression analysis, demonstrating the importance of a more complete representation of cassava genetic diversity for downstream reference-guided omics analysis and molecular breeding.

Analyses

Cassava TME204 genome characteristics

Illumina PE reads (Table 1) were used to estimate the overall genome characteristics of TME204, revealing a highly heterozygous diploid genome different from the reference genome of the partially in-bred South-American cassava cultivar AM560 [7] and other well-studied genomes, such as the human reference genome. The peak for k-mers covering TME204 heterozygous sequence was as high as the peak corresponding to k-mers present in both haplotypes, while the k-mer coverage plots for the cassava and human reference genomes were dominated by their homozygous sequence peaks (Supplementary figure 1 a). Based on the number of variant-induced branches in the De Bruijn assembly graph [26], the level of heterozygosity in the TME204 genome was measured at 1%, which is a magnitude higher than the heterozygosity level in the cassava and human reference genomes (Supplementary figure 1 b). This value is a conservative estimate because it is based on genomic regions only with lower rates of nonstructural variations. Highly heterozygous regions introduce divergent paths with higher complexity, which cannot be resolved by conventional bubble calling algorithms used to calculate variant-induced branching rate [27]. Consequently, sequences with a high density of single nucleotide polymorphisms (SNPs), small insertions and deletions (indels $\leq$ 50 bp), and large structural variations (SVs, e.g. indels > 50 bp, duplications, inversions, and translocations) were not counted in the 1% of heterozygosity. The cassava genomes (TME204 and AM560) are more repetitive than the human reference genome (Supplementary figure 1 c), which makes them more difficult to assemble with high quality.

Table 1. Cassava TME204 shotgun sequencing data collected.

	PacBio CLR	PacBio HiFi reads	Illumina PE reads
Sequencer	PacBio Sequel	PacBio Sequel II	Illumina NovaSeq
Chemistry	Sequel binding kit 3.0	Sequel II binding kit 2.0	TruSeq DNA Nano
Number of SMRT cells	6 (1M v3 cells)	1 (8M cell)	NA
Number of reads	5,037,588	1,531,543	259,505,436
Number of bases (bp)	90,586,242,030	31,312,160,541	77,851,630,800
Read length N50 (bp)	29,274	20,363	2 X 150
Estimated coverage [a]	121x	42x	104x
Accession numbers	ERR5487554 - ERR5487559	ERR5485301	ERR5484652

[a] Based on a haploid genome size of 750 Mbp

Benchmarking cassava TME204 assemblies from PacBio CLR and HiFi reads

PacBio HiFi sequencing yielded 42x HiFi reads with length N50 of 20 Kbp (Table 1). To assess the performance of different assemblers, the HiFi reads were assembled using four HiFi-specific software tools: Falcon, HiCanu, hifiasm and IPA (see Methods). For comparison of the HiFi reads with traditional long reads, we also assembled 121x PacBio CLRs (Table 1) from the same DNA sample using the Falcon assembler.

With the same amount of computing resources, HiFi read assembling was about two orders of magnitude faster and required ten times less data storage than CLR assembling. Each HiFi assembly was completed in only a few hours to a few days and used 20-800 GB of data storage when running on a single server with 64 CPUs and 500 GB RAM (random-access memory). CLR-Falcon assembly took a few weeks and used about 7 TB of disk space.

Assembled genome sizes varied based on the assembly software (Figure 1 a, Supplementary table 1 and 2, Supplementary results). HiFi reads improved contig continuity, doubling contig N50 and NG50 values when assembled using Falcon (Figure 1 b). According to the more comparable NG50 values (Supplementary results), the hifiasm contig set was the most continuous (NG50 33 Mbp), followed by the HiCanu contig set (23 Mbp) (Figure 1 b). HiFi reads also improved accuracy and completeness of the assembled genome sequences (Supplementary table 3). When measured using alignments of Illumina PE reads from the same sample (Table 1), both hifiasm and HiCanu achieved superior base accuracy (0.2% error rate) (Figure 1 c), structural accuracy (99.3% mapped reads were correctly paired) (Figure 1 d), and assembly completeness (99.9% mapped reads) (Figure 1 e). When measured using the Merqury k-mers comparisons of Illumina reads and assembled contigs [28], the hifiasm assembly was most accurate (99.997%, quality value (QV) 46.74) (Figure 1 f, Supplementary results) and complete (98.40%) (Figures 1 e, Supplementary results). Because a proper assembly of PacBio CLR reads requires signal level polishing to achieve satisfactory consensus accuracy [29], we further phased and polished the CLR-Falcon assembly using Falcon-Unzip [23]. The final CLR-Falcon-Unzip assembly had a QV score of 38.86, with k-mer completeness of 97.64%. Both measurements were still worse than those achieved by HiCanu and hifiasm with HiFi reads, while using much less computing time and resources.

In addition, the variable and larger than expected total assembled genome size of 1.2 Gbp or higher (Figure 1 a, Supplementary table 1 and 2), the BUSCO duplication rates varied but remained high (Supplementary table 1 and 2), underlining the difficulty of assembling the highly heterozygous and repetitive cassava genome [8–10]. For the HiCanu and hifiasm TME204 assemblies, the total sizes were both about twice the expected haploid genome size, and the BUSCO duplicate scores were above 80% (Supplementary table 2). Falcon (HiFi and CLRs) and IPA produced smaller assemblies, with lower BUSCO duplicate scores. Merqury k-mer analysis [28] confirmed that the different long

reads and assemblers varied in their performance of resolving haplotypes in the TME204 genome, with HiFi reads in combination with hifiasm producing the most haplotype-resolved assembly, and CLR-Falcon the least (Figure 1 g).

Phased, haplotype-resolved contigs of cassava TME204

Subsequently we used a newer release of the hifiasm assembler (v0.15.2) to assemble the haplotype-resolved TME204 contigs, which were also phased at the same time using Hi-C technology (Table 2). The resulting two sets of haplotigs (phased haplotype-resolved contigs) represent haplotype 1 (762 Mbp) and haplotype 2 (706 Mbp) of the diploid cassava genome, hereafter referred to as H1 and H2, respectively. The assembly has a QV score of 45.23 for H1 haplotigs, 48.94 for H2 haplotigs, and 46.63 for the combined set of sequences. For each haplotype and the combined diploid assembly, the k-mer completeness is 79.6%, 79.1%, and 98.4%, respectively, indicating that about 19% of the k-mers were haplotype-specific. Most importantly, the majority of haplotype-specific k-mers are present only once in the assembled sequences, while the majority of homozygous k-mers shared by two haploid genomes are present twice (Figure 2). This would be expected for a completely haplotype-resolved genome assembly in which even homozygous segments of the genome are included in both haplotypes. Only 3% of k-mers were from artificial duplications (Figure 2), which was similar to the false duplication rates measured at approximately 1% (reference asmgene score) to 4% (BUSCO duplication score). Functional completeness measured using plant BUSCO orthologs and TME204 Iso-Seq transcripts was 98% and above. The reference asmgene completeness score was slightly lower (95%), which could be due to the high level of sequence differences between AM560 and TME204 (see later comparative analysis), therefore fewer AM560 reference genes could be aligned to TME204, resulting in lower asmgene completeness and duplication scores.

To assess the structural accuracy of the assembled TME204 haplotigs, we mapped the longer PacBio CLR sequences from the same DNA sample (Table 1) to the haplotigs and analyzed the CLR read coverage along each haplotig (Supplementary figure 2). We defined reliably assembled sequences as those with at least 10x CLR read coverage. More than 97% of the assembled bases could be classified as correctly assembled with this quality metric.

Table 2. Assembly quality assessment of Cassava TME204 haplotigs generated by hifiasm v0.15.3.

Quality Category	Quality Metric	Haplotype 1	Haplotype 2
General	Contig Size (Mbp)	762	706
Continuity	Contig N50 (Mbp)	18	26
	Contig NG50 (Mbp)	18	22
	Largest contig (Mbp)	41	44
Base accuracy	QV [a]	45.23	48.94
	k-mer completeness (%) [a]	79.6	79.1
Structural accuracy	k-mer false duplications (%) [a]	3.0	2.4
	BUSCO duplicate (%)	4.9	4.2
	Reference asmgene duplicate (%)	1.6	0.8
	Reference gene transfer duplicate (%) [b]	3.8	3.2
	Reliable blocks (%)	97.2	97.5
	Congruent genetic markers (%)	99.8	99.9
Functional completeness	BUSCO complete (%)	99.0	98.8
	Reference asmgene complete (%)	95.2	95.4
	Reference gene transfer rate (%) [b]	96.3	96.5
	Transcript alignment rate (%)	99.3	99.4

[a] QV and k-mer completeness for the combined assembly is 46.63 and 98.4%, respectively. The k-mer false duplication rate for the combined assembly is 3.5%.

[b] Calculated based on the number of lifted genes regardless of completeness

To further validate the base, structural, and phasing accuracy of TME204 haplotigs, we generated complete sequences (96 to 128 Kbp) of bacterial artificial chromosomes (BACs) containing TME204 genome fragments and aligned them to both sets of haplotigs (Figure 3, Supplementary figure 2). When a region is properly assembled and phased, we expect one continuous BAC-to-haplotig alignment for the corresponding BAC (resolved BAC). Three of the four sequenced BACs were resolved in H1 and one was resolved in H2, either perfectly or with only one indel difference (Supplementary table 4), confirming the close to Q50 consensus accuracy (i.e. one error per 100 Kbp consensus sequences). The striking differences of BAC-to-haplotig alignments between the two haplotypes highlight the high level of haplotype differences in these regions.

The haplotigs were also compared with the cassava high-density genetic map [30]. Among the 22,403 available genetic makers, about 14,000 could be uniquely aligned to each set of the TME204 haplotigs perfectly (i.e. with full length coverage and 100% sequence identity). More than 99.8% of these unique and perfect genetic markers showed high congruence between the genetic map and assembled haplotigs (Supplementary figure 3). Only less than 0.2% of the genetic markers were found among markers with different chromosome origins. Plots of genetic versus physical distance identified three pairs of chromosome-scale haplotigs (chromosomes VIII, XII and XIV) and six other chromosome-scale haplotigs in either H1 or H2 (Table 3). In plots of genetic versus physical distance for these chromosome-scale haplotigs (Supplementary figure 3), we often observed steep slopes at the haplotig ends and flat regions in their centers, which is consistent with increased recombination in chromosome arms and reduced recombination in pericentric regions of the chromosomes. Collectively, the data suggest that all of the 18 cassava chromosome pairs are already highly continuous at the haplotig level, where each chromosome is composed of only one to a few haplotigs.

Pseudochromosome pairs of cassava TME204

To further scaffold haplotigs into pseudochromosomes, we first used Hi-C scaffolding, but this did not further scaffold any haplotigs in H1 (Supplementary file 1). In H2, Hi-C data produced seven chromosomal scaffolds that were perfectly congruent with the genetic map, but also mis-joined haplotigs from different chromosomes (Supplementary table 5, Methods). Together, the high congruence between haplotigs and the genetic map allowed us to reconstruct all 18 pairs of pseudochromosomes with high confidence (Figure 4, Supplementary file 2). TME204 H1 and H2 pseudochromosomes are composed of 43 and 39 haplotigs, respectively. In total 12 pseudochromosomes are chromosome scale (Table 3). Haplotig orientations could be determined (Supplementary file 3) except for two small haplotigs in H2, representing the first 0.4 Mbp of chromosome VII and the last 1.3 Mbp of chromosome XI (Supplementary file 4). Together, 86.8% and 91.1% of the haplotig sequences could be assigned to chromosomes for H1 and H2, respectively (Table 2, Supplementary files 3 and 4).

Table 3. Phased chromosome pairs in TME204 diploid genome assembly.

Chromosome	Haplotype 1			Haplotype 2		
	Length (Mbp)	Haplotigs (n)	Reference gene transfer rate (%) [a]	Length (Mbp)	Haplotigs (n)	Reference gene transfer rate (%) [a]
I	44.60	5	97.8	43.73	1	98.1
II	39.76	2	96.2	40.67	2	97.9
III	34.10	2	95.5	33.73	2	96.1
IV	35.07	1	97.2	35.26	3	93.6
V	33.99	1	97.3	33.40	2	97.7
VI	32.06	2	95	32.36	3	96
VII	37.89	6	92.5	36.62	5	91.5
VIII	40.94	1	97	42.44	1	97.1
IX	39.49	3	95.5	36.52	5	94.4
X	33.53	3	93.3	31.79	1	94.1
XI	34.31	2	94.9	33.74	2	95.4
XII	40.28	1	96.6	38.12	1	96.4
XIII	39.96	2	94.9	38.46	1	95.6

XIV	31.29	1	97.1	29.54	1	96.4
XV	35.50	2	97.8	34.23	2	97.9
XVI	34.02	1	95.6	34.23	2	95.9
XVII	37.53	3	93.1	33.98	2	92.1
XVIII	37.65	3	91.4	34.55	3	93.5
Total number of haplotigs		1,439		770		
Total length of haplotigs (bp)		762,392,783		706,328,643		
Total number of anchored haplotigs		43		39		
Total length of pseudochromosomes (bp)		661,977,943		643,362,786		
Number of unanchored haplotigs		1,396		731		
Number (%) of unanchored haplotigs aligned to pseudochromosomes		1,154 (82.7)		688 (94.1)		
Length of unanchored haplotigs (bp)		100,414,840		62,965,857		
Length (%) of unanchored haplotigs aligned to pseudochromosomes		63,212,546 (63.0)		45,142,471 (71.7)		
Annotated genes in unanchored haplotigs (% duplicate)		374 (80.6)		443 (69.1)		
Number of unanchored, mitochondrial haplotigs		281		53		
Length of unanchored, mitochondrial haplotigs		12,170,354		2,557,482		

[a] Percentage of AM560 genes that were lifted to the corresponding chromosome in each TME204 haplotype assembly, regardless of copy number and completeness

In both TME204 H1 and H2 assemblies, we found haplotigs that could not be scaffolded using either the genetic map (Table 3) or Hi-C technology (Supplementary files 1 and 2). A majority of these unanchored haplotigs can be partially aligned to the pseudochromosomes with an average sequence similarity of 98% (Table 3). A few hundred AM560 genes can be transferred onto these haplotigs as

well, although most (70%) were duplicated copies of genes that already transferred onto pseudochromosomes. It is clear that these haplotigs are of cassava origin and not from foreign contamination. When the assembled sequences were screened against the NCBI (National Center for Biotechnology Information) mitochondrial database, unanchored haplotigs representing the highly fragmented mitochondrial genome were identified in both haplotype assemblies (Table 3, Supplementary figure 4 a). When compared to the other non-mitochondrial unanchored haplotigs, mitochondrial haplotigs have a smaller size variation (25-76 Kbp) and lower depth of coverage on average (Supplementary figure 4 b). Regions similar to nuclear mitochondrial pseudogene regions (numt's) were also ubiquitous and found in both pseudochromosomes (Supplementary figure 4 c) and unanchored haplotigs (Supplementary table 6). Some of the non-mitochondrial unanchored haplotigs can be regions still missing from the current set of pseudochromosome pairs where the gene content completeness ranges from 91 to 98% (Table 3). They can also be results of assembly artifacts (i.e. collapsed repeats) or represent novel haplotypes from *de novo* mutations.

Repeat and gene landscape of cassava TME204 genome

De novo repeat modeling using all resolved allelic sequences identified 1,431 repeat families, with 1,016 families representing novel unclassified repeats, which make up 20% of TME204 genome (Supplementary figure 5). The distribution of family sizes and sequence lengths among the novel repeat families are similar to those in LTR families (Supplementary figure 5 b), which make up 38% of TME204 genome. In total, over 60% of each TME204 haploid genome can be masked as repeats, without counting small RNA and low complexity sequences (Supplementary figure 5).

During the last 10 years, continuous efforts have been made to improve the assembly and annotation of the cassava reference genome AM560 [7,12,30]. The set of AM560 reference gene models [31] is widely used in the research field. We therefore first annotated the TME204 genome (annotation release v 1.0) by transferring well established cassava reference gene models to TME204

H1 and H2 assemblies (Table 3). 96-97% of the 32,805 AM560 gene loci could be lifted completely to TME204 H1 and H2 assemblies with a duplication rate of 3 to 4%, which is similar to BUSCO complete and duplicate scores (Table 2). Comparison of orthologous gene pairs revealed high gene synteny (99%) between pseudochromosomes of AM560 and the TME204 H1/H2 assembly. Nine inverted regions involving 109 genes distributing among pseudochromosomes III, VI, VII, VIII, X, and XVIII were found between AM560 and the TME204 H1 assembly. There are seven inversions between the AM560 and TME204 H2 assemblies involving 203 genes on chromosomes VI, VII, X, XI and XVIII (Figure 5 a).

To complement the reference gene models, we also predicted genes (annotation release v 1.1) in TME204 H1 and H2 assemblies using the AUGUSTUS software tool, with experimental evidence from TME204 Iso-Seq transcripts (Supplementary table 7) and RNA-seq data [32] (Supplementary table 8). In contrast to the 53 K protein coding transcripts transferred from reference annotation, *ab initio* gene prediction with extrinsic evidence identified 93 K and 84 K protein coding transcripts in the TME204 H1 and H2 assemblies, respectively. BUSCO scores for the predicted proteomes and assembled genomes were similar, suggesting the functional completeness of the annotated proteomes well represents the underlying genomes (Supplementary figure 6). Figure 5 b shows that 93% of the previously transferred gene loci overlapped with *ab initio* predicted gene models, indicating a good recall rate of the *ab initio* gene prediction process. Among the more than 30 K predicted novel gene loci per assembly, which do not overlap with any transferred reference genes, 27 K are distributed uniformly across the 18 pseudochromosomes (Figure 5 c). The other 21 K and 13 K arise from unanchored haplotigs in the TME204 H1 and H2 assemblies, respectively (Figure 5 d). Close to 80% of the chromosomal predicted transcripts (both novel and known) could be functionally annotated using the InterPro protein database, while only 10% of the novel predicted transcripts from unanchored haplotigs showed significant matches to InterPro protein families (Figure 5 e). Novel predicted genes on chromosomes and unanchored haplotigs are functionally

distinct. Chromosomal novel genes are enriched for GO biological process (BP) term “cellular components of DNA packaging complex”, including nucleosome, chromatin, chromosome and DNA-protein complex (Figure 5 f). Novel genes from unanchored haplotigs are enriched for GO BP term “cellular components of chloroplast thylakoids” (Figure 5 g). Similarly, other GO BP terms were enriched as well (Supplementary figure 7 a and b). Chromosomal novel genes are enriched for “chromatin organization”, “meristem development and maintenance”, and “cell response to stress and stimuli”. Novel genes on unanchored haplotigs are enriched for “cytochrome complex assembly” and “related metabolism processes”.

Tissue-specific differentially expressed transcripts

Ab initio genome annotation identified 94 K and 84 K transcripts in TME204 H1 and H2 assemblies, respectively. To construct the haplotype resolved reference transcriptome, identical transcripts between haplotypes and within one haplotype were collapsed and only one copy of the sequences was kept, yielding 147,503 unique transcript sequences. More than 81% of predicted transcripts have different sequences between haplotypes (Figure 6 a). In such cases, analyzing RNA-seq data using one haploid set of genes/transcripts as the reference could potentially miss haplotype-specific, novel expression patterns. Therefore we re-analyzed previously published [32] RNA-seq data (Supplementary table 8) generated from nine different tissues of TME204. Based on the newly calculated transcript expression values, biological replicates cluster closely together in the PCA analysis of (Figure 6 b), which is similar to the previous analysis result based on the AM560 reference genome [32]. Across the nine tissues, 60,839 transcripts (41%) were expressed (i.e. at least two replicates per pairwise comparison have TPM value 1 and above). Among all pairwise comparisons against stem, in total 9,437(6%) transcripts showed significant difference in expression (a fold change (FC) of $\log2|FC| > 2$, adjusted p-value < 0.00001) (Figure 6 c). Clustering analysis of expression values of the differentially expressed transcripts (DETs) grouped the samples into two major distinct clusters: storage root, fibrous root, and root apical meristem (RAM) in one cluster,

shoot apical meristem (SAM), lateral bud, leaf, midvein, petiole and stem in the other. The sample clustering is consistent with the previous result as well [32]. When comparing single-copy with multi-copy transcripts (i.e. same transcripts from duplicated gene loci within one haplotype), multi-copy transcripts have a relatively lower fraction of expressed transcripts and DETs (Figure 6 a). This is consistent with previous findings [33] that single copy genes are generally more highly expressed than multi-copy genes. Transcripts from novel predicted gene loci on chromosomes are also less expressed and differentially regulated in different tissues (Figure 6 d). Classified by the haplotype origin of the transcripts, only 141 DETs are collapsed homozygous isoforms between haplotypes, 4651 are from the H1 assembly and 4644 are H2-specific. Functional enrichment analysis of the three sets of DETs identifies a common GO BP term “developmental process”, which is expected since different tissues are compared here. DETs from H1 contribute to more diverse biological processes than H2 DETs (Figure 6 e), with the most significantly enriched BP terms being “transcription regulation, DNA templated”, and “photosynthesis, light harvesting”, which are also enriched in H2 DETs. The DETs between haplotypes do have different functions during tissue development. Our analysis also identifies 663 DETs from novel predicted gene loci on chromosomes. Functional enrichment analysis reveals that these novel DETs are mainly involved in biological processes of “response to stress, stimuli, and defense”, “DNA modification”, “methylation/demethylation”, and “protein phosphorylation” (Figure 6 f).

Isoform allele-specific expression

For isoforms that are common to both haplotypes, we further investigated allele-specific expression (ASE) differences between the 39,028 bi-allelic transcripts from 35,264 orthologous gene loci (see Methods). This is the largest set of bi-allelic transcripts analyzed for cassava so far [8,9]. Most of these bi-allelic pairs maintained high levels of coding sequence similarity (Figure 7 a). In each tissue (Supplementary table 8), about 20-30% of the expressed alleles showed significant (adjusted p-value < 0.05) differences in expression between allelic pairs, within the range of previously reported values

of 14% [8] and 34% [9]. The ASE differences are mostly small to median fold changes (less than |8| fold). RAM has the least number of alleles with ASE differences, while fibrous root, midvein and petiole have the most abundant alleles with ASE differences (Figure 7 b, Supplementary figure 8 a). When alleles with ASE biases were compared across all nine tissues, about 8% were consistent such that the ASE was biased towards one allele in all tissues, 33% were inconsistent and tissue-specific, and the rest were inconsistent and the ASE bias persists only in some but not all tissues (Supplementary figure 8 a). Among ASE transcripts in the three more closely related subterranean tissues (storage root, fibrous root, and RAM), 20% were consistent, 52% were inconsistent and tissue specific, 1.6% were inconsistent and showed direction-shifting (Figure 7 c). Across the more distantly-related tissues, fibrous root, midvein and petiole, which harbor the most abundant ASE transcripts, similar distributions were observed and the fraction of inconsistent ASE transcripts with direction-shifting was around 2% (Supplementary figure 8 b). The number of up-regulated alleles between allelic chromosome pairs was similar across the genome, suggesting the allelic expression tends to be balanced between haplotypes (Figure 7 d).

Intra- and inter-genomic diversity of cassava genomes

Based on k-mer analysis, each TME204 haplotype harbors close to 20% of haplotype-specific k-mers. However, analysis of orthologous pairs of coding sequences revealed high gene synteny and coding sequence similarity on average. To systematically investigate sequence differences between the TME204 haplotypes and between cassava cultivars, different methods were applied. In the first approach, we produced reliable alignments between assembled sequences longer than 500 bp, with exact matches >100 bp [34,35]. With this method, 24%-29% of sequences per haploid genome were too divergent to be aligned and thus not accounted for the comparative analysis (Table 4). Between the two TME204 haploid genomes, the average level of sequence differences was 1.12%, including 2,526,852 SNPs and 1,733,059 single nucleotide indels, 13,332 small indels (20-50 bp) and 13,213 large indels (50 -10,000 bp). 67% of the large indels were expansion/contraction of repetitive

elements, while only 3% of the small indels were of the same types (Table 4, Supplementary figure 9). The levels and characteristics of inter-genomic differences between the two cassava cultivars (TME204 vs. AM560) were similar to those within TME204 diploid genome (Table 4, Supplementary figure 9).

Table 4. Inter- and intra-genomic diversity of cassava revealed by comparative analysis of assembled contig sequences.

	Inter-genomic	Inter-genomic	Intra-genomic
Reference	AM560	AM560	TME204 H1
Query	TME204 H1	TME204 H2	TME204 H2
Too divergent to be aligned (Mbp)	220 (29%)	186 (26%)	181 (24%)
Uniquely aligned (Mbp)	387	393	420
Sequence similarity (%) in uniquely aligned regions	98.75	98.79	98.85
Number of SNPs	2,720,699	2,679,237	2,720,467
Number of single-nucleotide indels	1,874,181	1,855,394	1,867,232
Number of Assemblytics small indels (20 – 50 bp) (% expansion/contraction of repeats)	13,605 (3%)	13,467 (3%)	13,332 (3%)
Number of Assemblytics large indels (50 bp – 10 Kbp) (% expansion/contraction of repeats)	13,387 (67%)	13,073 (66%)	13,213 (67%)

As a complementary approach, we also compared HiFi reads directly to AM560 contigs and TME204 haplotigs, which identified not only indels, but also inversions and breakpoints of other complex SVs such as translocations, etc. If the TME204 genome was assembled error-free, all sequence variants between one TME204 haplotype assembly and HiFi reads would have been heterozygous and representing intra-genomic diversity. Indeed, only less than 1% of structural variants (SVs) reported by HiFi read alignments were homozygous. They could have resulted from mis-assemblies and/or mis-alignments. Most of the SVs (> 99%, 115,000) were heterozygous between TME204 haplotypes, confirming that the TME204 haplotigs are structurally accurate and do harbor a high level of intra-genomic sequence differences between haplotypes. The very high number of reported SVs was due to the high sensitivity of the analysis method, since SVs supported by three or more HiFi reads could be identified with high confidence. Similarly, between the TME204 diploid genome and the AM560 genome, 198,000 SVs were identified by the HiFi read alignments, of which 70.5% were heterozygous and thus specific to only one of the TME204 haplotypes (Figure 8 a). On average, the number of SVs between one TME204 haplotype and AM560 haploid genome reached 128,000, which is again very similar to the number of intra-genomic SVs (115,000) between TME204 haplotypes. In addition to the much higher sensitivity, analysis of HiFi read alignments was also able to identify very small inversions such as those from 100 bp to a few Kbp (Figure 8 b), which were not captured by gene synteny analysis. Consequently, the number of inversions reported with this method was much higher and not directly comparable with the counts from gene synteny analysis.

We also compared the TME204/AM560 pseudochromosome pairs by identifying and examining regions that shared information content [36], which is more robust in comparing sequences with low sequence identity and where the linear order of homologs is not preserved [37]. The analysis revealed that each cassava pseudochromosome consists of islands of conserved regions flanked by regions with more degenerated sequences. Although the order of these conserved regions was mostly kept between each pseudochromosome pair, extensive genomic rearrangements still exist

(Figure 9). In total, more than 2,500 inversions were detected between each pair of cassava haploid genomes with this method (SupplementaryDataFile.pdf).

Cassava pan-genome

The presence of haplotype-specific k-mers and abundant SVs between the cassava haploid assemblies suggests that any of the linear reference genomes of one haplotype, either the AM560 pseudo-haplotype or TME204 H1 or H2, cannot represent the sequence diversity of cassava populations and may miss haplotype-specific sequences. To overcome this limitation, we built a pan-genome graph from TME204 H1 and H2, and also one including the reference AM560 pseudo-haplotype. Starting with each initial reference haplotype (TME204 H1 or AM560), haplotype-specific large SVs (100 bp and 100 Kbp) were identified in the query haplotype and subsequently amended to the reference haplotype for pan-genome graph reconstruction. We found 114,773,684 bases representing 40,776 such large SVs in TME204 H2 that were divergent from TME204 H1 (Figure 10 a). In comparison to the linear TME204 H1/H2 as the only reference genome, using the TME204 pan-genome as reference allowed us to map more Illumina reads from the same TME204 sample with higher accuracy (i.e. mapping quality 20 and above) (Figure 10 b). In the pan-genome that includes the AM560 genome and the two TME204 haplotypes, we found 198,028,264 bases representing 53,098 large SVs in the two TME204 haplotypes that were divergent from AM560. As reported above by the Assemblytics analysis, where a majority of large indels (50 bp – 10 Kbp) are expansion/contraction of repeats, the SV harboring divergent sequences in both pan-genomes are enriched for repeats, especially LTR elements (Figure 10 c), suggesting that most SVs captured by pan-genome graphs are LTR retrotransposons related.

Discussion

By comparing PacBio CLR and HiFi sequencing technologies and benchmarking four HiFi assemblers [15,23,24], we demonstrate that HiFi reads are extremely effective in producing a nearly complete

and accurate haplotype-resolved assembly of the complex diploid cassava genome. The combination of high base accuracy and long read length greatly simplified the data analysis workflow, decreased data footprints, shortened data analysis time, and improved the assembly quality. CLR-Falcon assembly starts with read self-correction, which is not only computationally expensive, but can also mix reads from different haplotype alleles, paralogous gene members, or repetitive elements. In contrast, HiFi reads have higher resolution and accuracy in resolving these sequence variants. All HiFi TME204 assemblies reached consensus accuracy between Q40 (99.99%) and Q50 (99.999%). The CLR-Falcon contig sequences were less accurate even after extensive polishing using signal level data, which also has the risk of introducing novel errors because current polishing pipelines cannot accurately differentiate reads from different haplotype alleles and repeat copies [25].

Among the compared HiFi assemblers, hifiasm generated the most completely haplotype-resolved TME204 genome assembly. The haplotigs reached NG50 of 18 Mbp, with consensus accuracy of QV45. Three pairs of pseudochromosomes are haplotigs without sequencing gaps. These values satisfy the 6.7.Q40 and 7.C.Q50 genome assembly quality metrics, which are measures for close-to-finished genome qualities as proposed by the VGP consortium [25]. In comparison to a recently published correspondence on a chromosome level-phased assembly of cassava cultivar SC205 [9], the TME204 HiFi assembly is more continuous (contig N50 18 Mbp vs 1.1 Mbp in SC205) and more complete (BUSCO complete score of 99% instead of 88% in SC205). The high accuracy and completeness of assembled sequences improved gene annotation, with 20 K novel chromosomal gene loci being predicted with experimental evidence of Iso-Seq transcripts and RNA-seq data. A total of 35,264 bi-allelic gene loci were phased, making the TME204 assembly the most haplotype-resolved cassava genome so far, in comparison to 24,128 in SC205, and 18,723 in 60444. ASE analysis of this largest set of bi-allelic transcripts across nine TME204 tissues confirmed that most alleles are coordinately expressed during tissue development, as previously reported for cassava [8,9] and recently reported for ginger [38]. On average, 20-30% of the expressed transcripts showed

ASE differences in at least one tissue. The expression bias is often inconsistent and tissue-specific. AES bias with direction-shifting was observed in 2% of the expressed transcripts. This is also similar to the patterns observed in ginger and tea plant [38,39]. RNA-seq data analysis against the reference transcriptome where haplotype origin of transcripts is known also enabled us to determine that DETs from different haplotypes may play different roles during cassava tissue development. However, the current TME204 H1 and H2 assembly is still a random mixture of different parental chromosomes because with Hi-C technology alone it is not possible to phase across chromosomes [19]. Trio-binning [40] using two parental genomes will be needed to completely separate parental chromosomes in the offspring genome and to assist in the analysis of monoallelic expression of parentally imprinted genes in offspring. Between allelic chromosome pairs, the number of ASE transcripts biased towards each haplotype was similar across the genome (Figure 7 d). Finding ASE transcripts will thus still hold true after reshuffling of pseudochromosomes between haplotypes. The number of DETs between allelic chromosome pairs was also similar (Supplementary figure 10 a). Functional enrichment analysis of DETs per chromosome identified chromosomes with uniquely enriched BP terms (Supplementary figure 10 a and b). The existence of such chromosomes suggests that haplotype-specific enriched functions in DETs will hold true no matter how the chromosomes are shuffled between haplotypes, although the specific set of DETs and actual enriched GO terms per haploid genome will change. Given the high level of sequence accuracy, functional completeness, and haplotype resolution, the TME204 genome and transcriptome will be a powerful resources and tools for establishing new technologies, such as novel marker identifications and genome editing for cassava trait improvement and breeding.

The HiFi sequencing strategy in combination with Hi-C not only enabled the assembly of haplotype-resolved chromosome pairs, but also allowed reconstruction of over 300 mitochondrial haplotigs

with lengths varying between 25 to 76 Kbp. Plant mitochondrial genomes are known to be highly fragmented, with total lengths varied from 200 to 2,000 Kbp [41]. The 53 mitochondrial haplotigs in the TME204 H2 assembly added up to a total size of 2 Mbp (Table 3), which can represent a complete mitochondrial genome. Interestingly, there were still 281 mitochondrial haplotigs (with a total length of 12 Mbp) in the TME204 H1 assembly, suggesting the presence of different sequence variants of the mitochondrial genome. This result strongly supports the recent discovery of plant mitochondrial genomes as a complex and dynamic mixture of sequence variants [42]. It signifies that the highly accurate base information over very long stretches of DNA molecules provided by the combination of HiFi sequencing with Hi-C technology is powerful in resolving the complexity of multiple haplotypes and isoforms, which will revolutionize and fundamentally improve future assemblies of plant genomes.

Extensive SVs and divergent sequences per haploid genome are dispersed throughout both TME204 haplotypes, and the levels of intra-genomic (TME204) and inter-genomic (TME204 versus AM560) diversity are similar in cassava. Genome regions with SVs are enriched with repeats, especially LTR elements. Accumulation of SVs and hemizygous sequences have been recently reported for other crops such as grapes, potatoes, and rice, and are considered a major force contributing to the cost of domestication [20,43,44]. Analysis of SVs in cassava TME204 population samples will help to reveal to what extent SV is driving cassava genome evolution. Our study demonstrates that reference-guided analysis of HiFi read alignment is more sensitive in identifying SVs than comparative analysis of assembled consensus sequences, and thus HiFi sequencing will be a cost-effective method for population scale analysis of SVs.

The high degree of genomic variations in cassava cultivars also highlights the importance of building a pan-genome [45–48] for research and breeding. Under-representation of genetic diversity by any linear haploid cassava genome will limit our understanding of genetic variations in reference-guided

analysis, especially when samples are sequenced using Illumina short reads, for example in genotyping-by-sequencing and RNA-seq experiments. Haplotype-specific short reads may remain unmapped, thus important genome information may be left undiscovered. Technically, large SVs are a frequent source of errors in aligning short Illumina reads, which may lead to mis-interpretation of data [49]. We demonstrate that using a pan-genome reference did increase mapping rate and mapping quality of Illumina reads in comparison to using a conventional linear haploid reference. Detailed investigation of a cassava pan-genome, including more cultivars, and its influence on interpretations of omics data is on-going and will be reported in the near future.

Potential implications

Using the HiFi sequencing strategy in combination with Hi-C, we reconstructed two chromosome-scale haploid genomes for the diploid cassava TME204 with the highest accuracy and completeness achieved so far, which allowed us to study the sequence, gene content, gene expression, and genome structure with unprecedented resolution. The haplotype resolved genome and transcriptome will be a valuable resource for cassava breeding and research. The ability to resolve the high complexity of multiple haplotypes and isoforms demonstrated in our study will provide insights for future work on plant genomics.

Methods

DNA extraction and Illumina shotgun sequencing

Leaves were collected from 6- to 8-week old *in vitro*-grown TME204 plants. Genomic DNA was extracted using DNeasy Plant Mini Kit (QIAGEN). The TruSeq DNA Nano Sample Prep Kit v2 (Illumina) was used for library preparations according to the manufacturer's instructions (Supplementary methods). The NovaSeq 6000 (Illumina NovaSeq 6000 Sequencing System, RRID:SCR_016387) was used for cluster generation and sequencing according to the standard protocol for paired-end (PE) sequencing at 2 X150 bp.

High molecular weight DNA extraction

Fresh leaves were harvested from *in vitro*-grown TME204 plants kept in the dark for 12-24 hours pre-harvest, and the petiole and basal midrib were removed with a sterile pair of scissors. One gram of leaf tissue was then snap-frozen in liquid nitrogen and homogenized to a powder with a mortar and pestle. Lysis buffer (9.5 mL of G2 buffer from the Blood & Cell Culture DNA Midi Kit (QIAGEN) and 19 µL of RNase A (100 mg/mL, Sigma Aldrich) was added to the homogenized tissue in a 50 mL conical centrifuge tube (Falcon). 500 µL of Proteinase K (20 mg/mL, Roche) was then added to the sample and the mixture was vortexed for 10 seconds. The sample was incubated at 50°C (Memmert Incubator) on a lab roller for 3 hours. Afterwards the sample was centrifuged for 10 minutes at 20°C at 1,800 x g. The supernatant was then used for high molecular weight (HMW) genomic DNA extraction according to the Genomic-tips protocol (100/G, Blood & Cell Culture DNA Midi Kit, QIAGEN).

PacBio CLR and HiFi library preparation and sequencing

The concentration of HMW genomic DNA was measured using a Qubit Fluorometer dsDNA Broad Range assay (Thermo Fisher Scientific). The CLR and HiFi library preparations started with 8 µg and 15 µg HMW DNA, respectively, using the SMRTbell Express Template Prep Kit 2.0 (Pacific Biosciences) according to the manufacturer's instructions (Supplementary methods). The CLR SMRT bell template-polymerase complex was sequenced on a PacBio Sequel instrument using the Sequel Sequencing Kit 3.0 (Pacific Biosciences) with six Sequel™ SMRT® Cells 1M v3 (Pacific Biosciences), taking a 10-hour movie per cell. The HiFi SMRT bell template-polymerase complex was sequenced on a PacBio Sequel II instrument (PacBio Sequel II System, RRID:SCR_017990) using the Sequel II Sequencing Kit 2.0 (Pacific Biosciences) and one Sequel™ II SMRT Cell 8M (Pacific Biosciences), taking a 30-hour movie.

Hi-C library preparation and sequencing

Two grams of fresh leaf tissue was harvested from *in vitro*-grown TME204 plants and flash-frozen in liquid nitrogen. The leaf tissue was then shipped on dry ice to Arima Genomics (San Diego, USA) for Hi-C library preparation (Supplementary methods). The DNA library was sequenced by Arima on the Illumina HiSeq X (Illumina HiSeq X Ten, RRID:SCR_016385) following manufacturer's protocols, yielding 727,211,240 read pairs (2X150 bp) (Accession number: ERR5484651).

RNA isolation, PacBio Iso-Seq library preparation and sequencing

Three different tissues were collected from greenhouse-grown TME204 plants: the top five leaves with petioles, apical and lateral meristems including the stem, and fibrous roots. The various tissues were flash-frozen in liquid nitrogen and homogenized with a mortar and pestle. RNA was isolated with the Spectrum Plant Total RNA kit (Sigma-Aldrich) according to Protocol A. The quantity and quality of total RNA samples were measured using Qubit RNA BR Assay Kit (Thermo Fisher Scientific) and Agilent TapeStation 4200 with RNA-specific tapes (Agilent Technologies), respectively. Samples with RNA integrity numbers ≥ 7 were used for Iso-Seq library preparation and sequencing.

PacBio Iso-Seq templates were prepared using the NEBNext Single Cell/Low Input cDNA Synthesis & Amplification Module (New England BioLabs) and PacBio Iso-Seq Express Template Switching Oligos (TSO) (Pacific Biosciences), following the PacBio Iso-Seq protocol “Procedure & Checklist – Iso-Seq Express Template Preparation for Sequel and Sequel II Systems” (PN 101-763-800) (Supplementary methods). The Iso-seq SMRT bell template-polymerase complex was sequenced on a PacBio Sequel II instrument using Sequel II Sequencing Kit 2.0 (Pacific Biosciences) and single Sequel™ II SMRT Cell 8M (Pacific Biosciences) taking a 30-hour movie.

Bacterial artificial chromosome (BAC) clone library construction, screening, sequencing and assembly

High molecular weight (HMW) DNA was prepared from TME204 young leaves as previously described [50,51]. Agarose embedded HMW DNA was partially digested with HindIII (New England Biolabs), sized through two size selection steps by pulsed field gel electrophoresis (CHEF Mapper system, Bio-Rad Laboratories) and ligated into the pAGIBAC-5 HindIII-Cloning vector. Pulsed-field migration programs, electrophoresis buffer and ligation desalting conditions were done according to [52]. The insert size of the BAC clones was assessed using the FastNot I restriction enzyme (New England Biolabs) and analyzed by pulsed field gel electrophoresis. Colony picking was carried out using a robotic workstation QPix2 XT (Molecular Devices) using a white/blue selection. White colonies were arranged in 144 384-well (55,296 BAC clones) microtiter plates containing LB medium with chloramphenicol (12.5 µg/mL) supplemented with 6% (v/v) glycerol.

Individual BAC clones were selected using radiolabeled ([α-33P]dCTP) probes. DNA were extracted from individual clone using Nucleobond Xtra midi kit (Macherey-Nagel) and used for PacBio library preparation by The French Plant Genomic Resources Center (CNRGV) of the French National Research Institute for Agriculture, food and Environment (INRAE). PacBio sequencing was performed on the Sequel II system with a movie time of 30 hours with 120 min pre-extension step by Gentyane Genomic Platform (INRAE). Circular consensus sequence (CCS) reads per BAC clone were generated using SMRT Analysis Software (SMRT Link v9.0.0, RRID:SCR_002942), and assembled using hifiasm (v0.12.0; RRID:SCR_021069). More details on BAC clone screening, sequencing and assembly can be found in supplementary methods.

Sequencing data quality control

The technical quality and potential sample contamination in Illumina PE reads were evaluated using FastQC (v 0.11.8, RRID:SCR_014583) and FastQ Screen (v 0.11.1, RRID:SCR_000141), respectively. The technical quality of PacBio raw data was checked using the “QC module” in the PacBio SMRT Analysis Software (SMRT Link version 8.0, RRID:SCR_002942). Iso-Seq reads were clustered into high quality (accuracy 99.9%, HQ) transcripts using the “Iso-Seq Analysis” Application in PacBio SMRT

Analysis Software (SMRT Link v10.1.0.119588). The technical quality of Hi-C data was checked using HiCUP (v0.8.0; RRID:SCR_005569)[53].

Estimation of genome properties

Genome complexities such as repeat content and the level of heterozygosity were evaluated with k-mers in the Illumina PE reads using Preqc in SGA (v 0.10.15, RRID:SCR_001982)) [26,54]. Analyzed datasets and their accessions are: Human (ERR091571-ERR091574) [26], cassava AM560 (SRR2847385), cassava TME204 (ERR5484652), cassava 60444 (ERR5484654) (8), cassava TME3 (ERR5484653) (8).

PacBio CLR and HiFi whole genome assembly

PacBio CLR reads were assembled using Falcon (RRID:SCR_016089) [23] in pb-assembly (v0.06). PacBio HiFi reads were assembled using multiple HiFi specific assemblers, including Falcon in pb-assembly (v0.0.8), Improved Phased Assembler IPA (v1.0.5)[55], hifiasm (v0.7, RRID:SCR_021069) [19], and HiCanu (v2.0, RRID:SCR_015880) [15]. Default options were used unless otherwise noted. Improved phased assembly (IPA) was run with both phasing and polishing included.

Benchmarking analysis of assembly accuracy and completeness

Assembly statistics were collected using QUAST (v4.5; RRID:SCR_001228) [56]. NG50 [57] was calculated using the haploid genome size of 750 Mbp. Base-level accuracy and completeness was measured using both mapping-based method and alignment free, k-mer-based method Merqury (v1.1) [28] (Supplementary methods).

For evaluation of structural accuracy, Merqury k-mer analysis results were first used to compute false duplication rates, where k-mers that appeared more than twice in each haploid assembly were used to identify artificial duplications. PacBio CLR reads were then aligned to each haploid genome

and the coverage was analyzed using Asset software [58]. Assembled regions supported by 10 and more PacBio CLR reads were identified as reliable regions.

Functional completeness was measured using BUSCO (v5; RRID:SCR_015008) completeness of single-copy orthologs discovered in plants (Viridiplantae Odb10) [59], and alignment rates of reference genes and TME204 Iso-Seq transcripts. The AM560 reference genome (v8.0) and gene annotation (v8.1) [31] were downloaded from JGI Phytozome 13 [60]. Reference coding sequences (CDSs) were aligned to TME204 haplotigs using minimap2 (v2.15r905, -cxsplice -C5; RRID:SCR_018550) [61]. "asmgene" completeness and duplication scores [19] were calculated using the "paftools" script from the minimap2 package, based on CDSs mapped at ≥97% identity over ≥99% of the CDS length. Iso-Seq data collected from TME204 transcriptomes of fibrous root, stem meristems and leaves were spliced aligned using minimap2 (v2.15r905, x splice:hq). Alignment statistics were collected using alignqc [62].

Haplotype-resolved, phased contig assembly using HiFi reads integrated with Hi-C technology

Two sets of haplotype-resolved, phased contig (haplotig) assemblies were generated using hifiasm (v0.15.3) with a combination of HiFi reads and paired-end Hi-C reads. Haplotigs were first validated against the high density genetic map of cassava [30], which contains 22,403 SNP markers with allele numbers ranging from 2 to 6. Allelic sequences (50 nt upstream sequence + allele sequence + 50 nt downstream sequence) were aligned to haplotigs using BLAT (v3.2.1; RRID:SCR_011919)[63]. For each haplotig, correlation plots of genetic vs. physical distance based on uniquely and perfectly aligned alleles were generated for visual inspection. Sequences of BACs were also aligned to haplotigs using BLAT (v3.2.1). The best BAC-to-haplotig alignment was manually inspected to identify resolved BACs, where one continuous BAC-to-haplotig alignment was produced.

Construction of Pseudochromosomes

Hi-C reads were mapped back to each set of haplotigs independently using the Arima mapping pipeline [64] and were used to further scaffold haplotigs with SALSA2 (v2.2, assisted by the assembly graph, resolved mis-assemblies, five iterations) [65]. No haplotigs in TME204 H1 were further scaffolded with Hi-C data after five rounds of iteration (Supplementary file 1). Thirty haplotigs in TME204 H2 were scaffolded into 13 scaffolds, of which seven were chromosome-scale and consistent with the genetic map (Supplementary table 5). One scaffold was apparently a technical artifact based on genetic markers, reaching 107 Mbp long and joining haplotigs from different chromosomes (I, XVI and XVIII) together (Supplementary table 5). Chromosomes VII, IX and XI were not reconstructed in H2. Because Hi-C scaffolding did not generate results for H1 and the results for H2 were not all satisfactory, ALLMAPS (v0.8.12; RRID:SCR_021171) [66] was used to reconstruct pseudochromosomes for both sets of haplotigs based on the genetic map [30]. Given the observed high congruence between the map and haplotigs, as well as between the map and Hi-C scaffolds, the choice of scaffolding strategy was reasonable and sound.

Repeat modeling, genome masking and annotation

Starting with the assembly of all resolved alleles (i.e. primary plus alternate contigs), repeat elements were predicted using RepeatModeler (v2.0.1; RRID:SCR_015027), with dependency on TRF (v4.09) [67], RECON (v1.08; RRID:SCR_021170) [68], RepeatScout (v1.0.6; RRID:SCR_014653) [69], and RepeatMasker (v4.1.0; RRID:SCR_012954) [70]. Analysis of Long Terminal Repeats (LTRs) were enabled with GenomeTools (v1.5.9; RRID:SCR_016120) [71], LTR_Retriever (v2.9.0; RRID:SCR_017623) [72], Ninja (v0.95-cluster_only) [73], MAFFT (v7.471-with-extensions; RRID:SCR_011811) [74] and CD-HIT (v4.8.1; RRID:SCR_007105) [75]. Among the 1,436 predicted repeat families, 1,021 were unknown/novel according to RepeatClassifier (V2.0.1) [76]. Five predicted repeat families with significant hits to plant genes were identified and removed from the repeat library using ProtExcluder (v1.1) [77] and each TME204 haplotype assembly was then masked using RepeatMasker (-no_is -nolow -norna -xsmall) (v4.1.0).

Genome annotation was first performed by transferring reference gene models from AM560 v8.1 to TME204 haplotype assemblies using liftoff (v1.6.1) [78]. Finding extra copies of the same genes was enabled with a minimum sequence identity of 95% in exons/CDSs. Synteny analysis of orthologous pairs of transferred genes was performed using MCScanX [79] and visually inspected using SynVisio [80].

Complementary to the transferred reference gene models, *ab initio* gene prediction was performed using AUGUSTUS (Augustus, RRID:SCR_008417) [81] with experimental evidence from RNA-seq data, Iso-Seq transcripts and AM560 v8.1 protein/transcript sequences (Supplementary methods). Genomic locations of predicted gene loci were compared to those of transferred gene loci using BEDTools (v2.29.2; RRID:SCR_006646). Predicted protein sequences were compared against AM560 v8.1 protein sequences and Uniprot/Swiss-Prot (release 2021_03) using blastp (v2.10.1+; RRID:SCR_001010), InterPro using InterProScan (v5.52-86.0; RRID:SCR_005829). Best protein matches from AM560 v8.1, Uniprot/Swiss-Prot and InterPro, plus Gene Ontology (GO) terms and pathways were used to functionally annotate predicted genes.

Haplotype resolved transcriptome analysis of differentially expressed transcripts

Transcripts annotated in TME204 H1 and H2 were pooled and de-duplicated using cd-hit-est (v4.8.1) [75] to generate the haplotype-resolved reference transcriptome, for expression quantification and differential expression analysis of transcripts. Transcripts that are identical between TME204 H1 and H2 were counted as homozygous alleles (hom). Transcripts with different sequences between TME204 H1 and H2 were counted as heterozygous alleles (het). Transcripts duplicated within one haploid genome were counted as multi-copy alleles (mc). Transcripts without an identical copy within the same haploid genome were counted as unique/single-copy alleles (sc). Duplicated transcripts (homozygous and/or multi-copy) were collapsed and represented only once in the reference transcriptome. RNA-seq reads from nine tissues with three biological replicates [32] were

mapped to the haplotype-resolved reference transcriptome using kallisto (v0.46.1, un-stranded; RRID:SCR_016582) [82]. All tissues were compared against stem tissue. In each pair-wise comparison of six samples, a transcript was considered to be expressed if the expression value of at least two samples exceeded 1 TPM (transcript per million). Differentially expressed transcripts (DETs) between tissues were identified using DESeq2 (v1.32.0; RRID:SCR_015687) [83] as those with a log2|FC| > 2 and adjusted p-value <0.00001.

Analysis of allele-specific expression

For allele-specific expression (ASE), bi-allelic transcripts were identified by reciprocal blastn (v2.10.0; RRID:SCR_001598) comparison of H1 and H2 transcripts. A unique, bi-directional best matched transcript pair was considered as allele A and B. Expression values for bi-allelic transcripts were a subset from the master quantification table including all resolved alleles. ASE was determined using the same package DEseq2, with adjusted p-value <0.05. ASE transcripts overlapped between tissues and haplotypes were analyzed using upsetR [84].

Comparative genomics

For alignment-based sequence similarity analysis, the cassava reference genome AM560 v8.0 was first disassembled into contig sequences using the utility function "split_scaffold" in IDBA (v1.1.3) [85]. Each set of TME204 haplotigs was then aligned to the AM560 reference contigs and against each other using nucmer (--maxmatch -l 100 -c 500) in MUMmer (v 4.0.0beta2; RRID:SCR_018171) [35], which reported all sequence alignments longer than 500 bp with each exact match longer than 100 bp. Contigs rather than pseudochromosomes were used to prevent false positives when the padding Ns in the query did not match perfectly to the distance in the reference. Sequence alignments were further analyzed using dnadiff in MUMmer [35] and Assemblytics [34] for identification of SNPs, single nucleotide indels, and large indels (20 bp – 10 Kbp).

For structural variant analysis using HiFi reads, TME204 HiFi reads were aligned to reference contigs of AM560 and TME204 haplotigs using minimap2 (v 2.15r905; RRID:SCR_018550) [61]. SVs were called using PacBio structural variant calling and analysis tools PBSV [86]. Summary statistics of SVs were collected using SURVIVOR (v1.0.7) [87].

For chromosome level comparisons, the alignment free method smash++ (v20.04) [36] was used to identify similar/shared regions and genomics rearrangements larger than 10 Kbp between pseudochromosome pairs. Parameters adjusted for analyzing highly repetitive genomes were: filter scale = large, filter size = 50000, filter type = blackman, threshold = 1.0, minimum segment size = 10000.

Pan-genome analysis

Pan-genomes were constructed using minigraph (v 0.15-r426) [49]. Large SVs (100 bp -100 Kbp) were identified and extracted from each pan-genome graph using gfatools (0.4-r214) [88].

Gene ontology (GO) enrichment analysis

For all selected gene sets, GO enrichment analysis was performed using topGO (v2.44.0; RRID:SCR_014798) [89,90] with Fisher exact test p-value cutoff set to 0.00001. The only exception was for the 141 homozygous DETs, where the p-value cutoff was set to 0.001. GO annotation of the *ab initio* predicted gene models were used as the background gene set.

Declarations

Ethics approval and consent to participate

Not applicable.

Data availability

Raw sequencing reads from PacBio (HiFi, CLR and Iso-Seq) and Illumina (Hi-C and shotgun) were deposited in the European Nucleotide Archive (ENA) database under the accession number PRJEB43673 (or ERP127652 as the secondary accession number in ENA). Assembled genome sequences of TME204 H1 and H2 were deposited in the NCBI database under the accession number PRJNA758616 and PRJNA758615, respectively. Assembled BAC clone sequences were deposited in the NCBI GenBank database under the accession numbers MZ959795, MZ959796, MZ959797, and MZ959798. All supporting data and materials are available in the *GigaScience* GigaDB database [91].

List of abbreviations

ASE: allele-specific expression

BAC: bacterial artificial chromosome

BP: biological process

CCS: circular consensus sequence

CDS: coding sequence

CLR: continuous long reads

CMD: Cassava Mosaic Diseases

DE: differentially expressed/differential expression

DET: differentially expressed transcript

ENA: European Nucleotide Archive

GO: gene ontology

HiFi: high-fidelity

HMW: high molecular weight

Indel: insertion and deletion

IPA: improved Phased Assembler

MF: molecular function

NCBI: National Center for Biotechnology Information

numt’s: nuclear mitochondrial pseudogene regions

PacBio: Pacific Biosciences

PE: paired-end

QV: quality value

RAM: root apical meristem

SAM: shoot apical meristem

SMRT: Single Molecule Real-Time

SNP: single nucleotide polymorphism

SV: structural variation

TPM: transcript per million

UDI: Unique Dual Indices

VGP: the Vertebrate Genome Project

Consent for publication

The cassava TME204 (Tropical Manihot esculenta 204) cultivar used in our study was obtained by ETH Zurich from the International Institute of Tropical Agriculture (IITA) in Nigeria in 2003 prior to the implementation of the International Treaty on Plant Genetic Resources for Food and Agriculture (https://www.fao.org/3/i0510e/i0510e.pdf). TME204 has been part of the ETH Zurich cassava germplasm collection since 2003. As a major crop, non-genetically modified cassava, including the wild type TME204 cultivar, is exempt from the Cartagena Protocol on Biosafety to the Convention on Biological Diversity (https://www.cbd.int/doc/legal/cartagena-protocol-en.pdf). The study reported in our manuscript follows all Swiss and international guidelines and legislation.

Competing interests

The authors declare that they have no competing interests.

Funding

This work was supported by the Bill & Melinda Gates Foundation (INV-008213), ETH Zurich, and the Functional Genomics Center Zurich (FGCZ). DP is funded by national funds through FCT (Fundação para a Ciência e a Tecnologia, I.P.) under the Institutional Call to Scientific Employment Stimulus (reference CEECINST/00026/2018). WG is supported by a Yushan Scholarship of the Ministry of Education in Taiwan.

Authors' Contributions

WQ, YL, AP, RS and WG designed the study. YL and CC prepared DNA and RNA samples for sequencing. AP, SG and AB prepared CLR, HiFi and Iso-Seq libraries and performed PacBio sequencing. YL, NR, EP, SV and MF generated the BAC sequences. WQ, YL, PS, DP and WG analyzed

data. WQ, YL, AP, AB, PS, and WG wrote the manuscript. All authors have reviewed the final manuscript before submission and have no competing interests.

Acknowledgments

We thank the high-throughput sequencing team at FGCZ for Illumina sequencing service, Arima Genomics for Hi-C service, Dr. David Stucki and Deborah Moine from PacBio for their technical support, Dr. Haoyu Cheng from Harvard Medical School and Alaina Shumate from the Johns Hopkins University School of Medicine for insightful discussion. We thank Jay Tracy from FGCZ for reviewing the manuscript for English writing and clarity.

Reference

1. Claros MG, Bautista R, Guerrero-Fernández D, Benzerki H, Seoane P, Fernández-Pozo N. Why assembling plant genome sequences is so challenging. *Biology*. 2012; doi: 10.3390/biology1020439.

2. Birky-Jr. CW. Heterozygosity, Heteromorphy, and Phylogenetic Trees in Asexual Eukaryotes. *Genetics*. 144:427–371996;

3. Balloux F, Lehmann L, de Meeûs T. The population genetics of clonal and partially clonal diploids. *Genetics*. 164:1635–442003;

4. Meloni M, Reid A, Caujapé-Castells J, Marrero Á, Fernández-Palacios JM, Mesa-Coelo RA, et al.. Effects of clonality on the genetic variability of rare, insular species: the case of Ruta microcarpa from the Canary Islands. *Ecol Evol*. 2013; doi: 10.1002/ece3.571.

5. Michael TP, VanBuren R. Building near-complete plant genomes. *Curr Opin Plant Biol*. 2020; doi: 10.1016/j.pbi.2019.12.009.

6. Tørresen OK, Star B, Mier P, Andrade-Navarro MA, Bateman A, Jarnot P, et al.. Tandem repeats lead to sequence assembly errors and impose multi-level challenges for genome and protein databases. *Nucleic Acids Res*. 2019; doi: 10.1093/nar/gkz841.

7. Bredeson JV, Lyons JB, Prochnik SE, Wu GA, Ha CM, Edsinger-Gonzales E, et al.. Sequencing wild and cultivated cassava and related species reveals extensive interspecific hybridization and genetic diversity. *Nat Biotechnol*. Nature Publishing Group; 2016; doi: 10.1038/nbt.3535.

8. Kuon J-E, Qi W, Schläpfer P, Hirsch-Hoffmann M, von Bieberstein PR, Patrignani A, et al.. Haplotype-resolved genomes of geminivirus-resistant and geminivirus-susceptible African cassava cultivars. *BMC Biol*. 2019; doi: 10.1186/s12915-019-0697-6.

9. Hu W, Ji C, Shi H, Liang Z, Ding Z, Ye J, et al.. Allele-defined genome reveals biallelic differentiation during cassava evolution. *Mol Plant*. Elsevier; 2021; doi: 10.1016/j.molp.2021.04.009.

10. Wang W, Feng B, Xiao J, Xia Z, Zhou X, Li P, et al.. Cassava genome from a wild ancestor to cultivated varieties. *Nat Commun*. 2014; doi: 10.1038/ncomms6110.

11. Chen F, Song Y, Li X, Chen J, Mo L, Zhang X, et al.. Genome sequences of horticultural plants: past, present, and future. *Hortic Res*. Nature Publishing Group; 2019; doi: 10.1038/s41438-019-0195-6.

12. Prochnik S, Marri PR, Desany B, Rabinowicz PD, Kodira C, Mohiuddin M, et al.. The Cassava Genome: Current Progress, Future Directions. *Trop Plant Biol*. 2012; doi: 10.1007/s12042-011-9088-z.

13. van Dijk EL, Jaszczyszyn Y, Naquin D, Thermes C. The Third Revolution in Sequencing Technology. *Trends Genet TIG*. 2018; doi: 10.1016/j.tig.2018.05.008.

14. The Vertebrate Genome Project. A reference standard for genome biology. *Nat Biotechnol*. Nature Publishing Group; 2018; doi: 10.1038/nbt.4318.

15. Nurk S, Walenz BP, Rhie A, Vollger MR, Logsdon GA, Grothe R, et al.. HiCanu: accurate assembly of segmental duplications, satellites, and allelic variants from high-fidelity long reads. *Genome Res*. 2020; doi: 10.1101/gr.263566.120.

16. Wenger AM, Peluso P, Rowell WJ, Chang P-C, Hall RJ, Concepcion GT, et al.. Accurate circular consensus long-read sequencing improves variant detection and assembly of a human genome. *Nat Biotechnol*. Nature Publishing Group; 2019; doi: 10.1038/s41587-019-0217-9.

17. Vollger MR, Logsdon GA, Audano PA, Sulovari A, Porubsky D, Peluso P, et al.. Improved assembly and variant detection of a haploid human genome using single-molecule, high-fidelity long reads. *bioRxiv*. 2019; doi: 10.1101/635037.

18. Porubsky D, Ebert P, Audano PA, Vollger MR, Harvey WT, et al. Fully phased human genome assembly without parental data using single-cell strand sequencing and long reads. Nat Biotechnol. 2021 Mar;39(3):302-308. doi: 10.1038/s41587-020-0719-5.

19. Cheng H, Concepcion GT, Feng X, Zhang H, Li H. Haplotype-resolved de novo assembly using phased assembly graphs with hifiasm. *Nat Methods*. 2021; doi: 10.1038/s41592-020-01056-5.

20. Zhou Q, Tang D, Huang W, Yang Z, Zhang Y, Hamilton JP, et al.. Haplotype-resolved genome analyses of a heterozygous diploid potato. *Nat Genet*. Nature Publishing Group; 2020; doi: 10.1038/s41588-020-0699-x.

21. Edger PP, Poorten TJ, VanBuren R, Hardigan MA, Colle M, McKain MR, et al.. Origin and evolution of the octoploid strawberry genome. *Nat Genet*. 2019; doi: 10.1038/s41588-019-0356-4.

22. Rabbi IY, Hamblin MT, Kumar PL, Gedil MA, Ikpan AS, Jannink J-L, et al.. High-resolution mapping of resistance to cassava mosaic geminiviruses in cassava using genotyping-by-sequencing and its implications for breeding. *Virus Res*. 2014; doi: 10.1016/j.virusres.2013.12.028.

23. Chin C-S, Peluso P, Sedlazeck FJ, Nattestad M, Concepcion GT, Clum A, et al.. Phased diploid genome assembly with single-molecule real-time sequencing. *Nat Methods*. 2016; doi: 10.1038/nmeth.4035.

24. Cheng H, Concepcion GT, Feng X, Zhang H, Li H. Haplotype-resolved de novo assembly using phased assembly graphs with hifiasm. Nat Methods. 2021 Feb;18(2):170-175. doi: 10.1038/s41592-020-01056-5.

25. Rhie A, McCarthy SA, Fedrigo O, Damas J, Formenti G, et al.. Towards complete and error-free genome assemblies of all vertebrate species. Nature. 2021 Apr;592(7856):737-746. doi: 10.1038/s41586-021-03451-0.

26. Simpson JT. Exploring genome characteristics and sequence quality without a reference. *Bioinformatics*. Oxford Academic; 2014; doi: 10.1093/bioinformatics/btu023.

27. Iqbal Z, Caccamo M, Turner I, Flicek P, McVean G. De novo assembly and genotyping of variants using colored de Bruijn graphs. *Nat Genet*. Nature Publishing Group; 2012; doi: 10.1038/ng.1028.

28. Rhie A, Walenz BP, Koren S, Phillippy AM. Merqury: reference-free quality, completeness, and phasing assessment for genome assemblies. *Genome Biol*. 2020; doi: 10.1186/s13059-020-02134-9.

29. Chin C-S, Alexander DH, Marks P, Klammer AA, Drake J, Heiner C, et al.. Nonhybrid, finished microbial genome assemblies from long-read SMRT sequencing data. *Nat Methods*. Nature Publishing Group; 2013; doi: 10.1038/nmeth.2474.

30. International Cassava Genetic Map Consortium (ICGMC). High-resolution linkage map and chromosome-scale genome assembly for cassava (Manihot esculenta Crantz) from 10 populations. *G3 Bethesda Md*. 2014; doi: 10.1534/g3.114.015008.

31. Phytozome. Phytozome info: M.esculenta v8.1. https://phytozome-next.jgi.doe.gov/info/Mesculenta_v8_1 Accessed 2022 Feb 14.

32. Wilson MC, Mutka AM, Hummel AW, Berry J, Chauhan RD, Vijayaraghavan A, et al.. Gene expression atlas for the food security crop cassava. *New Phytol*. 2017; doi: 10.1111/nph.14443.

33. Smet RD, Adams KL, Vandepoele K, Montagu MCEV, Maere S, Peer YV de. Convergent gene loss following gene and genome duplications creates single-copy families in flowering

plants. *Proc Natl Acad Sci*. National Academy of Sciences; 2013; doi: 10.1073/pnas.1300127110.

34. Nattestad M, Schatz MC. Assemblytics: a web analytics tool for the detection of variants from an assembly. *Bioinformatics*. Oxford Academic; 2016; doi: 10.1093/bioinformatics/btw369.

35. Marçais G, Delcher AL, Phillippy AM, Coston R, Salzberg SL, Zimin A. MUMmer4: A fast and versatile genome alignment system. *PLOS Comput Biol*. Public Library of Science; 2018; doi: 10.1371/journal.pcbi.1005944.

36. Hosseini M, Pratas D, Morgenstern B, Pinho AJ. Smash++: an alignment-free and memory-efficient tool to find genomic rearrangements. *GigaScience*. Oxford Academic; 2020; doi: 10.1093/gigascience/giaa048.

37. Zielezinski A, Girgis HZ, Bernard G, Leimeister C-A, Tang K, Dencker T, et al.. Benchmarking of alignment-free sequence comparison methods. *Genome Biol*. 2019; doi: 10.1186/s13059-019-1755-7.

38. Cheng S-P, Jia K-H, Liu H, Zhang R-G, Li Z-C, Zhou S-S, et al.. Haplotype-resolved genome assembly and allele-specific gene expression in cultivated ginger. *Hortic Res*. 2021; doi: 10.1038/s41438-021-00599-8.

39. Zhang X, Chen S, Shi L, Gong D, Zhang S, Zhao Q, et al.. Haplotype-resolved genome assembly provides insights into evolutionary history of the tea plant Camellia sinensis. *Nat Genet*. 2021; doi: 10.1038/s41588-021-00895-y.

40. Koren S, Rhie A, Walenz BP, Dilthey AT, Bickhart DM, Kingan SB, et al.. De novo assembly of haplotype-resolved genomes with trio binning. *Nat Biotechnol*. 2018; doi: 10.1038/nbt.4277.

41. Morley SA, Nielsen BL. Plant mitochondrial DNA. *Front Biosci Landmark Ed*. 2017; doi: 10.2741/4531.

42. Kozik A, Rowan BA, Lavelle D, Berke L, Schranz ME, Michelmore RW, et al.. The alternative reality of plant mitochondrial DNA: One ring does not rule them all. *PLOS Genet*. Public Library of Science; 2019; doi: 10.1371/journal.pgen.1008373.

43. Zhou Y, Minio A, Massonnet M, Solares E, Lv Y, Beridze T, et al.. The population genetics of structural variants in grapevine domestication. *Nat Plants*. Nature Publishing Group; 2019; doi: 10.1038/s41477-019-0507-8.

44. Kou Y, Liao Y, Toivainen T, Lv Y, Tian X, Emerson JJ, et al.. Evolutionary genomics of structural variation in Asian rice (Oryza sativa) domestication. *Mol Biol Evol*. 2020; doi: 10.1093/molbev/msaa185.

45. Bayer PE, Golicz AA, Scheben A, Batley J, Edwards D. Plant pan-genomes are the new reference. *Nat Plants*. Nature Publishing Group; 2020; doi: 10.1038/s41477-020-0733-0.

46. Della Coletta R, Qiu Y, Ou S, Hufford MB, Hirsch CN. How the pan-genome is changing crop genomics and improvement. *Genome Biol*. 2021; doi: 10.1186/s13059-020-02224-8.

47. Li J, Yuan D, Wang P, Wang Q, Sun M, Liu Z, et al.. Cotton pan-genome retrieves the lost sequences and genes during domestication and selection. *Genome Biol*. 2021; doi: 10.1186/s13059-021-02351-w.

48. Sun X, Jiao C, Schwaninger H, Chao CT, Ma Y, Duan N, et al.. Phased diploid genome assemblies and pan-genomes provide insights into the genetic history of apple domestication. *Nat Genet*. 2020; doi: 10.1038/s41588-020-00723-9.

49. Li H, Feng X, Chu C. The design and construction of reference pangenome graphs with minigraph. *Genome Biol*. 2020; doi: 10.1186/s13059-020-02168-z.

50. Peterson DG, Tomkins JP, Frisch DA, Wing RA, Paterson AH. Construction of plant bacterial artificial chromosome (BAC) libraries: an illustrated guide. *J Agric Genomics*. National Center for Genome Resources; 5:1–32000;

51. Gonthier L, Bellec A, Blassiau C, Prat E, Helmstetter N, Rambaud C, et al.. Construction and characterization of two BAC libraries representing a deep-coverage of the genome of chicory (Cichorium intybus L., Asteraceae). *BMC Res Notes*. 2010; doi: 10.1186/1756-0500-3-225.

52. Chalhoub B, Belcram H, Caboche M. Efficient cloning of plant genomes into bacterial artificial chromosome (BAC) libraries with larger and more uniform insert size. *Plant Biotechnol J*. 2004; doi: 10.1111/j.1467-7652.2004.00065.x.

53. Wingett S, Ewels P, Furlan-Magaril M, Nagano T, Schoenfelder S, Fraser P, et al.. HiCUP: pipeline for mapping and processing Hi-C data. *F1000Research*. 2015; doi: 10.12688/f1000research.7334.1.

54. Simpson JT, Durbin R. Efficient de novo assembly of large genomes using compressed data structures. *Genome Res*. 2012; doi: 10.1101/gr.126953.111.

55. Pacific Biosciences. IPA HiFi Genome Assembler. https://github.com/PacificBiosciences/pbipa.

56. Gurevich A, Saveliev V, Vyahhi N, Tesler G. QUAST: quality assessment tool for genome assemblies. *Bioinformatics*. Oxford Academic; 2013; doi: 10.1093/bioinformatics/btt086.

57. Earl D, Bradnam K, St John J, Darling A, Lin D, Fass J, et al.. Assemblathon 1: a competitive assessment of de novo short read assembly methods. *Genome Res*. 2011; doi: 10.1101/gr.126599.111.

58. Guan D. Asset. https://github.com/dfguan/asset.

59. Seppey M, Manni M, Zdobnov EM. BUSCO: Assessing Genome Assembly and Annotation Completeness. In: Kollmar M, editor. *Gene Predict Methods Protoc*. New York, NY: Springer;

60. Goodstein DM, Shu S, Howson R, Neupane R, Hayes RD, Fazo J, et al.. Phytozome: a comparative platform for green plant genomics. *Nucleic Acids Res*. 2012; doi: 10.1093/nar/gkr944.

61. Li H. Minimap2: pairwise alignment for nucleotide sequences. *Bioinformatics*. Oxford Academic; 2018; doi: 10.1093/bioinformatics/bty191.

62. Weirather JL, de Cesare M, Wang Y, Piazza P, Sebastiano V, Wang X-J, et al.. Comprehensive comparison of Pacific Biosciences and Oxford Nanopore Technologies and their applications to transcriptome analysis. *F1000Research*. 2017; doi: 10.12688/f1000research.10571.1.

63. Kent WJ. BLAT—The BLAST-Like Alignment Tool. *Genome Res*. 2002; doi: 10.1101/gr.229202.

64. Arima. Arima mapping pipeline. https://github.com/ArimaGenomics/mapping_pipeline.

65. Ghurye J, Rhie A, Walenz BP, Schmitt A, Selvaraj S, Pop M, et al.. Integrating Hi-C links with assembly graphs for chromosome-scale assembly. *PLOS Comput Biol*. Public Library of Science; 2019; doi: 10.1371/journal.pcbi.1007273.

66. Tang H, Zhang X, Miao C, Zhang J, Ming R, Schnable JC, et al.. ALLMAPS: robust scaffold ordering based on multiple maps. *Genome Biol*. 2015; doi: 10.1186/s13059-014-0573-1.

67. Benson G. Tandem repeats finder: a program to analyze DNA sequences. *Nucleic Acids Res*. 1999; doi: 10.1093/nar/27.2.573.

68. Bao Z, Eddy SR. Automated de novo identification of repeat sequence families in sequenced genomes. *Genome Res*. 2002; doi: 10.1101/gr.88502.

69. Wootton JC, Federhen S. Statistics of local complexity in amino acid sequences and sequence databases. *Comput Chem*. 1993; doi: 10.1016/0097-8485(93)85006-X.

70. Smit A, Hubley R, Green P. RepeatMasker Open-4.0. 2013-2015.

71. Gremme G, Steinbiss S, Kurtz S. GenomeTools: A Comprehensive Software Library for Efficient Processing of Structured Genome Annotations. *IEEE/ACM Trans Comput Biol Bioinform*. 2013; doi: 10.1109/TCBB.2013.68.

72. Ou S, Jiang N. LTR_retriever: A Highly Accurate and Sensitive Program for Identification of Long Terminal Repeat Retrotransposons1[OPEN]. *Plant Physiol*. 2018; doi: 10.1104/pp.17.01310.

73. Wheeler T. Large-scale neighbor-joining with NINJA”inAlgorithms in Bio-informatics. *Lect Notes Comput Sci*. Springer; p. 375–89.

74. Katoh K, Standley DM. MAFFT Multiple Sequence Alignment Software Version 7: Improvements in Performance and Usability. *Mol Biol Evol*. 2013; doi: 10.1093/molbev/mst010.

75. Fu L, Niu B, Zhu Z, Wu S, Li W. CD-HIT: accelerated for clustering the next-generation sequencing data. *Bioinformatics*. 2012; doi: 10.1093/bioinformatics/bts565.

76. Flynn JM, Hubley R, Goubert C, Rosen J, Clark AG, Feschotte C, et al.. RepeatModeler2 for automated genomic discovery of transposable element families. *Proc Natl Acad Sci U S A*. 2020; doi: 10.1073/pnas.1921046117.

77. Campbell MS, Law M, Holt C, Stein JC, Moghe GD, Hufnagel DE, et al.. MAKER-P: A Tool Kit for the Rapid Creation, Management, and Quality Control of Plant Genome Annotations. *Plant Physiol*. 2014; doi: 10.1104/pp.113.230144.

78. Shumate A, Salzberg SL. Liftoff: an accurate gene annotation mapping tool. Bioinformatics; 2020 Jun.

79. Wang Y, Tang H, Debarry JD, Tan X, Li J, Wang X, et al.. MCScanX: a toolkit for detection and evolutionary analysis of gene synteny and collinearity. *Nucleic Acids Res*. 2012; doi: 10.1093/nar/gkr1293.

80. Bandi VK. SynVisio: A Multiscale Tool to Explore Genomic Conservation [Thesis]. University of Saskatchewan; 2020 http://hdl.handle.net/10388/12895

81. Hoff KJ, Stanke M. Predicting Genes in Single Genomes with AUGUSTUS. *Curr Protoc Bioinforma*. 2019; doi: 10.1002/cpbi.57.

82. Bray NL, Pimentel H, Melsted P, Pachter L. Near-optimal probabilistic RNA-seq quantification. *Nat Biotechnol*. 2016; doi: 10.1038/nbt.3519.

83. Love MI, Huber W, Anders S. Moderated estimation of fold change and dispersion for RNA-seq data with DESeq2. *Genome Biol*. 2014; doi: 10.1186/s13059-014-0550-8.

84. Conway JR, Lex A, Gehlenborg N. UpSetR: an R package for the visualization of intersecting sets and their properties. *Bioinformatics*. 2017; doi: 10.1093/bioinformatics/btx364.

85. Peng Y, Leung HCM, Yiu SM, Chin FYL. IDBA - A Practical Iterative de Bruijn Graph De Novo Assembler. *Lect Notes Comput Sci*. 2010; doi: 10.1007/978-3-642-12683-3_28.

86. Pacific Biosciences. PacificBiosciences/pbsv. https://github.com/PacificBiosciences/pbsv.

87. Jeffares DC, Jolly C, Hoti M, Speed D, Shaw L, Rallis C, et al.. Transient structural variations have strong effects on quantitative traits and reproductive isolation in fission yeast. *Nat Commun*. Nature Publishing Group; 2017; doi: 10.1038/ncomms14061.

88. Li H. lh3/gfatools. https://github.com/lh3/gfatools.

89. Alexa A. Gene set enrichment analysis with topGO. *undefined*. 2006;

90. Alexa A, Rahnenfuhrer J. topGO: Enrichment Analysis for Gene Ontology. Bioconductor version: Release (3.14);

91. Qi W; Lim Y; Patrignani A; Schläpfer P; Bratus-Neuenschwander A; Grüter S; Chanez C; Rodde N; Prat E; Vautrin S; Fustier M; Pratas D; Schlapbach R; Gruissem W. The haplotype-resolved chromosome pairs and transcriptome data of a heterozygous diploid African cassava cultivar GigaScience Database 2022. http://doi.org/10.5524/102193.

Figure Click here to access/download;Figure;GigaScience-TME204-HiFi-HiC-Figures-Final-submitted.docx

Figure 1. Benchmarking analysis of cassava TME204 assemblies from PacBio CLRs and HiFi reads.

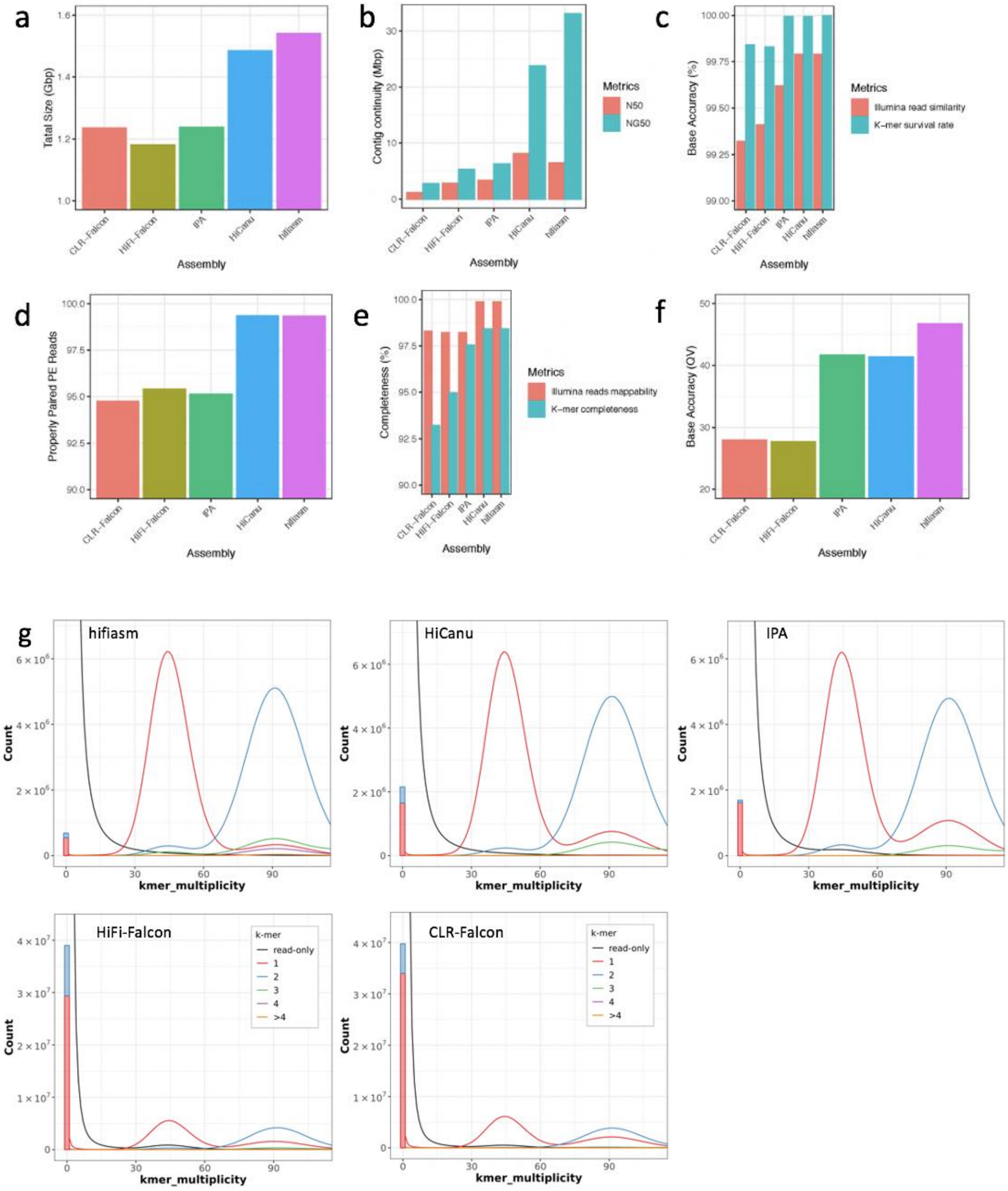

(a) Assembly size of all resolved alleles. (b) Contig continuity measured as N50 and NG50. N50 is the length of the shortest contig in the set of largest contigs that make up 50% of the assembly size as shown in (a). NG50 is the length of the shortest contig in the set of largest contigs that make up 50%

of the haploid genome size of 750 Mbp. (c) Base accuracy of contigs, measured by sequence similarity between contigs and mapped Illumina reads, and as the fraction of k-mers found in both the assembled sequences and the Illumina reads. (d) Structure accuracy of contigs, measured by the percentage of properly paired Illumina PE reads. (e) Assembly completeness, measured by the percentage of mapped Illumina reads and the fraction of reliable Illumina k-mers retained in the contigs. (f) Phred scale quality value (QV) of contigs, calculated using the error probability P with the formula: QV=-10*log(P, 10), where P is the fraction of k-mers found in the contigs but missing in the Illumina reads. (g) Completeness of resolved haplotypes measured by Merqury copy number spectrum plots. Red peaks at 45X k-mer multiplicity represent resolved haplotype alleles, red peaks at 90X k-mer multiplicity collapsed haplotype alleles. K-mer coverage on the x-axis is computed from the Illumina reads. The y-axis is the abundance for k-mers with a given coverage, either in the Illumina reads or in the contigs, and depends on k-mer origin. Black represents k-mers from the Illumina reads. K-mers from contigs were colored by the number of times they are found in the underlying assembly. Black humps found either at 40x (heterozygotes/1-copy k-mers) or 80x (homozygotes/2-copy k-mers) represent reliable Illumina k-mers missing in contigs, which is used as the measure for assembly completeness in (e). The assembly specific k-mers absent from the Illumina reads are plotted as a bar at zero k-mer multiplicity, which is used as the measure for error probability in (f).

Figure 2. Merqury assembly and copy number spectrum plots of cassava TME204 haplotigs.

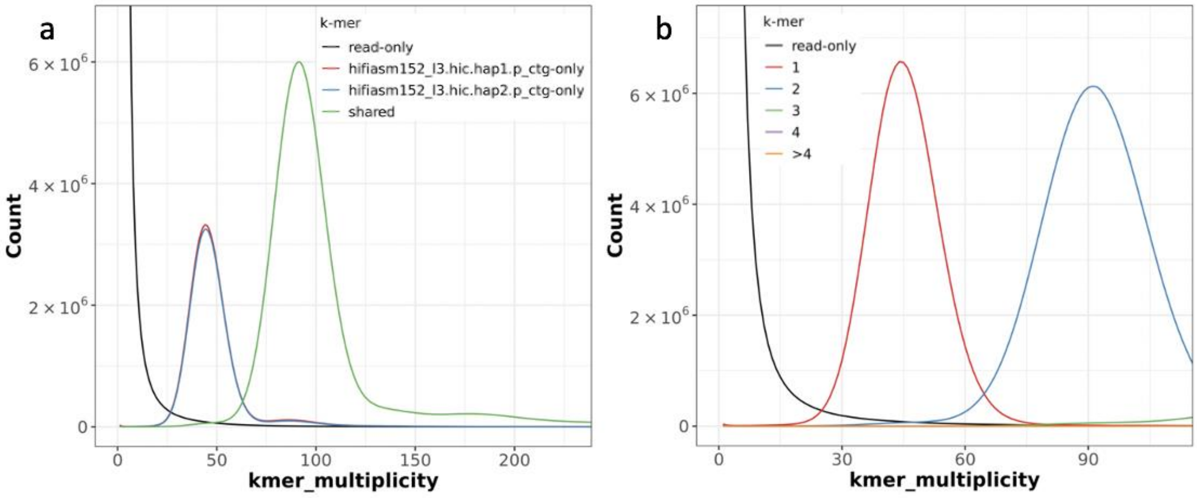

(a) For the Merqury assembly plot, k-mers are colored by their uniqueness in the Illumina PE reads (black), haplotype 1 (red) and haplotype 2 (blue) assemblies. Shared k-mers are shown in green. At the heterozygotes peak (45 x), the second haplotype has only slightly fewer k-mers (blue) compared to the first haplotype (red), indicating the reconstruction of heterozygous variants was almost complete. Red hump and blue shoulder around 90x are haplotype specific k-mers that are actually from homozygotes sequences, green shoulder around 45 x is due to shared k-mers belonging to heterozygotes. These shoulders are all very small, suggesting a very low level of collapsed homozygous regions and artificial duplications. (b) In the copy number spectrum plot, the majority of heterozygous k-mers appear once (red peak at 45x) and the majority of homozygous k-mers twice (blue peak at 90x), confirming that the assembly is close to complete haplotype-resolved and even the homozygous part of the genome is included in both haplotypes. High k-mer completeness is supported by the lack of black humps at 45x or 90x. Low artificial duplication is revealed by the barely detectable humps (green, purple, orange) of duplicated k-mers. The bars at zero k-mer multiplicity are low in both plots, suggesting most k-mers in the assemblies are also present in Illumina reads and therefore the assembled sequences are of high consensus accuracy.

Figure 3. Phasing accuracy of cassava TME204 haplotigs validated by BAC-to-haplotig alignments.

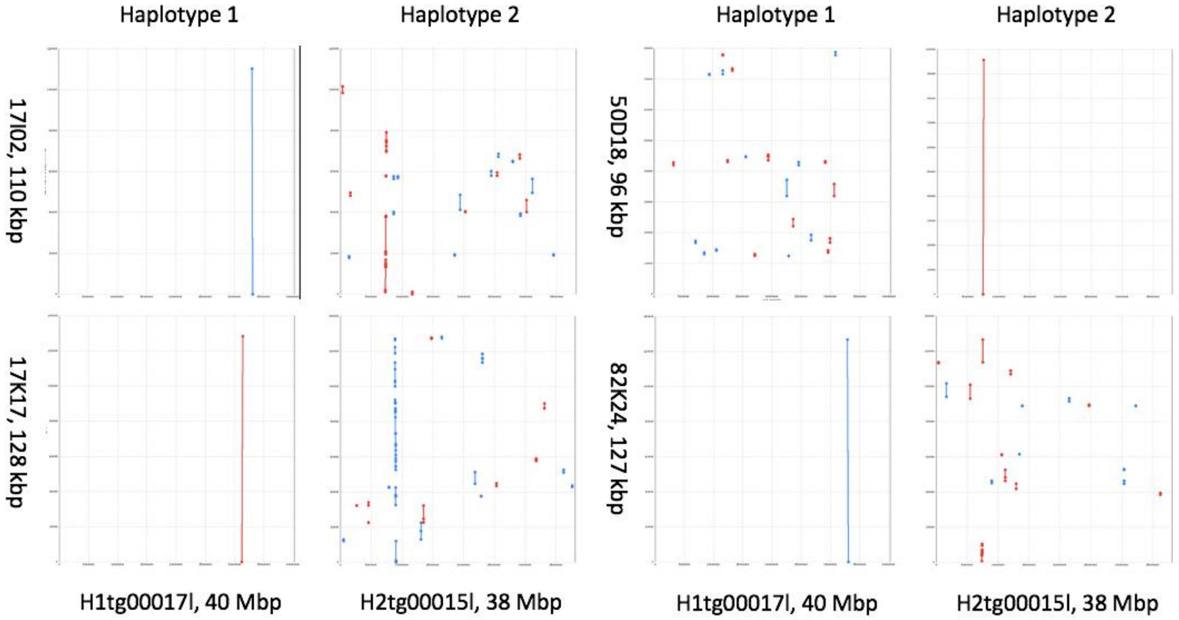

Each dot plot shows the alignment of one BAC (y-axis) with one haplotig (x-axis). Forward alignments are plotted as red lines/dots, reverse alignments in blue. A line represents an undisturbed segment of alignment. When a region is correctly assembled and phased, the corresponding BAC sequence will align continuously (a resolved BAC). Three of the four BACs were resolved in the TME204 H1 assembly, one (50D18) was resolved in the TME204 H2 assembly. For each BAC, the striking differences of BAC-to-haplotig alignments between haplotypes reveal the high level of haplotype differences in these regions.

Figure 4. Reconstruction of pseudochromosomes in the cassava TME204 H1 assembly using the high-density genetic map.

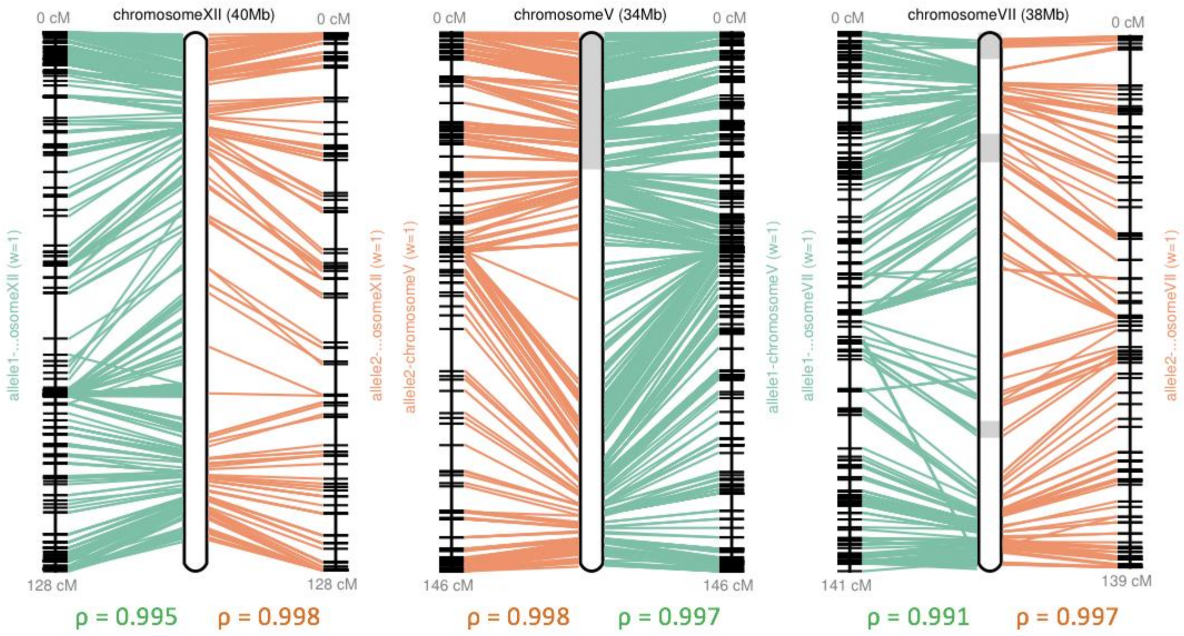

For each pseudochromosome, the panel shows the physical positions on the reconstructed pseudochromosome and the map positions connecting by lines. Adjacent contigs within the reconstructed pseudochromosome are shown as boxes with alternating shades. The ρ-value under each map measures the Pearson correlation coefficient, with values in the range of -1 to 1, and values closer to -1 and 1 indicate near-perfect collinearity. Chromosome XII is composed of a single chromosomal haplotig, the same as for chromosomes IV, VIII, XIV, and XVI. Chromosome V is composed of two contigs, the same as for chromosomes II, III, VI, IX, X, XI, XIII, XV, XVII, XVIII. Chromosome VII is composed of six haplotigs, which is the most fragmented chromosome in the TME204 H1 assembly, followed by chromosome I, which has four haplotigs. Plots for all chromosomes in both H1 and H2 assemblies are provided in Supplementary file 5.

Figure 5. Cassava TME204 genome annotation.

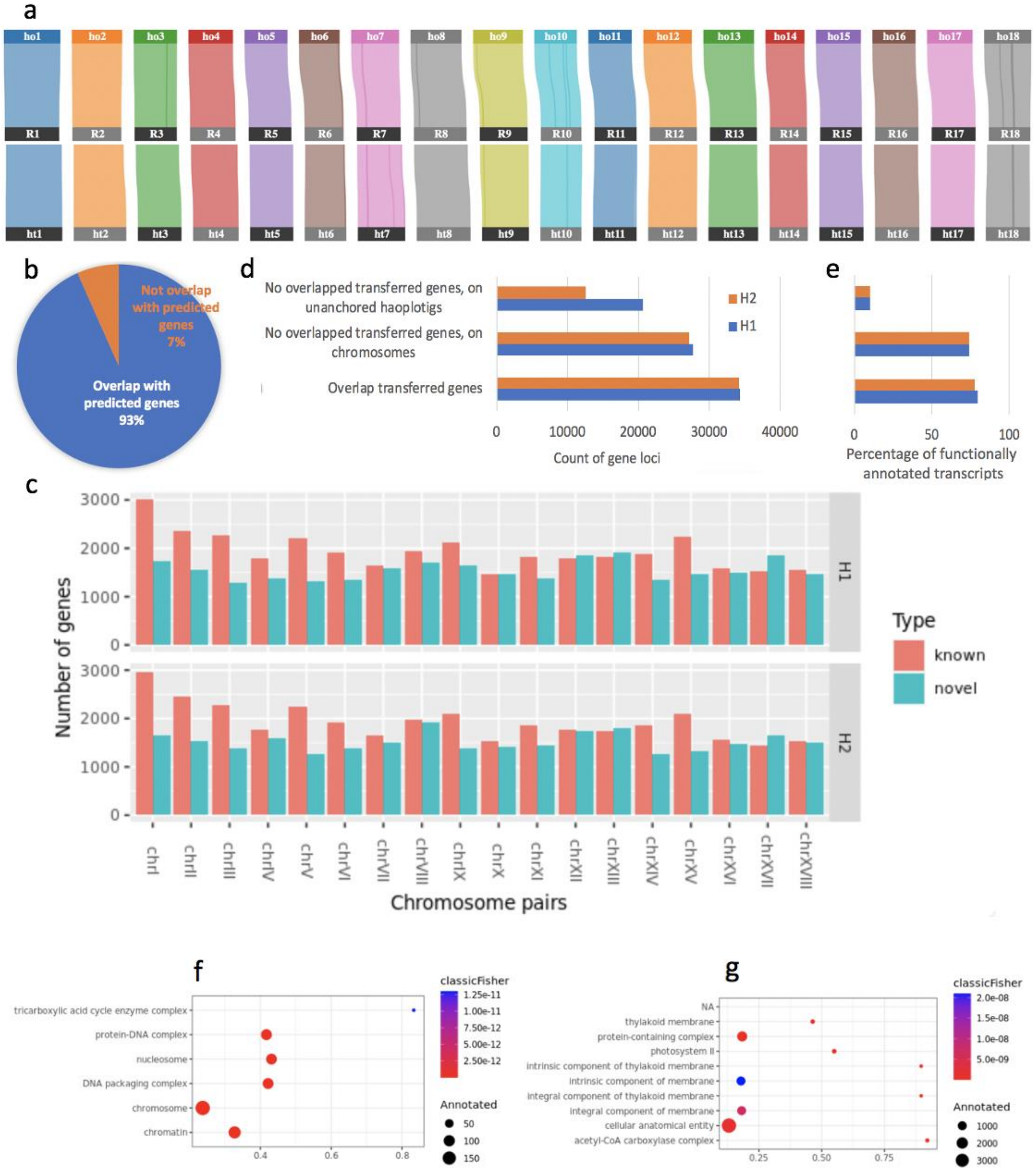

(a) Gene synteny (99%) between AM560 and the TME204 pseudochromosome pairs revealed by orthologous pairs of transferred reference genes. “ho” and “ht” encode “haplotype one” and “haplotype two” of TME204, respectively. “R” encodes “reference”. Color lines highlight the inverted regions. (b) Recall rate (93%) of the transferred reference genes by *ab initio* gene prediction in the TME204 H1 assembly. For the TME204 H2 assembly the recall rate is 94%. (c) Distribution of *ab*

initio predicted gene loci among TME204 chromosome pairs. “known” represents predicted gene loci overlapped with transferred reference gene models. “novel” represents predicted gene loci without any overlapping transferred reference gene models. (d) Amount of novel predicted gene loci on unanchored haplotigs in comparison to that of chromosomal gene loci in TME204 assemblies. In comparison to the H1 assembly, the H2 assembly has less unanchored haplotigs (60 Mbp instead of 100 Mbp, Table 2). As a result, the number of novel predicted gene loci from these sequences is also lower. (e) Fractions of functionally annotated transcripts, which are grouped similarly as predicted gene loci, as described in c and colored in d. Bubble plots of enriched cellular component (CC) terms in novel chromosomal genes (f) and novel genes from unanchored haplotigs (g). The colors of the bubbles are illustrated from blue to red in descending order of –log10 (p-value). The sizes of the bubbles are from small to large in ascending order of total gene counts annotated with the CC terms shown on the y-axis. The x-axis represents the ratio of novel/total gene counts. Results shown are from the TME204 H1 assembly. The enriched CC terms in the TME204 H2 assembly are almost identical (Supplementary figure 7 c and d).

Figure 6. Haplotype resolved analysis of differentially expressed transcripts during cassava TME204 tissue development.

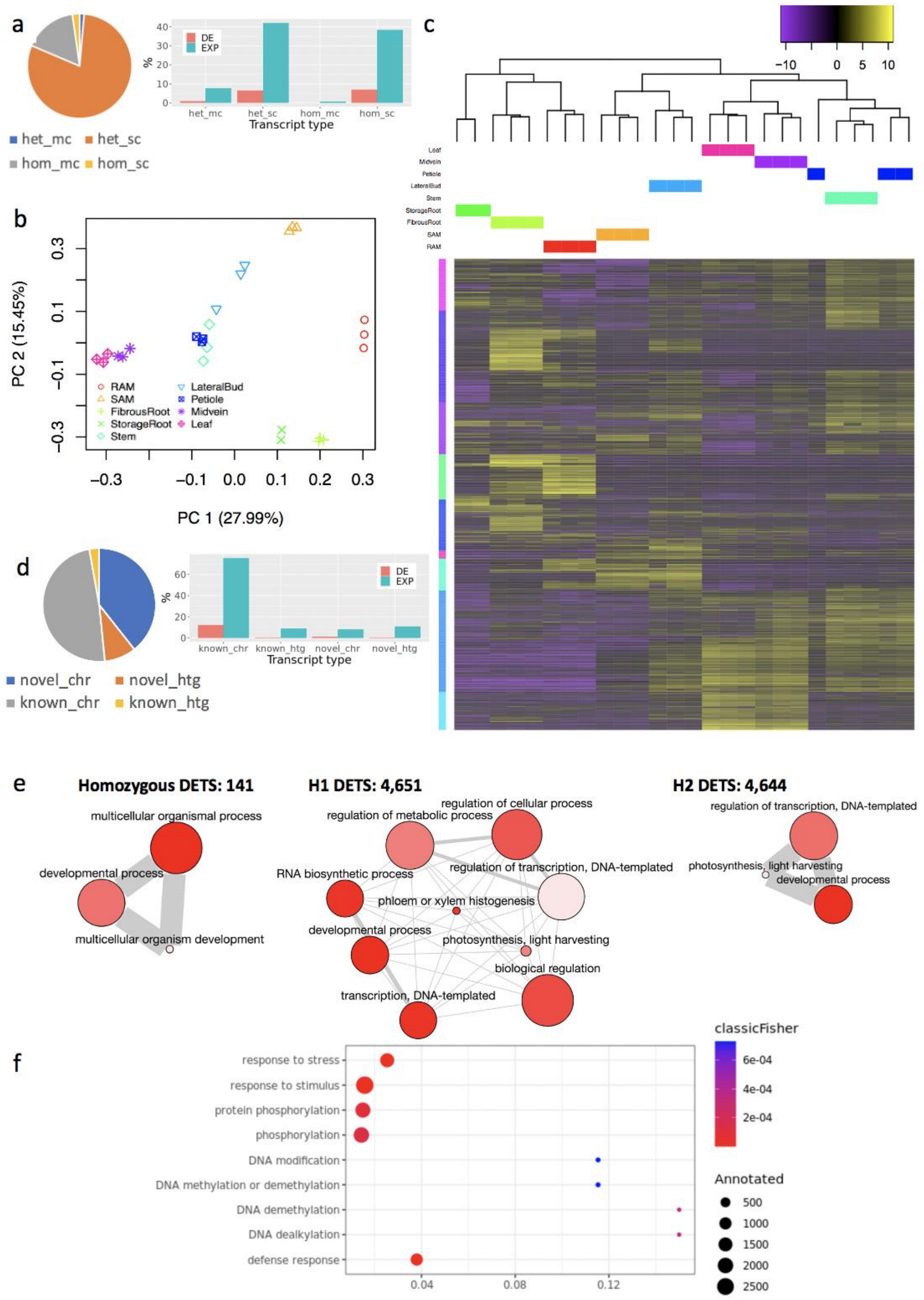

(a) Clustering analysis of transcripts from both TME204 H1 and H2 assemblies. “hom” encodes transcripts with identical sequences between haplotypes. “het” encodes transcripts with different sequences between haplotypes. “mc” represents multi-copy transcripts within one haplotype assembly. “sc” represents single-copy transcripts in one haplotype assembly. Identical transcripts (“hom” and “mc”) are collapsed and present only once in the haplotype resolved reference transcriptome. Multi-copy (“het_mc” and “hom_mc”) transcripts are less highly expressed (“EXP”) and differentially regulated (“DE”, adjusted p-value < 0.00001 and a fold change of log2|FC| >2) during TME204 tissue development. (b) Principal component analysis (PCA) of samples based on transcript expression levels. (c) Sample and transcript clustering analysis of the 9,436 differentially expressed transcripts (DETs) across the nine tissues (adjusted p-value < 0.00001 and log2|FC| >2). The color scale represents row-centered expression levels. (d) Transcripts from chromosomal, novel predicted gene loci are also less highly expressed (”EXP”) and differentially regulated (“DE”, adjusted p-value < 0.00001 and a log2|FC| >2) in different TME204 tissues. (e) ReviGo graphs of enriched biological process (BP) terms in DETs with different haplotype origins: homozygous DETs common to both H1 and H2, DETs from the H1 assembly and DETs to from H2 assembly. Each GO term is a node. Related GO terms are connected by edges between the nodes. Node color indicates the Fisher exact test p-value. The lighter the color, the lower the p-value. Node size corresponds to the frequency of the GO term in the whole UniProt database. (f) Bubble plot of enriched BP terms in the 663 chromosomal, novel predicted DETs.

Figure 7. Allele-specific expression in cassava TME204.

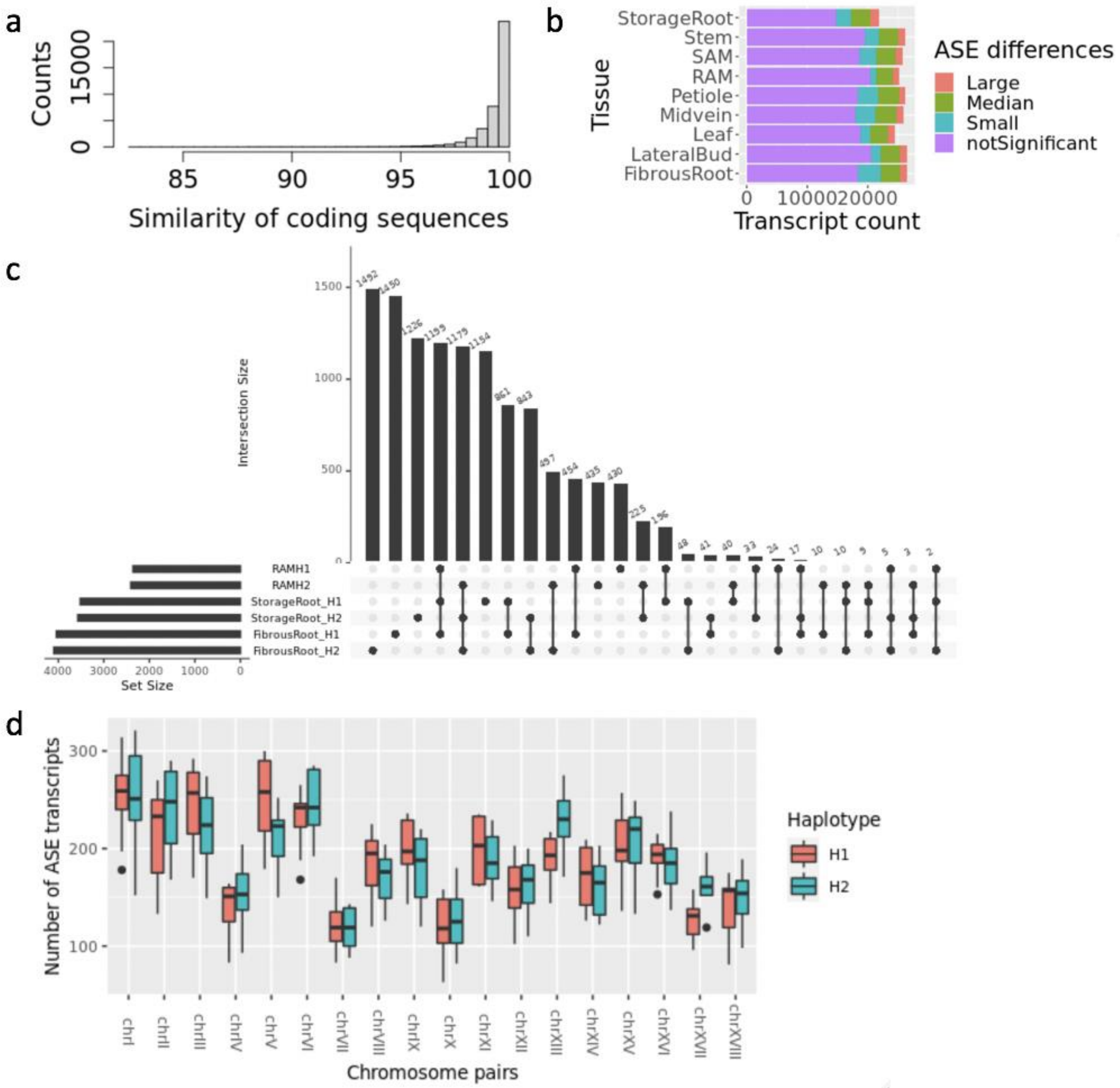

(a) Coding sequence similarity of the 39,028 bi-allelic transcript pairs in TME204. (b) Number of the bi-allelic transcripts expressed in nine cassava tissues. The expressed transcripts were further classified into four categories: “notSignificant”: no significant allelic expression differences (p-value ≥ 0.05); “Small”: significant allelic expression differences with a log2|FC| < 1 (p-value < 0.05); “Median”: significant allelic expression differences with a log2|FC|≥ 1 and log2|FC| < 3, p-value < 0.05); and “Large”: significant allelic expression differences with a log2|FC| ≥ 3 (p-value < 0.05). Storage root has fewer expressed transcripts than all other tissues, which is partially due to fewer biological replicates (two instead of three). (c) Upset plot showing allele-specific

expression (ASE) among the TME204 subterranean tissues. Transcripts with ASE biased towards H1 or H2 alleles in fibrous root, storage root and RAM are shown as sets at the bottom of the graph, sorted by the number of ASE transcripts in each tissue and haplotype (i.e., Set size). Transcripts overlapping between tissues and haplotypes are connected as dots with vertical lines (i.e., transcript intersection categories). The number of transcripts within each intersection category is plotted above as a vertical black bar, labeled with the transcript count. (d) Distribution of transcripts with ASE differences between pseudochromosome pairs in the TME204 diploid genome. Transcripts with ASE biased towards H1 alleles were counted for H1 pseudochromosomes, transcripts with ASE biased towards H2 alleles for H2 pseudochromosomes. Box plots summarize the average across all nine tissues.

Figure 8. Identification of structural variants in cassava TME204 and AM560 genomes by HiFi reads.

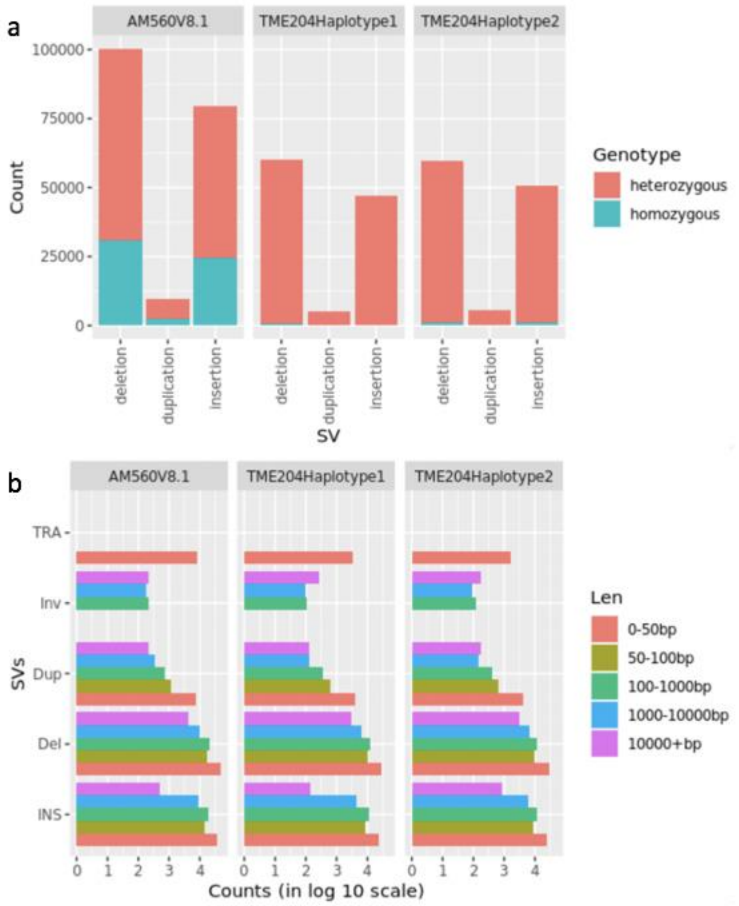

(a). Classification and counts of SVs by genotypes. (b) Classification and counts of SVs by variant types and length. INS: insertions; Del: deletions; Dup: duplications; INV: inversions; TRA: breakpoints of complex variants with unknow sizes, such as translocations etc.

Figure 9. Chromosome XII maps of TME204 and AM560 show extensive genomic rearrangements between the chromosome pairs.

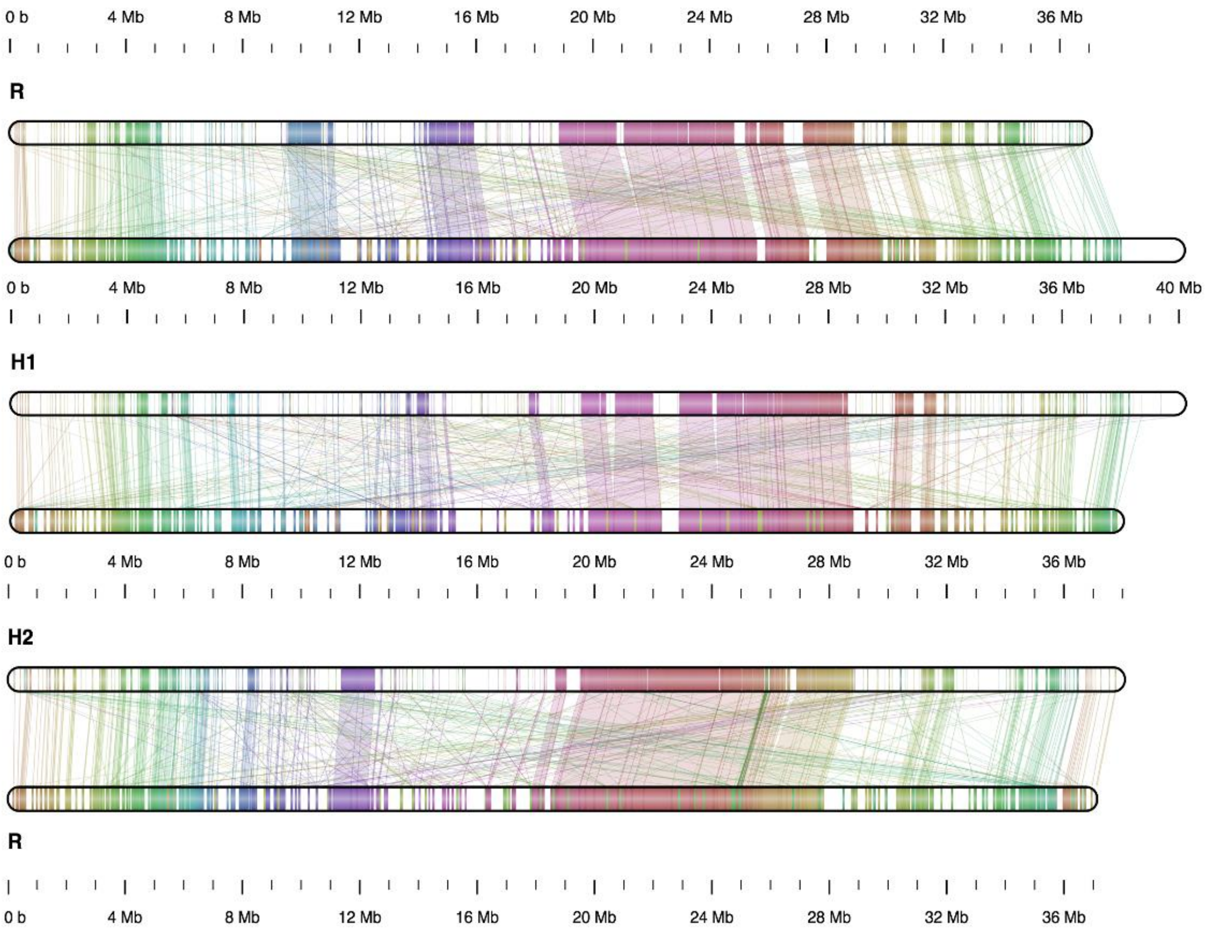

"R" indicates the pseudochromosome from the reference AM560 v8.0 assembly, "H1" pseudochromosome from the TME204 haplotype 1 assembly, "H2" pseudochromosome from the TME204 haplotype 2 assembly. Shared regions between chromosome pairs are shown as color segments and connected by color lines between chromosomes. Shared regions with similar sequence information content were detected by Smash++ with parameters adjusted for highly repetitive genomes (Methods). White segments represent regions that are degenerated between a chromosome pair. Such accumulation of degenerated genomic sequences was observed between all pseudochromosome pairs (Supplementary file 6), both within the TME204 diploid genome, and between each TME204 haplotype and the AM560 haploid genome.

211 **Figure 10. Properties of cassava pan-genomes.**

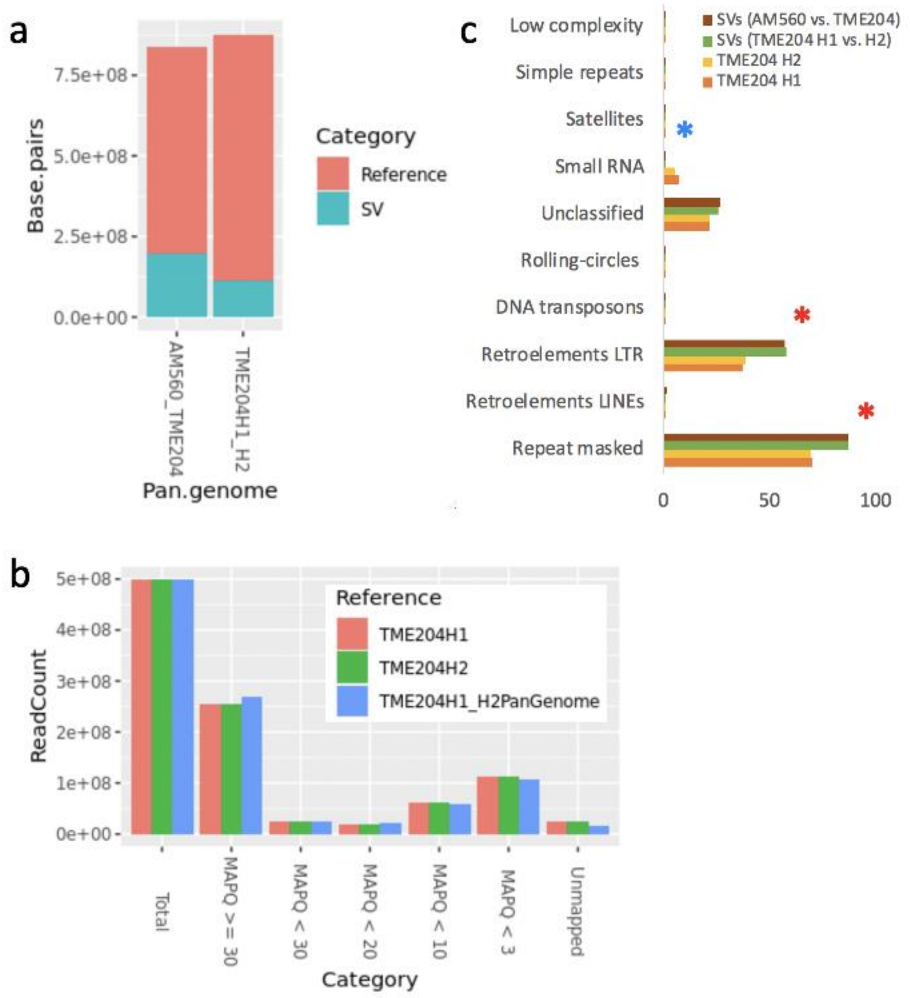

212

213 (a) Cassava pan-genomes across different assemblies and large SVs (100 bp – 100 kbp) detected by
214 pan-genome graphs. The pan-genome size decreased when AM560 was included because there
215 were less one-to-one orthogonal regions between AM560, TME204 H1, and H2. (b) The pan-genome
216 of TME204 H1 and H2 improved mapping rate and mapping quality of Illumina PE reads collected
217 from the same DNA sample. (c) SVs in cassava pan-genomes are enriched with repeats (Chi square
218 test p-value < 0.05), especially LTR elements (p-value < 0 .05), and are deprived of small RNAs (p-
219 value < 0.005).

Click here to access/download
Supplementary Material
GigaScience-TME204-HiFi-HiC-Supp-Final-submitted.docx

Click here to access/download;Personal Cover;GigaScience-TME204-HiFI-HiC-ReviewerReports-Final-Submitted.docx

Answers to reviewer reports:

Reviewer #1: All my concerns have been solved. Thus, I recommend to accept this manuscript.

Answer: Thank you for the positive feedback. The reviewer's comments were helpful for improving the manuscript.

However, before final publication, I suggest the authors to carefully check the typos throughout the whole manuscript. Here I just show you a few examples:

Answer: Thank you for finding small mistakes. Spelling and grammar in all documents have been checked in Microsoft Words again and by a native English speaker.

1) Line 128 and 131, "a fold change (FC) > |4|" is not right. Should be log2|FC| >2? Please check.
Similar errors were found in Lines 153-155.

Answer: The reviewer is correct. All have been corrected.

2) Line 217-218, 'p-vale' should be 'p-value'

Answer: Corrected.

3) Fig.6a, in the right sub-figure, DE might mean "differentially expressed", what does EXP mean? expression?

Answer: Yes. "DE" represents "differentially expressed". "EXP" represents "expressed". The explanations have been added into the figure legends of Fig 6a and Fig 6d.

4) Fig.6c, which color (yellow or purple) represents high and low expression level, respectively?

Answer: The color scale of the heatmap has been added into Fig 6c.

Reviewer #2: The revised manuscript is greatly improved; the authors addressed all of my previous concerns.

Answer: Thank you for the positive feedback. The reviewer's comments were helpful for improving the manuscript.

Abstract Line 3-4: The lack of genetic gain in cassava is due to many factors, not solely the lack of the ability to generate a reference genome due to its heterozygosity. Rephrase.

Answer: The sentence “Genetic gain by molecular breeding is limited because cassava has a highly heterozygous, repetitive and difficult to assemble genome.” has been rephrased to the following: “Genetic gain by molecular breeding has been limited, partially because cassava is a highly heterozygous crop with a repetitive and difficult to assemble genome.”

I think it would be better to use "variable" or "diverged" to refer to ASE rather than inconsistent which implies an experimental problem.

Answer: The terminology of “consistent ASE bias” and “inconsistent ASE bias” have been established and used in multiple publications (Muzzey et al, Crowley et al, Shao et al; see references below). As defined by Shao et al., “genes showing ASE (ASEGs) in various ways, which can be classified into two patterns: consistent ASEGs such that the ASE was biased toward one parental allele in all tissues/conditions, and inconsistent ASEGs such that ASE was found in some but not all tissues/conditions, including direction-shifting ASEGs in which the ASE was biased toward one parental allele in some tissues/conditions while toward the other parental allele in other tissues/conditions.” We therefore follow the established terminology of previous publications and use “inconsistent” to described ASE biases in our manuscript.

Muzzey D, et al. Extensive and coordinated control of allele-specific expression by both transcription and translation in Candida albicans. Genome Res. 2014 Jun;24(6):963-73. doi: 10.1101/gr.166322.113.

Crowley JJ, et al. Analyses of allele-specific gene expression in highly divergent mouse crosses identifies pervasive allelic imbalance [published correction appears in Nat Genet. 2015 Jun;47(6):690]. Nat Genet. 2015;47(4):353-360. doi:10.1038/ng.3222.

Shao L, et al. Patterns of genome-wide allele-specific expression in hybrid rice and the implications on the genetic basis of heterosis. Proc Natl Acad Sci U S A. 2019 Mar 19;116(12):5653-5658. doi: 10.1073/pnas.1820513116.

Line 80: Explain how being clonally propagated can increase the number of effective alleles. What is an effective allele? F1 hybrids are heterozygous so how is clonal propagation unique?

Answer: In the manuscript we wrote that “Clonally propagation can increase the effective number of alleles and heterozygosity in the population.” We explain this statement below supported by relevant literature references. We are not further elaborating the statement in our current manuscript because this will be further explained and substantiated by results in another manuscript that is currently in preparation.

“Clonal propagation leads to accumulation of somatic mutations [1,2]. As mutations accumulate over time, the growing plant can become a genetic mosaic [3], with cell lineages differing in somatic mutations. A cell lineage with a mutation conferring an advantage in terms of cell proliferation can replace other lineages. Such diplontic selection at the cellular level can purge deleterious mutations, allowing favorable new alleles to accumulate in the

plant [4,5]. Gametes can bear the somatic mutations of the cell lineage from which they are derived. When different somatic mutations from different cell lineages are inherited, the clonal descendants of the plant can be genetically heterogeneous. Because continued diplontic selection leads to accumulation of favorable mutations, the gametes are not a random set but are also biased towards favorable mutations. As a source of genetic variation, it has been shown that inherited somatic mutations could be quantitatively comparable to mutations occurring during meiosis [6]."

1. Whitham TG, Slobodchikoff CN. Evolution by individuals, plant-herbivore interactions, and mosaics of genetic variability: The adaptive significance of somatic mutations in plants. *Oecologia*. 1981; doi: 10.1007/BF00347587.

2. McKey D, Elias M, Pujol B, Duputié A. The evolutionary ecology of clonally propagated domesticated plants. *New Phytol*. 2010; doi: 10.1111/j.1469-8137.2010.03210.x.

3. Gill DE, Chao L, Perkins SL, Wolf JB. Genetic Mosaicism in Plants and Clonal Animals. *Annu Rev Ecol Syst*. 1995; doi: 10.1146/annurev.es.26.110195.002231.

4. Otto SP, Hastings IM. Mutation and selection within the individual. *Genetica*. 102–103:507–241998;

5. Pineda-Krch M, Lehtila K. Cell lineage dynamics in stratified shoot apical meristems. *J Theor Biol*. 2002; doi: 10.1006/jtbi.2002.3139.

6. Orive ME. Somatic mutations in organisms with complex life histories. *Theor Popul Biol*. 2001; doi: 10.1006/tpbi.2001.1515.

Clarify which version of hifiasm was used in Supp table 1 vs Table 2. The metrics for the alternate assembly in Supp Table 1 seem poor.

Answer: The version numbers have been added in Supp table 1 and the header of Table 2. The alternate assembly in Supp Table 1 was without Hi-C phasing. The alternate assembly in Table 2 was phased with Hi-C.

Line 298 and others: It should be non-mitochondrial not none-mitochondrial

Answer: Corrected.

If the pseudochromsomes were assigned randomly to H1 and H2, how can you explain differential GO enrichment in the ASE? What would happen to the enrichment if the pseudochromsomes were reshuffled among the haplotypes?

Answer: We have now explained this in the discussion and added the new Supplementary figure 10: "Between allelic chromosome pairs, the number of ASE transcripts biased towards each haplotype was similar across the genome (Figure 7 d). Finding ASE transcripts will thus still hold true after reshuffling of pseudochromosomes between haplotypes. The number of DETs between allelic chromosome pairs was also similar (Supplementary figure 10 a).

Functional enrichment analysis of DETs per chromosome identified chromosomes with uniquely enriched BP terms (Supplementary figure 10 a and b). The existence of such chromosomes suggests that haplotype-specific enriched functions in DETs will hold true no matter how the chromosomes are shuffled between haplotypes, although the specific set of DETs and actual enriched GO terms per haploid genome will change."

Supplementary figure 10. GO enrichment analysis of DETs per chromosome.

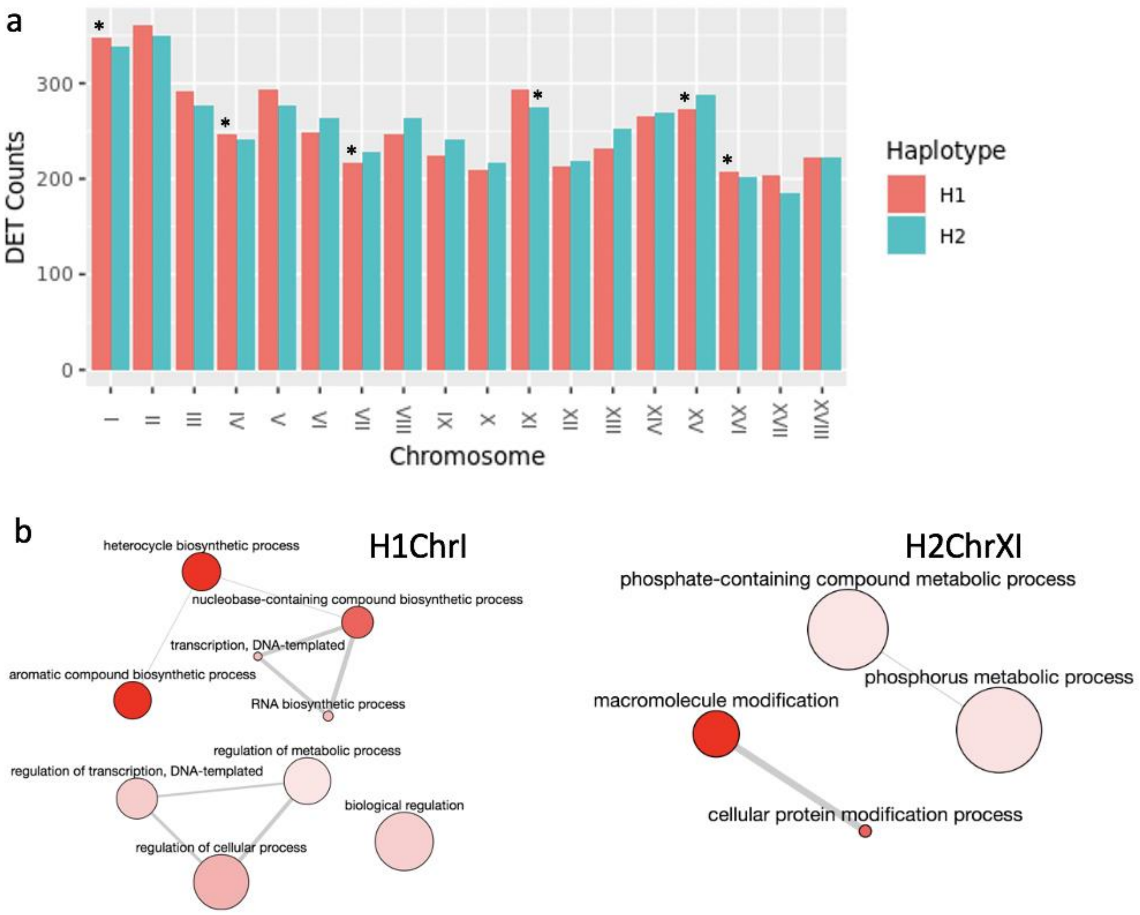

(a) Distribution of DETs between pseudochromosome pairs in the TME204 diploid genome. Chromosomes where DETs were enriched of unique GO BP terms were highlighted with "*". (b) ReviGo graphs of enriched BP terms unique for DETs from different chromosomes. H1 Chromosome I and H2 Chromosome XI were plotted because their DETs identified most abundantly enriched BP terms. Each GO term is a node. Related GO terms are connected by edges between the nodes. Node color indicates the Fisher exact test p-value. The lighter the color, the lower the p-value. Node size corresponds to the frequency of the GO term in the entire UniProt database.
