# Supplementary material for: The haplotype-resolved chromosome pairs of a heterozygous diploid African cassava cultivar reveal novel pan-genome and allele-specific transcriptome features
Source: Gigascience. 2022 Mar 24;11:giac028. doi: 10.1093/gigascience/giac028 (PMC8952263; doi:10.1093/gigascience/giac028)
Supplement: giac028_Supplemental_File [file giac028_supplemental_file.docx]

**Supplementary material supporting the manuscript titled “The haplotype-resolved chromosome pairs of a heterozygous diploid African cassava cultivar reveal novel pan-genome and allele-specific transcriptome features”**

**Supplementary methods**

**Illumina library preparation**

The DNA sample (100 ng) was sonicated with the ME220 Focused-ultrasonicator (PN: 500506, Covaris) using settings specific to the fragment size of 350 bp. The fragmented DNA sample was size- selected using AMpure beads (Beckman Coulter), end-repaired and adenylated. TruSeq adapters containing Unique Dual Indices (UDI) (GACACCATGT and GCACGGTACC) for multiplexing were ligated to the size-selected DNA sample. Fragments containing TruSeq adapters on both ends were selectively enriched by PCR. The quality and quantity of the enriched library were validated using Tapestation (Agilent Technologies). The product is a DNA fragment population with an average fragment size of 500 bp. The library was adjusted to 10nM using a Tris-Cl 10 mM, pH8.5 with 0.1% Tween 20 buffer.

**PacBio CLR and HiFi library preparation**

The DNA samples were mechanically sheared to an average size distribution of 30 Kbp (CLR) and 20 Kbp (HiFi) using a Megaruptor Device (Diagenode). A Femto Pulse gDNA analysis assay (Agilent Technologies) was used to assess the fragment size distribution. Sheared DNA samples were DNA damage-repaired and end-repaired using polishing enzymes. PacBio sequencing adapters were ligated to the DNA template. For the CLR library, a Blue Pippin device (Sage Science) was used to size select DNA fragments > 25 Kbp. For the HiFi library, a Sage Elf device (Sage Science) was used to enrich DNA fragments > 15 Kbp. The size selected DNA libraries were quality-checked and quantified using a Femto Pulse gDNA analysis assay and a Qubit Fluorometer, respectively. The CLR SMRT bell template-polymerase complex was created using the Sequel binding kit 3.0 (Pacific Biosciences). The HiFi SMRT bell template-polymerase complex was created using the Sequel II Binding Kit 2.0 and Internal Control 1.0 (Pacific Biosciences).

**Arima Hi-C library preparation**

Flash-frozen leaves were first crosslinked, followed by Hi-C library generation using the High Coverage Arima Hi-C kit (PN: A410110). Illumina-compatible sequencing libraries were prepared by first shearing purified proximally-ligated DNA and then size-selecting DNA fragments using SPRI beads (Beckman Coulter). The size-selected fragments containing ligation junctions were enriched using Enrichment Beads provided in the High Coverage Arima Hi-C kit and converted into Illumina-compatible sequencing libraries using the Swift Accel-NGS 2S Plus DNA Library Kit (PN: 21096). After adapter ligation, DNA library was PCR-amplified and purified using SPRI beads. The purified DNA library was quality-controlled using qPCR (Roche) and Bioanalyzer (Agilent Technologies).

**PacBio Iso-Seq library preparation**

RNA samples (300 ng) were reverse-transcribed with oligo-dT primer in combination with the 5' template-switching oligonucleotide (TSO). Synthesized first-strand cDNAs from different tissues were multiplexed in the cDNA amplification reaction with barcoded forward and reverse cDNA PCR primers annealing to the sequences of the 5' TSO and 3' oligo-dT primer. Barcoded and amplified cDNAs were purified using ProNex beads (Promega), following the workflow targeting transcripts around 2 kb in length. Purified ds cDNAs were used for PacBio template preparation with the SMRTbell Express Template Prep Kit 2.0 (PN: 100-938-900) (Pacific Biosciences). Afterwards the Iso-Seq SMRT bell template-polymerase complex was prepared using Sequel II Binding Kit 2.1 (Pacific Biosciences) and PacBio sequencing primer v4.

**Screening of the BAC library**

Radiolabeled probes ([α-^33^P]dCTP) were designed to screen the genomic BAC library. DNA fragments were PCR-amplified from TME204 genomic DNA using specific primers (Supplementary table 9).

High-density colony filters were prepared using a robotic workstation QPix2 XT (Molecular Devices, San José, CA, USA). BAC clones were spotted in duplicate using a 7 × 7 pattern onto 22 × 22 cm Membranes Hybond-XL filters (GE-Healthcare, Chicago, IL, USA). The whole BAC library was represented on one filter, containing 55 296 BAC Clones. After incubation at 37°C for 17 h, DNA fixation was processed as follows:

1. Denaturation on Whatman paper soaked with a solution of 0.5 m NaOH and 1.5 m NaCl for 4 min at room temperature and for 10 min at 100°C.

2. Neutralization on a Whatman paper soaked with 1 m Tris–HCl pH 7.4 and 1.5 m NaCl for 10 min, incubation in a solution of 0.25 mg ml−1 proteinase K (Sigma-Aldrich, St. Louis, MO, USA) for 45 min at 37°C and baking for 45 min at 80°C.

3. UV fixation on a Biolink 254 nm crosslinker (Thermo Fisher Scientific, Waltham, MA, USA) with an energy of 120 000 μJ.

Probe radiolabeling and filter hybridization were performed as described in (1). Hybridized filters were imaged with an Amersham™ Typhoon™Biomolecular Imager (GE Healthcare), and analyses were performed using HDFR software (Incogen, Williamsburg, NY, USA). Positive BAC clones detected by hybridization were validated individually by quantitative PCR (qPCR) amplification using the primer pairs used for probe synthesis and TME204 genomic DNA as control. qPCR amplified products were visualized using agarose gel electrophoresis and sequenced to confirm the specific amplification of targeted genes. BAC-end sequencing was used to compare and discriminate identified BAC clones. The insert size of the BAC clones was assessed using the FastNot I restriction enzyme and analyzed by pulsed field gel electrophoresis.

**BAC clone sequencing**

Individual BAC clone DNA were extracted using Nucleobond Xtra midi kit (Macherey-Nagel, Düren, Nordrhein-Westfalen). 2µg of each sample were used for the construction of a multiplexed SMRTbell® library by the INRAE-CNRGV. We followed the PacBio recommendations for Multiplexed Microbial Libraries preparation (PN 101-696-100) with some adjustments by using the SMRTbell Express Prep kit v2.0 (Pacific Biosciences, Menlo Park, CA, USA). The first enzymatic steps consist of removing single-stranded overhangs, repairing any DNA damage and polishing ends of the double stranded fragments and tailing with an A-overhang. Ligation with specific barcoded hairpin T-overhang adapters to both ends of the targeted double-stranded DNA (dsDNA) molecule was performed to create a closed, single-stranded circular DNA. A nuclease treatment was performed on each individual sample by using SMRTbell Enzyme Clean-up kit (Pacific Biosciences, Menlo Park, CA, USA). A size-selection with Blue-Pippin system (Sage Science, Beverly, MA, USA) to remove fragments less than 15Kb was done on pooled sample previously purified with 0.45X AMPure PB beads (Pacific Biosciences, Menlo Park, CA, USA). The size and concentration of the final library were assessed using the FemtoPulse system and the Qubit Fluorometer and Qubit dsDNA HS reagents Assay kit (Thermo Fisher Scientific, Waltham, MA, USA), respectively.

Sequencing primer v2 and Sequel DNA Polymerase 2.0 were annealed and bound, respectively to the SMRTbell library. The library was loaded on one SMRTcell at an on-plate concentration of 90pM using a diffusion loading. Sequencing was performed on the Sequel II system with a run movie time of 30 hours with 120 min pre-extension step and Software v9.0 (PacBio) by Gentyane Genomic Platform (INRAE-Clermont-Ferrand, France).

**BAC clone assembly**

The PacBio raw reads were corrected using SMRTLink_v9.0.0 with 8 passes, then demultiplexed. Residual *E. coli* reads were identified using BLAST+ 2.10.0 and removed using Seqfilter. The HiFi reads were filtered by identification of the vector sequences using cross_match and removed by custom Perl scripts. HiFi reads smaller than 15kb were filtered using Seqfilter, and then subsampled with SeqKit to obtain an estimated average assembly depth of 50x. Assembly of the reads was performed with hifiasm-0.12. To validate the result, the length of the obtained contig were checked and BAC ends sequences were mapped with the extremities on the assembly using BLAST+ 2.10.0. HiFi Reads were remapped to the assembly and depth was obtained with samtools-1.8.

**Benchmarking of consensus accuracy and assembly completeness**

In the mapping-based method, TME204 Illumina PE reads were mapped to all genome drafts using BWA mem (v0.7.17) (2). Statistics of read mapping were collected using samtools (v 1.10) (3) and Qualimap (v2.2.1) (4). Sequence differences between mapped reads and assembled sequences, and fractions of mapped Illumina PE reads were used to measure consensus accuracy and assembly completeness. The alignment free k-mer based analysis was performed using Merqury (v1.1) (5). Meryl (v1.7) was used to identify all k-mers with k=20 present in TME204 Illumina PE reads. The k-mer size of 20 was selected based on a haploid genome size of 750 MB and a diploid genome size of 1.5 Gbp. K-mers in each assembly were evaluated for their presence in the Illumina k-mer spectrum. A k-mer missing in the Illumina set is counted as a base-level ‘error’. The fraction of such ‘erroneous’ k-mers was used to calculate a phred scale consensus accuracy quality value (QV) A QV score of 40 means 1 in 10,000 k-mers was specific to the assembled sequences and missing from the Illumina reads. Assembly completeness was measured by k-mer completeness, which is the fraction of reliable Illumina k-mers retained in the assembly.

***Ab initio* gene prediction using AUGUSTUS with experimental evidence**

TME204 RNA-seq reads from all nine tissues (6) (Supplementary table 8) were aligned to the repeat masked TME204 H1 and H2 assemblies using STAR (v2.7.8a) (7). Uniquely aligned reads were converted to hints of gene structures using bam2hints and bam2wig in AUGUSTUS (v3.4.0) (8). TME204 Iso-Seq reads were aligned to the assemblies using minimap2 (v2.21) (9). The alignments were converted to hints using emtrey (v1.1) (https://github.com/rvolden/emtrey). Protein and transcript sequences of AM560 v8.1 (<https://phytozome-next.jgi.doe.gov/info/Mesculenta_v8_1>) were converted to hints using utility scripts from BRAKER (v2.1.5) (10). Protein sequence alignments were produced using “startAlign.pl” with GenomeThreader as the aligner, then converted to hints using “align2hints.pl”. Transcript sequence alignments were generated using blat (11), filtered using pslCDnaFilter (minId=0.9, bestOverlap, localNearBest=0.005) and converted to hints using “blat2hints.pl”. Training of TME204-specific gene prediction parameters was performed using the H2 assembly and RNA-seq hints. Gene models were first predicted using RNA-seq hints (extrinsicCfgFile=extrinsic.M.RM.E.W.cfg) and the Arabidopsis parameter set. Genes with 100% RNA-seq read support and with distinct amino acid sequences (<80%) were used as the trusted gene file, which was checked and confirmed to be free of erroneous gene structures. A set of 400 from these 18,585 trusted genes was randomly selected as the test gene set, the remaining genes were used to train the TME204 specific gene prediction parameters. The newly trained parameters were used to analyze the test gene set for measuring sensitivity and specificity of gene prediction. TME204-specific meta-parameters were then optimized using the same training gene set with a maximum of five optimization rounds, with an 8-fold cross validation for each new combination of parameters. For training of TME204-specific UTR prediction parameters, the trusted gene set was generated using the utility script “autoAug.pl” by aligning the AM560 v8.1 transcripts to the TME204 H2 assembly. Similarly, 400 from these 58,016 trusted genes were randomly selected as the test gene set, the remaining were used to train the TME204-specific UTR prediction parameters, with three optimization rounds. Accuracy of the trained parameter set was compared against the accuracy of AUGUSTUS Arabidopisis parameter set, where prediction sensitivity and specificity were improved at all levels (nucleotide, exon, gene, UTR exon, UTR base) (Supplementary figure 11). The resulting TME204-specific parameter set, in combination with all alignment hints (TME204 RNA-seq data and Iso-Seq transcripts, AM560 v8.1 protein and transcript sequences) were used to predict protein coding genes in TME204 H1 and H2 assemblies using AUGUSTUS with intrinsic evidence (--extrinsicCfgFile=extrinsic.M.RM.E.W.P.PB.cfg, --alternatives-from-evidence=true).

**Supplementary Results**

**Benchmarking cassava TME204 assemblies from PacBio CLR and HiFi reads**

In the case of a heterozygous, diploid genome such as cassava, diploid-aware assemblers (Falcon, hifiasm and IPA) produce a primary genome assembly, which is a set of primary contigs representing pseudo-haplotypes (the longest continuous stretches of assembled sequences), and an alternate assembly consisting of a set of unphased associated contigs (Falcon) or phased (IPA, hifiasm) haplotigs (i.e., continuous sequences of the same haplotype) (12,13). For a genome with very high sequence divergence between haplotypes, haplotype allelic contigs can be incorrectly placed in the primary assembly, resulting in heterotype duplications that inflate the assembly size. The assembler hifiasm (13) also identifies such contigs and places them into the alternate assembly. Phased haplotigs and purged contigs represent resolved alternative alleles (Supplementary table 1). In contrast to this strategy, HiCanu (14) produces a single set of contigs representing all resolved alleles (Supplementary table 2). To achieve an unbiased comparison of the assembly continuity, accuracy, and completeness of all four assemblers, we first combined primary and alternate sequence assemblies into one contig set of mixed-haplotypes (Figure 1, Supplementary table 3).

The CLR-Falcon contig N50 of TME204 was already 10-fold longer compared to cassava TME3 and 60444 CLR-Falcon contigs (15), although the three genomes have similar levels of repetitiveness and heterozygosity (Supplementary figure 1). This could be due to longer sequencing read length, more accurate sequencing chemistry and base calling algorithms, higher coverage (Table 1), and the improved version of the Falcon assembler.

Because assembled genome sizes varied based on the assembly software (Figure 1 a), N50 values cannot be used for comparisons between assemblers due to their dependency on the assembly size. Consequently, we used NG50 (16) to compare assembly continuity among the HiFi assemblers, since it was normalized using the same haploid genome size of 750 Mbp instead of the varied total assembled sizes.

The consensus accuracy measured using the aligned Illumina PE reads was lower than the measurement using Merqury (5) k-mer analysis, while completeness measured using mapped Illumina PE reads was higher. The sequence differences between the mapped Illumina PE reads and the assembled consensus sequences could originate from sequencing errors in the Illumina reads and/or mis-alignments introduced by the alignment software tool. The read alignment process is also more tolerant to sequencing errors than the k-mer analysis, where erroneous k-mers were identified and excluded. Consequently, hereafter we only used k-mer analysis to measure the accuracy and completeness of the genome sequence assemblies.

**Supplementary Figures**

**Supplementary figure 1.** **K-mer analysis reveal that genomes of cassava cultivars are diploid and highly heterozygous.**


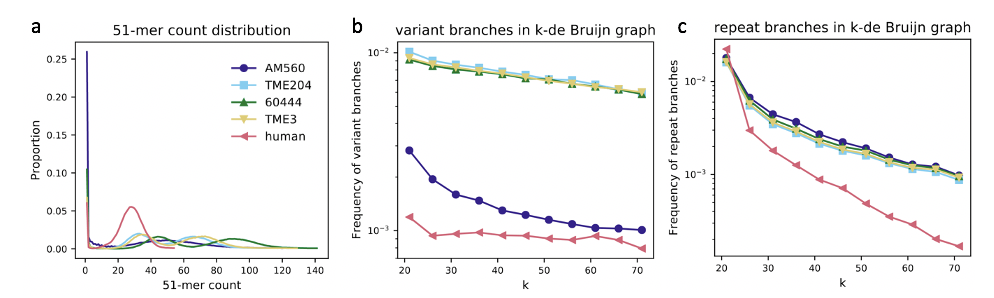


(a) Histogram of k-mer (length=51) coverage, which is the number of times a k-mer is observed in the Illumina reads (x-axis). The relative abundance of k-mers with a given coverage is plotted on the y-axis. For the African cassava TME204, TME3 and 60444 genomes, the k-mer coverage histograms are bi-modal, and the heterozygous peaks (at 35x for TME204/TME3, 45x for 60444) is as high as the homozygous peaks (at 70x for TME204/TME3 and 90x for 60444, respectively), indicating highly heterozygous genomes. For the inbred South-American cassava AM560 and human reference genomes, the histogram is single modal and dominated by the homozygous sequence peaks, indicating homozygous or nearly homozygous genomes. (b) Genome heterozygosity measured as the rate of variant branches (y-axis) in a de Bruijn graph as a function of k-mer length (x-axis). Approximately 1 in 100 vertices in the de Bruijn graphs of TME204, TME3 and 60444 has a variant-induced branch, corresponding to 1 SNP per 100 bp. The level of heterozygosity is 10 times higher than that in the human reference genome. The cassava reference genome was reconstructed from the inbred cassava cultivar AM560, in which the level of heterozygosity is similar to the level in the human reference genome. (c) Genome repetitiveness measured as the rate of repeat branches (y-axis) in a de Bruijn graph as a function of k-mer length (x-axis). The repetitiveness of all four cassava genomes is higher than that of the human genome when measured using k-mers ranging from 25 to 71 bp. The analysis was performed using the preqc (17) module within sga (18).

**Supplementary figure 2. Structural and phasing accuracy of the TME204 phased chromosome XII.**

a


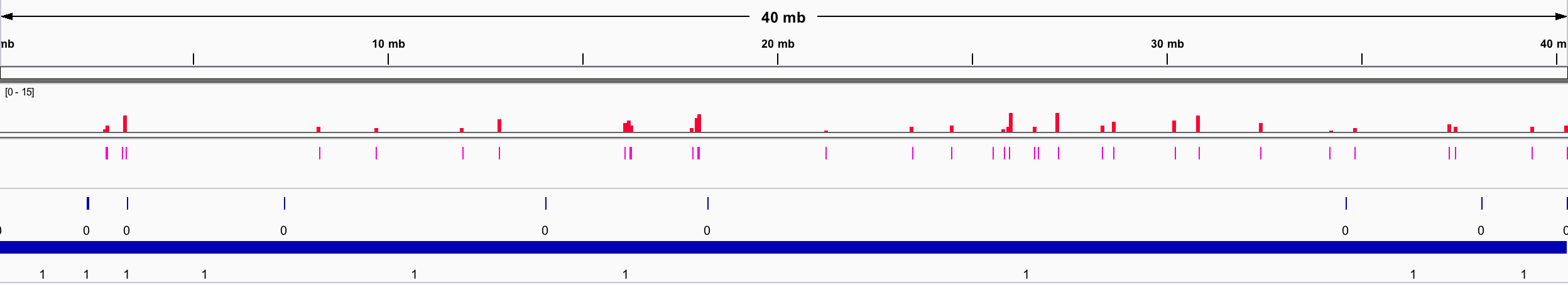


b


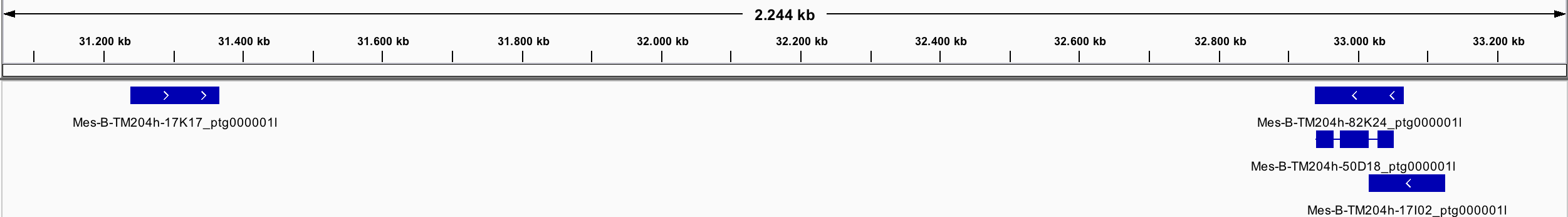


c


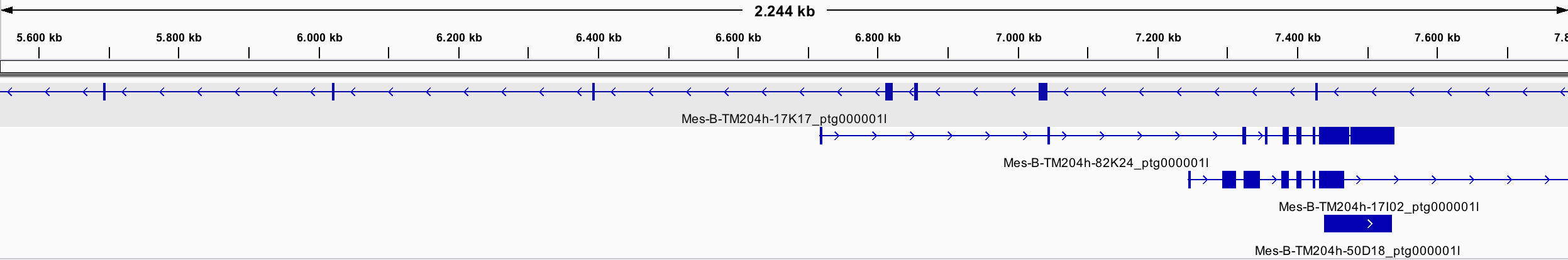


Each phased chromosome was assembled as one haplotig without sequencing gaps. (a) Structural accuracy of the phase 1 chromosome XII (minus-strand, h1tg00017l) measured by k-mers and CLRs. Potentially false duplications are highlighted by locations of k-mers present more than twice in the contig (as magenta bars) and their copy numbers (as red bars). Reliable blocks of assembled sequences with sufficient (≥10) PacBio CLR read support are shown as blue thick lines, labelled with 1 below. Potentially misassembled regions, identified by the lack of PacBio CLR read coverage (< 10), are highlighted as blue bars, labelled with 0 underneath. (b) Phasing accuracy of phase 1 chromosome XII (minus-strand, h1tg00017l) measured using BACs. (c) Phasing accuracy of phase 2 chromosome XII (plus-strand, h2tg00015l) measured using BACs. Three out of four sequenced BACs align continuously in full length with phase 1 chromosome XII. The fourth BAC (50D18) that cannot be aligned continuously with phase 1 aligns in full-length continuously with phase 2 chromosome XII.

**Supplementary figure 3. Distribution of uniquely and perfectly aligned genetic markers among TME204 haplotype 1 (a) and haplotype 2 (b) haplotigs**.

# The presence of phased chromosomes and chromosome pairs can be visually identified at the contig level without sequencing gaps. Most of the 18 chromosome pairs consist of only a few haplotigs. Genetic distance (cM) on the y-axis is derived from the genetic map. Physical distance (Mbp) on the x-axis is derived from the uniquely aligned positions in the TME204 haplotigs. Each dot is a genetic marker. Different colors represent different haplotigs.

#
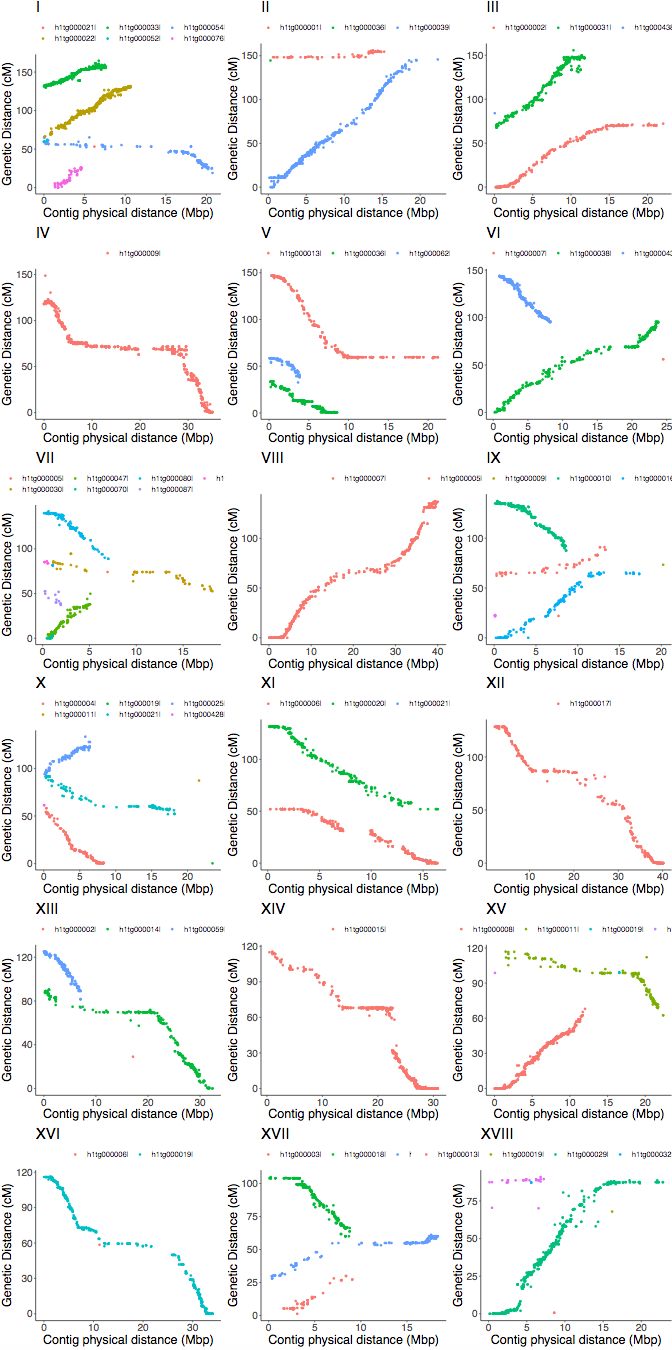


# b

#
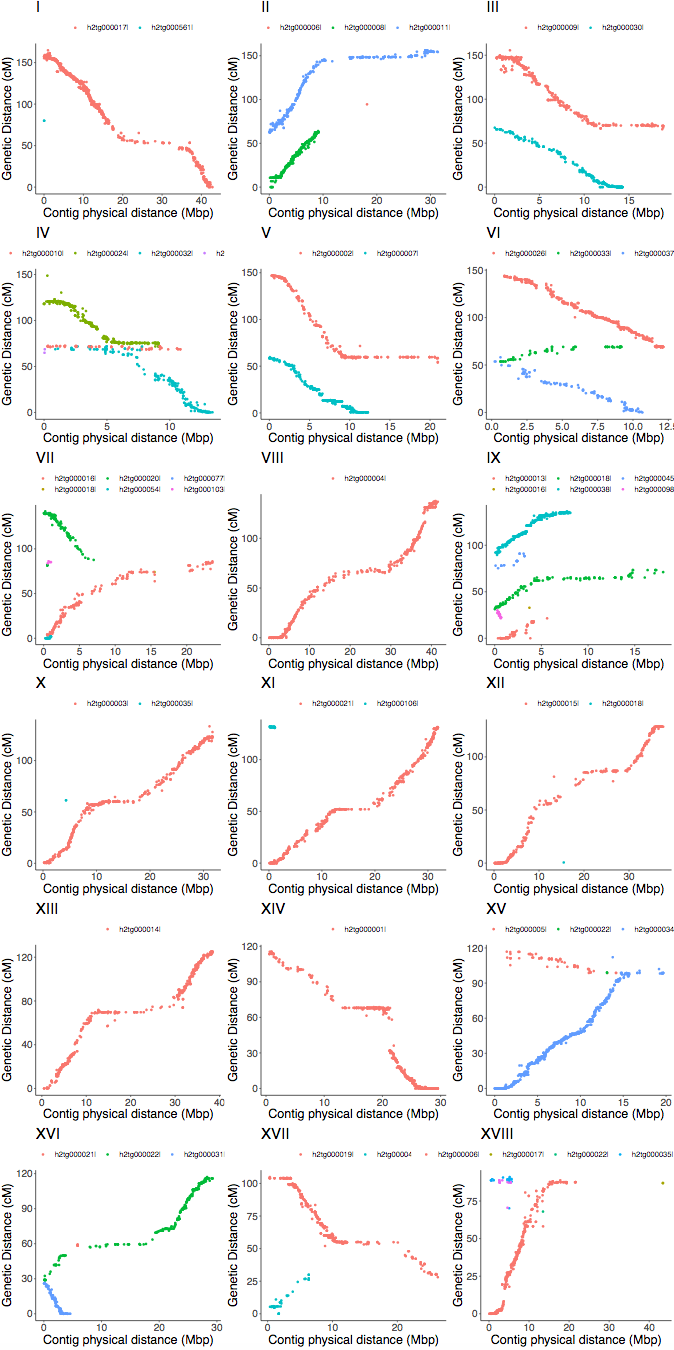


**Supplementary figure 4. Haplotigs of the mitochondrial genome and chromosomal distribution of numt’s in the TME204 diploid genome.**


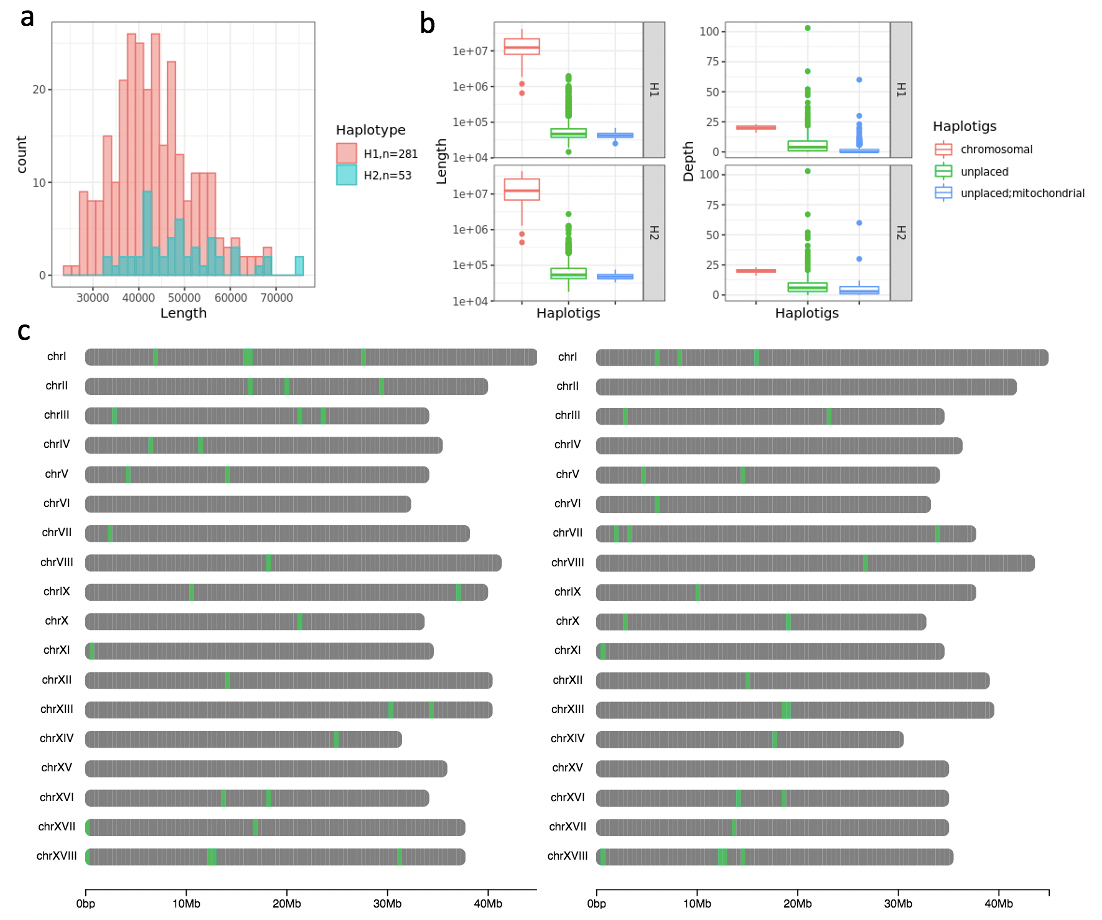


(a) Total counts and length distributions of mitochondrial haplotigs in TME204 H1 and H2 assemblies. (b) Length and depth of coverage for chromosome anchored haplotigs, unanchored mitochondrial haplotigs, and the other unanchored haplotigs. (c) Chromosomal distribution of numt’s in TME204 H1 and H2 assemblies.

**Supplementary figure 5. Repeat landscape of cassava TME204 diploid genome.**


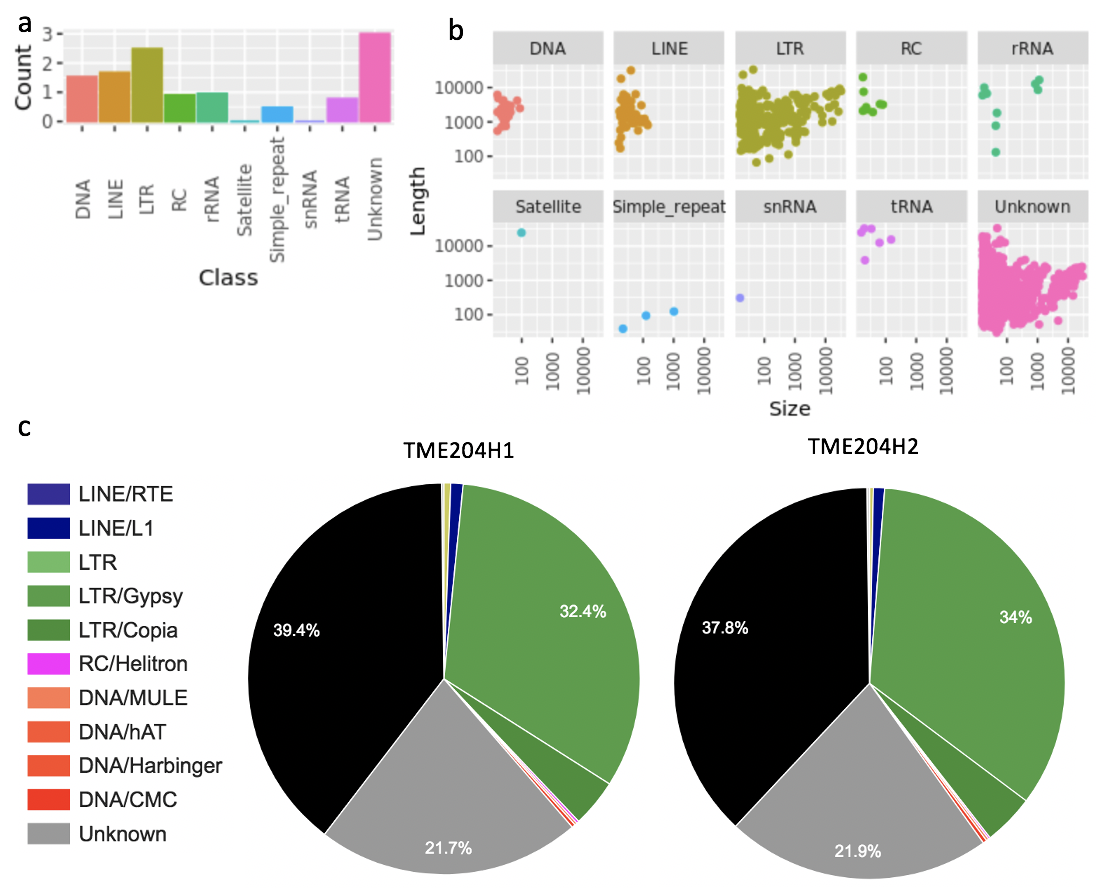


(a) Number of *de novo* predicted repeat families per repeat class. The Y-axis shows the number in log10 scale. (b) Length and copy number of *de novo* predicted repeat families, grouped by repeat classes. (c) Repeat landscape of cassava TME204 H1 and H2 assembly. The black slice represents the fraction of genomics regions not masked, which include none-repetitive sequences, small RNAs and low complexity sequences.

**Supplementary figure 6. BUSCO scores of *ab initio* predicted proteomes in comparison to the underlying genomes.**

**
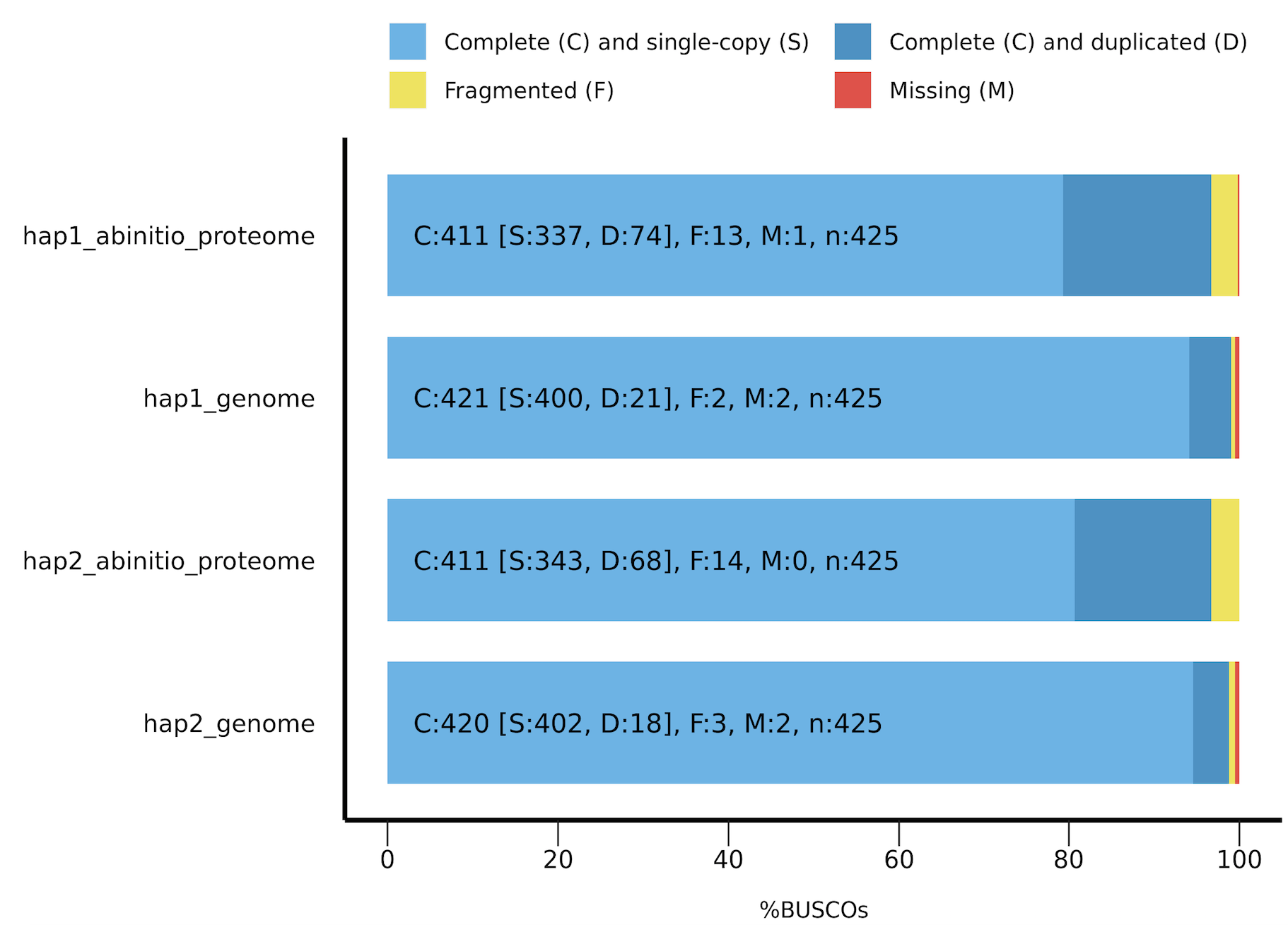
**

**Supplementary figure 7. Enriched gene ontology (GO) terms in novel predicted genes.**


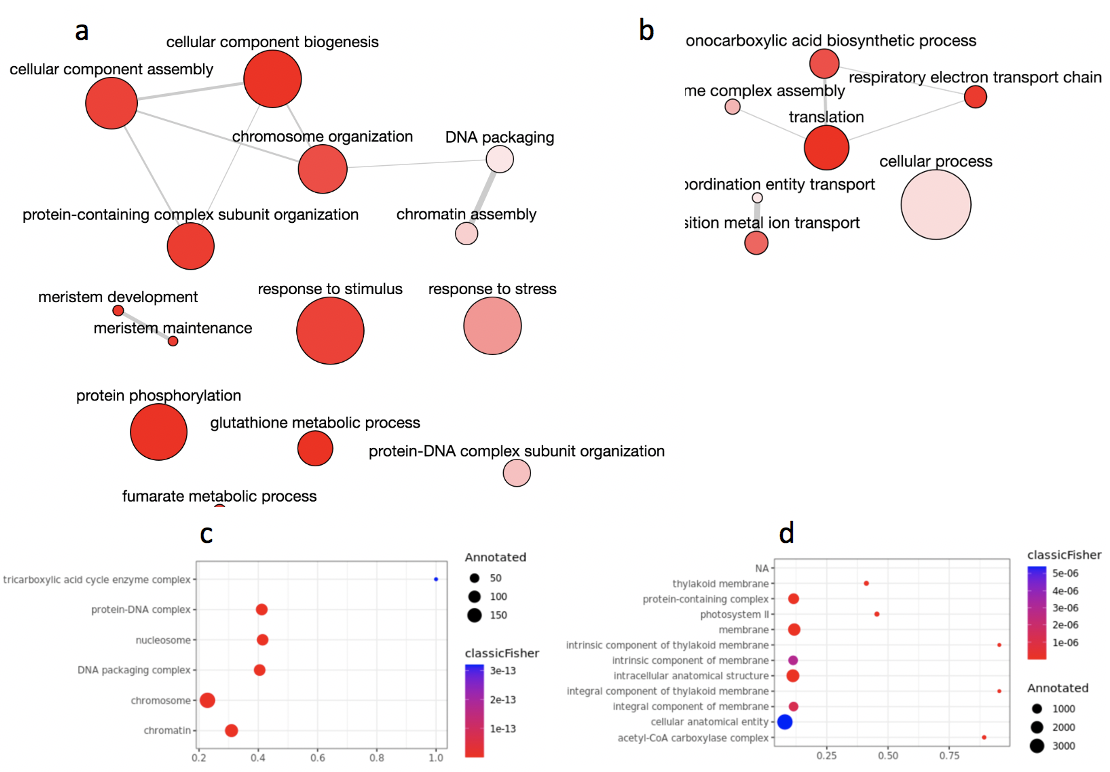


ReviGo (19) graphs of enriched biological process (BP) terms in novel predicted genes on (a) chromosomes and (b) unanchored haplotigs in the TME204 H1 assembly. Each GO term is a node. Related GO terms are connected by edges between the nodes. Bubble color indicates the Fisher exact test p-value. The lighter the color, the lower the p-value. Bubble size indicates the frequency of the GO term in the whole UniProt database. Bubble plots of enriched cellular component (CC) terms in novel chromosomal genes (c) and novel genes from unanchored haplotigs (d) in the TME204 H2 assembly, which are almost identical to those in the TME204 H1 assembly (Figure 5 f and g).

**Supplementary figure 8. Allele-specific expression during TME204 tissue development (6).**


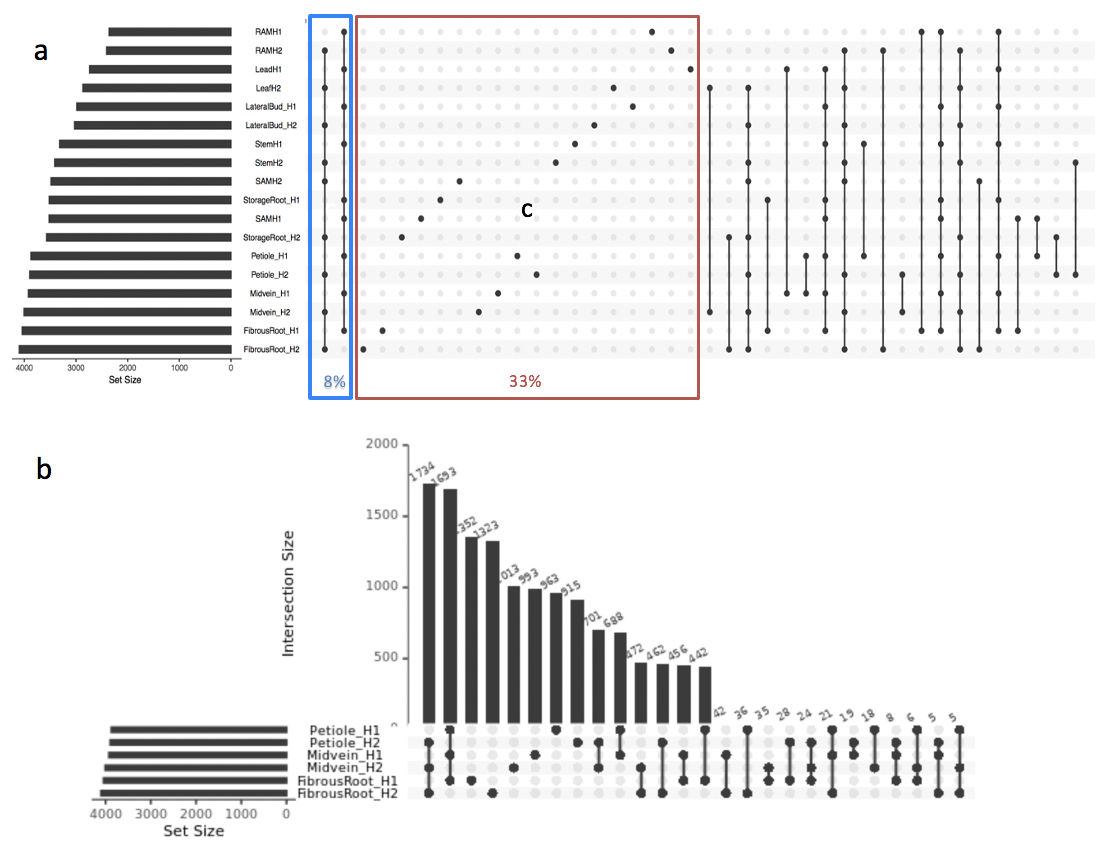


(a) Upset (20) plot showing ASE among all nine TME204 tissues. Only the top 40 intersections are shown. The blue rectangle highlights the fraction of transcripts with consistent ASE differences across all nine tissues. The red rectangle shows the fraction of transcripts where the ASE difference is inconsistent and tissue-specific. (b) Upset plot showing ASE among fibrous root, midvein and petiole, the three tissues with most abundant transcripts showing ASE differences.

**Supplementary figure 9. Classifications and size distributions of small and large indels identified by Assemblytics (21) analysis of reliable contig alignments between cassava genomes.**


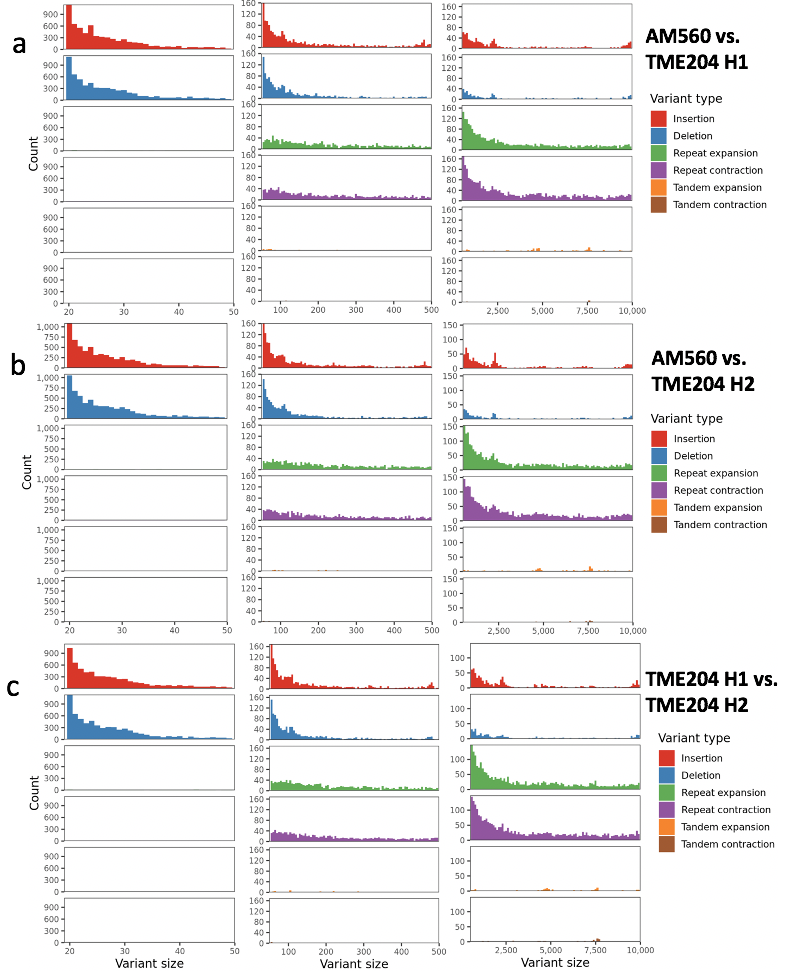


(a) Indels between AM560 contigs and TME204 H1 haplotigs. (b) Indels between AM560 contigs and TME204 H2 haplotigs. (b) Indels between TME204 H1 and H2 haplotigs.

**Supplementary figure 10.** GO enrichment analysis of DETs per chromosome.


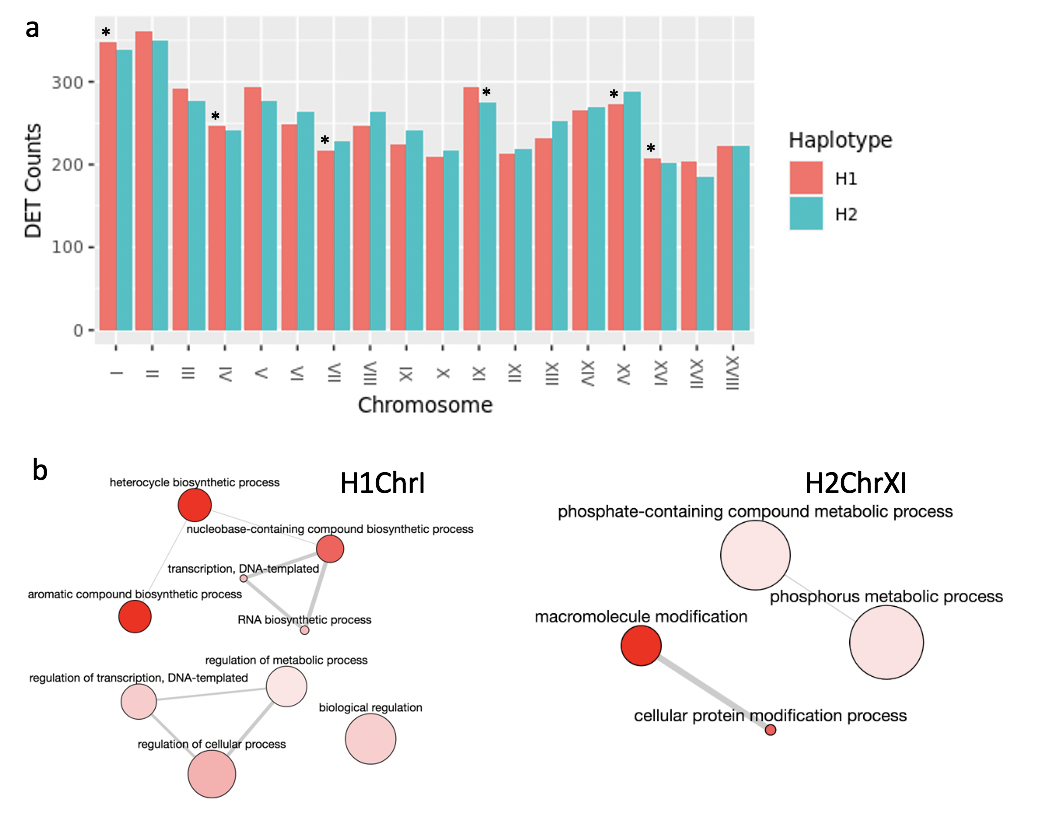


(a) Distribution of DETs between pseudochromosome pairs in the TME204 diploid genome. Chromosomes where DETs were enriched of unique GO BP terms were highlighted with “*”. (b) ReviGo graphs of enriched BP terms unique for DETs from different chromosomes. H1 Chromosome I and H2 Chromosome XI were plotted because their DETs identified most abundantly enriched BP terms. Each GO term is a node. Related GO terms are connected by edges between the nodes. Node color indicates the Fisher exact test p-value. The lighter the color, the lower the p-value. Node size corresponds to the frequency of the GO term in the entire UniProt database.

**Supplementary figure 11. Improved AUGUSTUS *ab initio* gene prediction sensitivity and specificity using TME204 RNA-seq data**.


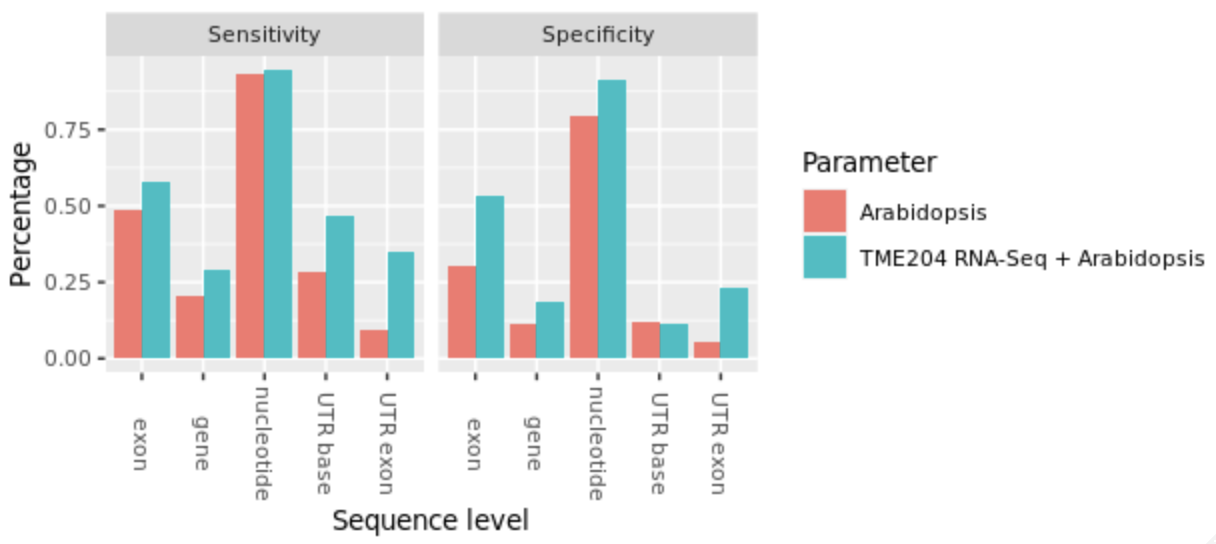


Prediction sensitivity and specificity of exons, genes, coding region nucleotides, UTR nucleotides and UTR exons using AUGUSTUS with a pre-trained Arabidopsis parameter set. This is compared to prediction sensitivity and specificity achieved with a parameter set iteratively trained with cassava TME204 RNA-Seq data, AM560 reference gene models, and the pre-trained Arabidopsis parameter set.

## **Supplementary tables**

## **Supplementary table 1. Assembly statistics for different cassava TME204 primary and alternate genome assemblies based on PacBio CLR and HiFi reads.**

| Contig set | Read type | Assembler | Size (Mbp) | N50 (Mbp) | BUSCO complete (%) | BUSCO duplicate (%) |
| --- | --- | --- | --- | --- | --- | --- |
| Primary | CLR | Falcon (pb-assembly v0.06) | 1176.669 | 1.261 | 95.3 | 46.5 |
|  | HiFi | Falcon (pb-assembly v0.08) | 995.112 | 3.841 | 91.2 | 14.7 |
|  |  | Hifiasm v0.7 | 922.872 | 29.016 | 96.8 | 15.6 |
|  |  | IPA v1.0.5 | 1014.359 | 4.698 | 96.8 | 43.5 |
| Alternate | CLR | Falcon (pb-assembly v0.06) | 58.250 | 0.267 | 18.1 | 0.7 |
|  | HiFi | Falcon (pb-assembly v0.08) | 185.785 | 0.438 | 38.6 | 1.9 |
|  |  | Hifiasm v0.7 | 617.610 | 0.274 | 83.8 | 14.7 |
|  |  | IPA v1.0.5 | 223.050 | 0.647 | 51.6 | 2.1 |

**Supplementary table 2. Assembly statistics for different cassava TME204 combined assemblies of mixed-haplotypes based on PacBio CLR and HiFi reads.**

| Read type | CLR | HiFi | HiFi | HiFi | HiFi |
| --- | --- | --- | --- | --- | --- |
| Assembler | Falcon (pb-assembly v0.06) | Falcon (pb-assembly v0.08) | HiCanu (Canu v2.0) | Hifiasm (v0.7) | IPA (v1.05) |
| Assembled sequences | Primary and associated contigs | Primary and associated contigs | Contigs of all resolved  Alleles | Primary and purged contigs, phased haplotigs | Primary and phased haplotigs |
| Number of bases (Gbp) | 1.235 | 1.181 | 1.484 | 1.540 | 1.237 |
| Contig N50 (Mbp) | 1.189 | 2.803 | 8.131 | 6.464 | 3.363 |
| Contig NG50 (Mbp) | 2.775 | 5.288 | 23.781 | 33.087 | 6.302 |
| Largest contig (Mbp) | 7.773 | 18.554 | 42.800 | 42.956 | 17.899 |
| BUSCO complete (%) | 95.6 | 92.5 | 96.8 | 96.3 | 96.5 |
| BUSCO duplicate (%) | 54.0 | 28.1 | 82.1 | 82.1 | 76.5 |

**Supplementary table 3. Cassava TME204 genome assembly benchmarking results for PacBio CLR and HiFi reads, measured by alignments and k-mer analysis of Illumina PE reads.**

| Read types | CLR | HiFi | | | |
| --- | --- | --- | --- | --- | --- |
| Assemblers | Falcon (pb-assembly v0.06) | Falcon (pb-assembly v0.08) | HiCanu (Canu v2.0) | Hifiasm (v0.7) | IPA (v1.0.5) |
| Assembled sequences | All resolved alleles combined | | | | |
| Mapping rate (%) | 98.29 | 98.21 | 99.88 | 99.88 | 98.21 |
| Properly paired (%) | 94.75 | 95.41 | 99.33 | 99.32 | 95.12 |
| Singletons (%) | 0.34 | 0.41 | 0.02 | 0.02 | 0.42 |
| Mate mapped to a different contig (%) | 3.10 | 2.30 | 0.48 | 0.48 | 2.61 |
| Other wrongly pairs (%) | 0.1 | 0.09 | 0.05 | 0.06 | 0.06 |
| General error rate (%) | 0.68 | 0.59 | 0.21 | 0.21 | 0.38 |
| Total k-mer in the assembly | 1,234,874,650 | 1,180,852,607 | 1,483,627,597 | 1,540,334,405 | 1,237,344,741 |
| K-mer not in Illumina data | 38,973,344 | 39,754,338 | 2,157,169 | 652,749 | 1,690,846 |
| K-mer survival rate (%) | 99.840 | 99.829 | 99.993 | 99.997 | 99.993 |
| Consensus quality (QV) | 27.95 | 27.67 | 41.38 | 46.74 | 41.65 |
| K-mer completeness | 93.20 | 94.96 | 98.40 | 98.40 | 97.54 |

**Supplementary table 4. Cassava TME204 BAC-to-haplotig alignments.**

| BAC | Length (bp) | Haplotype 1 | | | Haplotype 2 | | |
| --- | --- | --- | --- | --- | --- | --- | --- |
|  |  | Haplotig | Number aligned blocks | Number matches (bp) | Haplotig | Number aligned blocks | Number matches (bp) |
| 17I02 | 110289 | h1tg000017l | 2^a^ | 110288 | h2tg000015l | 433 | 76783 |
| 17K17 | 128326 | h1tg000017l | 1 | 128326 | h2tg000015l | 288 | 44218 |
| 50D18 | 95702 | h1tg000017l | 253 | 74458 | h2tg000015l | 2^a^ | 95701 |
| 82K24 | 126840 | h1tg000017l | 1 | 126835^b^ | h2tg000015l | 414 | 88170 |

^a^ The two-block alignment was due to one insertion (1bp) in the assembled BAC sequence.

^b^ A 5 bp sequence at the 3’ end of the assembled BAC sequence was not aligned. Manual inspection revealed that they belong to the cloning vector.

**Supplementary table 5. Hi-C scaffolds in cassava TME204 haplotype 2 assembly.**

| Scaffold ID | Scaffold length (Mbp) | Haplotig ID | Chromosome placement by the genetic map |
| --- | --- | --- | --- |
| 1 | 113 | h2tg000005l | XV |
|  |  | h2tg000034l | XV |
|  |  | h2tg000017l | I |
|  |  | h2tg000006l | XVIII |
|  |  | h2tg000046l | XVIII |
|  |  | h2tg000035l | XVIII |
| 3 | 41 | h2tg000008l | II |
|  |  | h2tg000011l | II |
| 6 | 35 | h2tg000032l | IV |
|  |  | h2tg000010l | IV |
|  |  | h2tg000024l | IV |
| 7 | 34 | h2tg000022l | XVI |
|  |  | h2tg000031l | XVI |
| 8 | 34 | h2tg000019l | XVII |
|  |  | h2tg000047l | XVII |
| 9 | 34 | h2tg000009l | III |
|  |  | h2tg000030l | III |
| 10 | 33 | h2tg000007l | V |
|  |  | h2tg000002l | V |
| 12 | 32 | h2tg000037l | VI |
|  |  | h2tg000033l | VI |
|  |  | h2tg000026l | VI |
| 37 | 0.4 | h2tg000089c | NA |
|  |  | h2tg000642l | NA |
| 102 | 0.1 | h2tg000530l | NA |
|  |  | h2tg000628l | NA |
| 159 | 0.1 | h2tg000514l | NA |
|  |  | h2tg000694l | NA |
| 168 | 0.1 | h2tg000495l | NA |
|  |  | h2tg000607l | NA |

**Supplementary table 6. Assembled sequences with nuclear mitochondrial pseudogene regions (numt’s) in the TME204 genome.**

| Sequences | H1 | H2 |
| --- | --- | --- |
| Chromosome with numt’s | 16 | 13 |
| Unanchored haplotigs with numt’s | 592 | 265 |

**Supplementary table 7. TME204 PacBio Iso-Seq reads and high quality (HQ ^a^) transcripts.**

| Tissue | PacBio barcode name | Polymerase reads | Accession number | HQ transcripts |
| --- | --- | --- | --- | --- |
| Leaf | bc1008_5p--bc1008_5p | 180,025 | ERR5489420 | 23,103 |
| Stem | bc1012_5p--bc1012_5p | 290,953 | ERR5489421 | 38,333 |
| Root | bc1018_5p--bc1018_5p | 301,754 | ERR5489422 | 36,147 |

^a^ Accuracy 99.9% and above

**Supplementary table 8. Reanalysis of a published TME204 Illumina RNA-seq dataset (6).**

| Accession numbers | Tissue type |
| --- | --- |
| SRR3629818, SRR3629835, SRR3629853 | Leaf |
| SRR3629824, SRR3629842, SRR3629859 | Stem |
| SRR3629843 | Storage root |
| SRR3629845 | Fibrous root |
| SRR3629837 | Midvein |
| SRR3629838 | Petiole |
| SRR3629840 | Lateral bud |
| SRR3629850 | SAM (shoot apical meristem) |
| SRR3629851 | RAM (root apical meristem) |

**Supplementary table 9. Primer sequences used to amplify the probes used for BAC screening.**

| BAC | Primer Name | Primer Sequence |
| --- | --- | --- |
| 17K17 | CMD396 | TTGCAGGAGGACACACCATTGG |
|  | CMD397 | ACCTTGGCAGCCAGTGAGG |
| 17I02 | CMD410 | GACCGCTTTCACTGGTACCCAC |
|  | CMD411 | GCAGTAGAACTCCCACGTCCGT |
| 50D18, 82K24 | CMD408 | ATGCTAGACCGCTGTGCATGC |
|  | CMD409 | GTGGAGAACTGGCCTGCTCAG |

**References**

1. Gonthier L, Bellec A, Blassiau C, Prat E, Helmstetter N, Rambaud C, et al. Construction and characterization of two BAC libraries representing a deep-coverage of the genome of chicory (Cichorium intybus L., Asteraceae). BMC Res Notes. 2010 Aug 11;3:225.

2. Li H. Aligning sequence reads, clone sequences and assembly contigs with BWA-MEM. ArXiv13033997 Q-Bio [Internet]. 2013 May 26 [cited 2020 Nov 30]; Available from: http://arxiv.org/abs/1303.3997

3. Li H, Handsaker B, Wysoker A, Fennell T, Ruan J, Homer N, et al. The Sequence Alignment/Map format and SAMtools. Bioinformatics. 2009 Aug 15;25(16):2078–9.

4. Okonechnikov K, Conesa A, García-Alcalde F. Qualimap 2: advanced multi-sample quality control for high-throughput sequencing data. Bioinformatics. 2016 Jan 15;32(2):292–4.

5. Rhie A, Walenz BP, Koren S, Phillippy AM. Merqury: reference-free quality, completeness, and phasing assessment for genome assemblies. Genome Biol. 2020 Sep 14;21(1):245.

6. Wilson MC, Mutka AM, Hummel AW, Berry J, Chauhan RD, Vijayaraghavan A, et al. Gene expression atlas for the food security crop cassava. New Phytol. 2017 Mar;213(4):1632–41.

7. Dobin A, Davis CA, Schlesinger F, Drenkow J, Zaleski C, Jha S, et al. STAR: ultrafast universal RNA-seq aligner. Bioinformatics. 2013 Jan;29(1):15–21.

8. Hoff KJ, Stanke M. Predicting Genes in Single Genomes with AUGUSTUS. Curr Protoc Bioinforma. 2019;65(1):e57.

9. Li H. Minimap2: pairwise alignment for nucleotide sequences. Bioinformatics. 2018 Sep 15;34(18):3094–100.

10. Brůna T, Hoff KJ, Lomsadze A, Stanke M, Borodovsky M. BRAKER2: Automatic Eukaryotic Genome Annotation with GeneMark-EP+ and AUGUSTUS Supported by a Protein Database. bioRxiv. 2020 Aug 11;2020.08.10.245134.

11. Kent WJ. BLAT—The BLAST-Like Alignment Tool. Genome Res. 2002 Apr 1;12(4):656–64.

12. Chin C-S, Peluso P, Sedlazeck FJ, Nattestad M, Concepcion GT, Clum A, et al. Phased diploid genome assembly with single-molecule real-time sequencing. Nat Methods. 2016 Dec;13(12):1050–4.

13. Cheng H, Concepcion GT, Feng X, Zhang H, Li H. Haplotype-resolved de novo assembly with phased assembly graphs. ArXiv200801237 Q-Bio [Internet]. 2020 Aug 3 [cited 2020 Nov 27]; Available from: http://arxiv.org/abs/2008.01237

14. Nurk S, Walenz BP, Rhie A, Vollger MR, Logsdon GA, Grothe R, et al. HiCanu: accurate assembly of segmental duplications, satellites, and allelic variants from high-fidelity long reads. Genome Res. 2020 Aug 14;gr.263566.120.

15. Kuon J-E, Qi W, Schläpfer P, Hirsch-Hoffmann M, von Bieberstein PR, Patrignani A, et al. Haplotype-resolved genomes of geminivirus-resistant and geminivirus-susceptible African cassava cultivars. BMC Biol. 2019 18;17(1):75.

16. Earl D, Bradnam K, St John J, Darling A, Lin D, Fass J, et al. Assemblathon 1: a competitive assessment of de novo short read assembly methods. Genome Res. 2011 Dec;21(12):2224–41.

17. Simpson JT. Exploring genome characteristics and sequence quality without a reference. Bioinformatics. 2014 May 1;30(9):1228–35.

18. Simpson JT, Durbin R. Efficient de novo assembly of large genomes using compressed data structures. Genome Res. 2012 Mar;22(3):549–56.

19. Supek F, Bošnjak M, Škunca N, Šmuc T. REVIGO Summarizes and Visualizes Long Lists of Gene Ontology Terms. PLOS ONE. 2011 Jul 18;6(7):e21800.

20. Conway JR, Lex A, Gehlenborg N. UpSetR: an R package for the visualization of intersecting sets and their properties. Bioinformatics. 2017 Sep 15;33(18):2938–40.

21. Nattestad M, Schatz MC. Assemblytics: a web analytics tool for the detection of variants from an assembly. Bioinformatics. 2016 Oct 1;32(19):3021–3.
