# Supplementary material for: The haplotype-resolved chromosome pairs of a heterozygous diploid African cassava cultivar reveal novel pan-genome and allele-specific transcriptome features
Source: Gigascience. 2022 Mar 24;11:giac028. doi: 10.1093/gigascience/giac028 (PMC8952263; doi:10.1093/gigascience/giac028)
Supplement: giac028_Response_to_Reviewer_Comments_Revision_1 [file giac028_response_to_reviewer_comments_revision_1.pdf]

Author's Response To Reviewer Comments

Close

Answers to Reviewer reports:

Reviewer #1: In this manuscript, Qi et al. assembled two chromosome-scale haploid genomes in African cassava TME204, validated the structural and phasing accuracy of haplotigs by BACs and high-density genetic map, revealed extensive chromosome re-arrangements and abundant intra-genomic and inter-genomic divergent sequences, analyzed the allele-specific expression patterns in different tissues, and built a cassava pan-genome and demonstrated its importance in down-stream omics analysis.

Overall, this work is of crucial importance and should be sufficient to publish in the GigaScience Journal.

Answer: Thanks for the positive feedback.

However, I found that this manuscript lacks the basic logical and some analyses have major flaws.
Please see the details below:
1) According to Supplementary table10, there were at least 9 different tissues of the TME204 Illumina RNA-seq data. However, when the authors performing analysis of 'Tissue specific differentially expressed transcripts (Line 393)', why just compared between leaf and stem but ignore the remaining tissues? This is illogical.

Answer: We have now included analysis results of all datasets for the nine tissue types in the revised manuscript.

2) Two cassava haplotypes (H1 and H2) were constructed in this study. In Table 4 and Supplementary figure 9, why the authors performed analysis between 'TME204 H1 vs. AM560' but did not mention the comparison between 'TME204 H2 vs. AM560' at all? Similarly, in Fig. 8 and Fig. 10c, the analysis was also performed in 'TME204 H1' but not in 'TME204 H2'.

Answer: We added the comparison between AM560 and TME204 H2 in Table 4, Supplementary figure 9, Figure 8 and Figure 10. The results were not presented earlier because the information is very similar to the comparison of AM560 vs. TME204 H1 and therefore we felt it was redundant.

3) in Fig.7C, ASE should be the expression level comparisons between H1 and H2, why the legends still are H1 (red bar) and H2 (blue bar)? I cannot understand.

Answer: In Fig. 7C, H1 and H2 represent two haplotypes of each chromosome. The height of each bar represents the number of transcripts with ASE biased towards a given haplotype (i.e. H1 or H2). We have added the information.

Also in Fig. 7D, it's very difficult to understand this figure. E.g., what's the meaning of labels (e.g., "leaf_H1" and "Stem_H1; Leaf_H1") on x-axis? Logically, there are "stem_H1; leaf_H1", "stem_H1; leaf_H2", "stem_H2; leaf_H2", then where is the "stem_H2; leaf_H1"?

Answer: “Leaf_H1” represents transcripts in leaf tissue with ASE biased towards H1 alleles; "Stem_H1; Leaf_H1" represent transcripts with ASE biased towards H1 alleles in both stem and leaf tissues. After repeated the analysis with all nine tissues, we have changed this ASE figure to upset plots, since the number of transcript sets and possible intersections between/among sets were much higher in the repeated analysis. In the upset plots, not all possible intersections are included for the following two reasons: 1) Empty intersections are not shown; 2) When more tissues are included in the analysis, only the top 40 intersection categories are displayed.

4) Fig6d, Line 110-111, "The transcriptome comparison between TME204 leaf and stem tissues identified

gene loci with associated transcripts that were differentially regulated in one haplotype only." This statement is not true because the comparison between leaf and stem cannot conclude that the transcripts were differentially regulated in one haplotype only. Thus, the sentences in Line 407-408 also need to be revised.

Answer: This is a misunderstanding. The transcriptome analysis was performed using transcripts from both TME204 haplotypes, thus the haplotype origin of each transcript was known. We have revised the analysis and rewritten the results after analyzing the TME204 RNA-seq data from all nine tissues. We grouped differentially expressed transcripts by haplotype origin and performed gene ontology enrichment analysis. We found that transcripts from H1 were enriched for more biological process terms than transcripts from H2. We have rewritten the results section accordingly.

Other suggestions to the authors:
- Fig6a, what's meaning of Het_Uniq, Het_Dup, Hom_Uniq, and Hom_Dup.

Answer: To construct the haplotype-resolved transcriptome, transcript sequences from each haplotype were first labelled either as H1 or H2 in their transcript IDs. Transcript sequences were then clustered based on sequence similarity. Transcript sequences that were identical in both haplotypes were counted as homozygous (Hom) transcripts. Transcript sequences that were different between haplotypes were counted as heterozygous (Het) transcripts. Transcripts present only once within one haplotype were counted as unique (Uniq). Transcripts present multiple times within one haplotype (i.e. due to gene duplication events) were counted as duplicated (Dup). This is explained in the Methods section. In the revised manuscript, we use het_sc, het_mc, hom_sc and hom_mc, respectively, where “sc” representes “single-copy”, “mc” represents “multi-copy”.

- Fig6d, what's the meaning of legend bar? Log2(leaf/stem) or log2(stem/leaf)?

Answer: Fig6d has been replaced by a heatmap to include all nine TME204 tissues. The color scale of the heatmap is explained in the figure legend.

- ref30 cannot be cited because it is still under preparation.

Answer: Removed as suggested. To credit the authors properly, we cited the publication about the phytozome database, where the reference genome and annotation are maintained.

- In 'Conclusions section', the statement "The haplotype-resolved genome allows the first systematic view of the heterozygous diploid genome organization in cassava." is inaccurate, because two haplotypes in heterozygous cassava genome have already been published in Hu et al. (2021, Molecular Plant, 10.1016/j.molp.2021.04.009)

Answer: The reference cited by the reviewer is a “Correspondence” of the cassava cultivar SC205 genome that was assembled from PacBio continuous long reads, which are more error-prone than the HiFi sequencing reads reported in our manuscript. The methods section for this “Correspondence” does not indicate that the authors polished the SC205 haplotype assemblies using Illumina short reads to remove errors. This makes it difficult to assess the quality of the haplotype assemblies. The assembly of the SC205 genome reported in this “Correspondence” is very similar to the haplotype-resolved assemblies of the African cassava genomes 60444 and TME3 reported in 2019 (https://doi.org/10.1186/s12915-019-0697-6) that were not cited by the authors. Together, we feel that our conclusion statement is correct as written. In the revised manuscript, we compared our assembly with SC205 and it is clear that TME204 assembly is more continuous (N50 18 Mbp vs. 1.1 Mbp) and more complete (BUSCO complete 99% vs. 89%). We also phased more gene loci (35K instead of 24K). To be precise, we changed our statement to “The haplotype-resolved genome allows a systematic view of the heterozygous diploid genome organization in cassava with improved accuracy, completeness and haplotype resolution.”

- The title is also suggested to be changed because it is not attractive.

Answer: We are now using a more attractive title.

- The citation of 'Figure 10b' (Line 497) and 'Figure 10c' (Line 502) are wrong.

Answer: This has been corrected. We checked the figure panels and their call-outs in the text again

carefully to make sure the panels are called out sequentially.

Reviewer #2: This manuscript describes the sequencing, assembly, annotation and analysis of a cassava genome. The cassava genome has already been published but this manuscript describes the genome of a heterozygous cultivar rather than the slightly inbred cultivar published previously. The authors performed the assembly using a number of assembler programs and benchmarked each assembly. Not surprisingly, they found that hifiasm worked the best with HiFi reads. The authors then did annotation of the genome and performed a set of analyses including allele specific expression and pan-genome analyses.

The manuscript and its genome will be of use to a range of users in the genomics field. I do feel that the manuscript is exceedingly long and reads more of a dissertation than a research article. A significant portion of the text could be deleted and not impact the take home messages in the manuscript.

Answer: We have re-organized the manuscript and moved technical details to the Supplementary Methods and Results.

For example, the analysis of allele specific expression, alternative splice form expression and the pan-genome is extremely limited in depth and breadth. If these remain in the manuscript, the authors should perform more extended analyses including examining a wider range of tissues and genomes as there are extensive genomic resources available for cassava. It would be nice to tie this complete, phased assembly with the diversity analyses done previously with cassava that revealed the bases of genetic load.

Answer: We agree with the comment and expanded differential expression analysis and analysis of allele-specific expression by including all nine TME 204 tissue types. The pan-genome analysis in the manuscript was aimed at comparing the three haploid genomes together, complementary to other pairwise comparisons. For this purpose, we feel that the depth and breadth are appropriate. We have sequenced and assembled additional cassava genomes for which a more in depth pan-genome analysis is currently on-going and that includes previously reported cassava genome diversity analyses. This in-depth cassava pan-genome analysis will be reported as a separate papers because it goes beyond the scope of our current manuscript.

De novo annotation of the assembly was not performed. Instead, the authors projected the reference annotation onto their assembly and then did alignments with transcript data derived from IsoSeq. The authors are misinterpreting the pseudogenes. As shown earlier by Gan et al. (2011) with Arabidopsis, projection reference annotation on other genome assemblies fails to capture alternative splice forms and thus, predictions of pseudogenes from projected annotation are grossly in accurate. De novo annotation using cognate transcript evidence should be performed to ensure artifacts are not introduced into the annotation. This also would allow the authors to more deeply investigate the dysfunctional/deleterious alleles that are present in casava, a vegetatively propagated crop.

Answer: The tool we used to transfer the AM560 reference gene models was recently developed using a more sophisticated algorithm. However, it is true that the pseudogenes discovered with this method were inaccurate. Therefore, we have now added ab initio annotation using the AUGUSTUS tool with multiple lines of evidence, including RNA-seq and Iso-seq data from the same sample, plus protein and transcript sequences from the reference genome. The transcriptome analysis was revised based on the ab inito predicted transcripts.

Close
