# Supplementary material for: The haplotype-resolved chromosome pairs of a heterozygous diploid African cassava cultivar reveal novel pan-genome and allele-specific transcriptome features
Source: Gigascience. 2022 Mar 24;11:giac028. doi: 10.1093/gigascience/giac028 (PMC8952263; doi:10.1093/gigascience/giac028)
Supplement: giac028_Response_to_Reviewer_Comments_Revision_2 [file giac028_response_to_reviewer_comments_revision_2.pdf]

Author's Response To Reviewer Comments

Close

Answers to reviewer reports:

Reviewer #1: All my concerns have been solved. Thus, I recommend to accept this manuscript.

Answer: Thank you for the positive feedback. The reviewer's comments were helpful for improving the manuscript.

However, before final publication, I suggest the authors to carefully check the typos throughout the whole manuscript. Here I just show you a few examples:

Answer: Thank you for finding small mistakes. Spelling and grammar in all documents have been checked in Microsoft Words again and by a native English speaker.

1) Line 128 and 131, "a fold change (FC) > |4|" is not right. Should be log2|FC| >2? Please check. Similar errors were found in Lines 153-155.

Answer: The reviewer is correct. All have been corrected.

2) Line 217-218, 'p-vale' should be 'p-value'

Answer: Corrected.

3) Fig.6a, in the right sub-figure, DE might mean "differentially expressed", what does EXP mean? expression?

Answer: Yes. "DE" represents "differentially expressed". "EXP" represents "expressed". The explanations have been added into the figure legends of Fig 6a and Fig 6d.

4) Fig.6c, which color (yellow or purple) represents high and low expression level, respectively?

Answer: The color scale of the heatmap has been added into Fig 6c.

Reviewer #2: The revised manuscript is greatly improved; the authors addressed all of my previous concerns.

Answer: Thank you for the positive feedback. The reviewer's comments were helpful for improving the manuscript.

Abstract Line 3-4: The lack of genetic gain in cassava is due to many factors, not solely the lack of the ability to generate a reference genome due to its heterozygosity. Rephrase.

Answer: The sentence "Genetic gain by molecular breeding is limited because cassava has a highly heterozygous, repetitive and difficult to assemble genome." has been rephrased to the following: "Genetic gain by molecular breeding has been limited, partially because cassava is a highly heterozygous crop with a repetitive and difficult to assemble genome."

I think it would be better to use "variable" or "diverged" to refer to ASE rather than inconsistent which implies an experimental problem.

Answer: The terminology of "consistent ASE bias" and "inconsistent ASE bias" have been established and used in multiple publications (Muzzey et al, Crowley et al, Shao et al; see references below). As defined

by Shao et al., “genes showing ASE (ASEGs) in various ways, which can be classified into two patterns: consistent ASEGs such that the ASE was biased toward one parental allele in all tissues/conditions, and inconsistent ASEGs such that ASE was found in some but not all tissues/conditions, including direction-shifting ASEGs in which the ASE was biased toward one parental allele in some tissues/conditions while toward the other parental allele in other tissues/conditions.” We therefore follow the established terminology of previous publications and use “inconsistent” to described ASE biases in our manuscript.

Muzzey D, et al. Extensive and coordinated control of allele-specific expression by both transcription and translation in Candida albicans. Genome Res. 2014 Jun;24(6):963-73. doi: 10.1101/gr.166322.113.

Crowley JJ, et al. Analyses of allele-specific gene expression in highly divergent mouse crosses identifies pervasive allelic imbalance [published correction appears in Nat Genet. 2015 Jun;47(6):690]. Nat Genet. 2015;47(4):353-360. doi:10.1038/ng.3222.

Shao L, et al. Patterns of genome-wide allele-specific expression in hybrid rice and the implications on the genetic basis of heterosis. Proc Natl Acad Sci U S A. 2019 Mar 19;116(12):5653-5658. doi: 10.1073/pnas.1820513116.

Line 80: Explain how being clonally propagated can increase the number of effective alleles. What is an effective allele? F1 hybrids are heterozygous so how is clonal propagation unique?

Answer: In the manuscript we wrote that “Clonally propagation can increase the effective number of alleles and heterozygosity in the population.” We explain this statement below supported by relevant literature references. We are not further elaborating the statement in our current manuscript because this will be further explained and substantiated by results in another manuscript that is currently in preparation.

“Clonal propagation leads to accumulation of somatic mutations [1,2]. As mutations accumulate over time, the growing plant can become a genetic mosaic [3], with cell lineages differing in somatic mutations. A cell lineage with a mutation conferring an advantage in terms of cell proliferation can replace other lineages. Such diplontic selection at the cellular level can purge deleterious mutations, allowing favorable new alleles to accumulate in the plant [4,5]. Gametes can bear the somatic mutations of the cell lineage from which they are derived. When different somatic mutations from different cell lineages are inherited, the clonal descendants of the plant can be genetically heterogeneous. Because continued diplontic selection leads to accumulation of favorable mutations, the gametes are not a random set but are also biased towards favorable mutations. As a source of genetic variation, it has been shown that inherited somatic mutations could be quantitatively comparable to mutations occurring during meiosis [6].”

1. Whitham TG, Slobodchikoff CN. Evolution by individuals, plant-herbivore interactions, and mosaics of genetic variability: The adaptive significance of somatic mutations in plants. Oecologia. 1981; doi: 10.1007/BF00347587.
2. McKey D, Elias M, Pujol B, Duputié A. The evolutionary ecology of clonally propagated domesticated plants. New Phytol. 2010; doi: 10.1111/j.1469-8137.2010.03210.x.
3. Gill DE, Chao L, Perkins SL, Wolf JB. Genetic Mosaicism in Plants and Clonal Animals. Annu Rev Ecol Syst. 1995; doi: 10.1146/annurev.es.26.110195.002231.
4. Otto SP, Hastings IM. Mutation and selection within the individual. Genetica. 102–103:507–241998;
5. Pineda-Krch M, Lehtila K. Cell lineage dynamics in stratified shoot apical meristems. J Theor Biol. 2002; doi: 10.1006/jtbi.2002.3139.
6. Orive ME. Somatic mutations in organisms with complex life histories. Theor Popul Biol. 2001; doi: 10.1006/tpbi.2001.1515.

Clarify which version of hifiasm was used in Supp table 1 vs Table 2. The metrics for the alternate assembly in Supp Table 1 seem poor.

Answer: The version numbers have been added in Supp table 1 and the header of Table 2. The alternate assembly in Supp Table 1 was without Hi-C phasing. The alternate assembly in Table 2 was phased with Hi-C.

Line 298 and others: It should be non-mitochondrial not none-mitochondrial

Answer: Corrected.

If the pseudochromsomes were assigned randomly to H1 and H2, how can you explain differential GO enrichment in the ASE? What would happen to the enrichment if the pseudochromsomes were reshuffled among the haplotypes?

Answer: We have now explained this in the discussion and added the new Supplementary figure 10: “Between allelic chromosome pairs, the number of ASE transcripts biased towards each haplotype was similar across the genome (Figure 7 d). Finding ASE transcripts will thus still hold true after reshuffling of pseudochromosomes between haplotypes. The number of DETs between allelic chromosome pairs was also similar (Supplementary figure 10 a). Functional enrichment analysis of DETs per chromosome identified chromosomes with uniquely enriched BP terms (Supplementary figure 10 a and b). The existence of such chromosomes suggests that haplotype-specific enriched functions in DETs will hold true no matter how the chromosomes are shuffled between haplotypes, although the specific set of DETs and actual enriched GO terms per haploid genome will change.”

Close
