# Supplementary material for: The haplotype-resolved chromosome pairs of a heterozygous diploid African cassava cultivar reveal novel pan-genome and allele-specific transcriptome features
Source: Gigascience. 2022 Mar 24;11:giac028. doi: 10.1093/gigascience/giac028 (PMC8952263; doi:10.1093/gigascience/giac028)
Supplement: giac028_Response_to_Reviewer_Comments_Revision_3 [file giac028_response_to_reviewer_comments_revision_3.pdf]

Author's Response To Reviewer Comments

Close

Dear Hongling,

Many thanks for your careful edits and patience. We have re-uploaded all figures following guidelines in your email request (appended). In details:

1) Figures are provided as separate Microsoft word document files.
2) Each figure fits on a single page in portrait format as a single file.
3) Each multi-panel figure is a single composite file that contains all parts of the figure.
4) Figure titles and legends are provided as a seperated Microsoft word document file. The length of all figure titles and legends have been checked. Each figure title has a max of 15 words. Each legend has a max of 300 words.

Kind regards,

Weihong Qi

On 21.02.22 11:14, Hongling Zhou wrote:

Dear Dr.Qi,

We have made some minor changes (see the attachment), and I have uploaded it for you in the EM system. Could you please upload all figures according to our requirements? See below:

When preparing figures for the journal, please follow the formatting instructions below.

Figures should be provided as separate files, not embedded in the main manuscript file.
Each figure of a manuscript should be submitted as a single file that fits on a single page in portrait format.
Tables should NOT be submitted as figures but should be included in the main manuscript file.
Multi-panel figures (those with parts a, b, c, d etc.) should be submitted as a single composite file that contains all parts of the figure.
Figures should be numbered in the order they are first mentioned in the text, and uploaded in this order.
Figures should be uploaded in the correct orientation.
Figure titles (max 15 words) and legends (max 300 words) should be provided in the main manuscript, not in the graphic file.
Figure keys should be incorporated into the graphic, not into the legend of the figure.
Each figure should be closely cropped to minimize the amount of white space surrounding the illustration. Cropping figures improves accuracy when placing the figure in combination with other elements when the accepted manuscript is prepared for publication on our site. For more information on individual figure file formats, see our detailed instructions.
Individual figure files published in the journal should not exceed 10 MB. If a suitable format is chosen, this file size is adequate for extremely high quality figures. Supplemental files and original source images larger than 10MB should be hosted in our GigaScience GigaDB repository so please contact our curators (database@gigasciencejournal.com) if you have any of these. For image data, GigaDB additionally requires details of bit-depth, pixel resolution, and voxel resolution. Including this information in the manuscript is also recommended. More details can be found at the following link: http://gigadb.org/site/guideimaging.
If you have 3D models, we can additionally support these in our interactive GigaDB 3D viewer. 3D reconstructions should be submitted in STL or OBJ format and are made publicly available in Sketchfab (https://sketchfab.com/GigaDB) and to the 3D printing community via Thingiverse (https://www.thingiverse.com/GigaScience/designs).
As an Open Access journal all figures, images and other media are published under the Creative Commons Attribution (CC BY 4.0) license.

NOTE that figures purchased from commercial photo libraries do NOT allow you to transfer rights to a journal for unlimited publication - and these types of photos CANNOT be used in the journal or any other material produced by the journal.
Please note that it is the responsibility of the author(s) to obtain permission from the copyright holder to reproduce figures (or tables) that have previously been published elsewhere. In order for all figures to be published under our Open Access license, authors must have permission from the rights holder if they wish to include images that have been published elsewhere in non-open access journals or photo libraries. Permission should be indicated in the figure legend, and the original source included in the reference list. If you do not have owner permission, GigaScience reserves the right to remove that content and/or replace it with other content with permission to use.
If you have any questions or problems please contact the GigaScience Editors and Curators.

I'll send the paper back to you in the EM system, you only need to re-upload the figures. Thank you!

Best wishes,
Hongling

Close
