# Supplementary material for: The haplotype-resolved chromosome pairs of a heterozygous diploid African cassava cultivar reveal novel pan-genome and allele-specific transcriptome features
Source: Gigascience. 2022 Mar 24;11:giac028. doi: 10.1093/gigascience/giac028 (PMC8952263; doi:10.1093/gigascience/giac028)
Supplement: giac028_Reviewer_1_Report_Original_Submission — Zehong Ding -- 11/11/2021 Reviewed [file giac028_reviewer_1_report_original_submission.pdf]

Reviewer Report

Title: The haplotype-resolved chromosome pairs of a heterozygous diploid African cassava cultivar reveal novel pan-genome and allele-specific transcriptome features

Version: Original Submission Date: 11/11/2021

Reviewer name: Zehong Ding

Reviewer Comments to Author:

In this manuscript, Qi et al. assembled two chromosome-scale haploid genomes in African cassava TME204, validated the structural and phasing accuracy of haplotigs by BACs and high-density genetic map, revealed extensive chromosome re-arrangements and abundant intra-genomic and inter-genomic divergent sequences, analyzed the allele-specific expression patterns in different tissues, and built a cassava pan-genome and demonstrated its importance in down-stream omics analysis.
Overall, this work is of crucial importance and should be sufficient to publish in the GigaScience Journal. However, I found that this manuscript lacks the basic logical and some analyses have major flaws. Please see the details below:
1) According to Supplementary table10, there were at least 9 different tissues of the TME204 Illumina RNA-seq data. However, when the authors performing analysis of 'Tissue specific differentially expressed transcripts (Line 393)', why just compared between leaf and stem but ignore the remaining tissues? This is illogical.
2) Two cassava haplotypes (H1 and H2) were constructed in this study. In Table 4 and Supplementary figure 9, why the authors performed analysis between 'TME204 H1 vs. AM560' but did not mention the comparison between 'TME204 H2 vs. AM560' at all? Similarly, in Fig. 8 and Fig. 10c, the analysis was also performed in 'TME204 H1' but not in 'TME204 H2'.
3) in Fig.7C, ASE should be the expression level comparisons between H1 and H2, why the legends still are H1 (red bar) and H2 (blue bar)? I cannot understand. Also in Fig. 7D, it's very difficult to understand this figure. E.g., what's the meaning of labels (e.g., "leaf_H1" and "Stem_H1; Leaf_H1") on x-axis? Logically, there are "stem_H1; leaf_H1", "stem_H1; leaf_H2", "stem_H2; leaf_H2", then where is the "stem_H2; leaf_H1"?
4) Fig6d, Line 110-111, "The transcriptome comparison between TME204 leaf and stem tissues identified gene loci with associated transcripts that were differentially regulated in one haplotype only." This statement is not true because the comparison between leaf and stem cannot conclude that the transcripts were differentially regulated in one haplotype only. Thus, the sentences in Line 407-408 also need to be revised.
Other suggestions to the authors:
- Fig6a, what's meaning of Het_Uniq, Het_Dup, Hom_Uniq, and Hom_Dup.
- Fig6d, what's the meaning of legend bar? Log2(leaf/stem) or log2(stem/leaf)?
- ref30 cannot be cited because it is still under preparation.
- In 'Conclusions section', the statement "The haplotype-resolved genome allows the first systematic view of the heterozygous diploid genome organization in cassava." is inaccurate, because two

haplotypes in heterozygous cassava genome have already been published in Hu et al. (2021, Molecular Plant, 10.1016/j.molp.2021.04.009)
- The title is also suggested to be changed because it is not attractive.
- The citation of 'Figure 10b' (Line 497) and 'Figure 10c' (Line 502) are wrong.

Methods

Are the methods appropriate to the aims of the study, are they well described, and are necessary controls included? Choose an item.

Conclusions

Are the conclusions adequately supported by the data shown? Choose an item.

Reporting Standards

Does the manuscript adhere to the journal's guidelines on minimum standards of reporting? Choose an item.

Choose an item.

Statistics

Are you able to assess all statistics in the manuscript, including the appropriateness of statistical tests used? Choose an item.

Quality of Written English

Please indicate the quality of language in the manuscript: Choose an item.

Declaration of Competing Interests

Please complete a declaration of competing interests, considering the following questions:

- Have you in the past five years received reimbursements, fees, funding, or salary from an organisation that may in any way gain or lose financially from the publication of this manuscript, either now or in the future?
- Do you hold any stocks or shares in an organisation that may in any way gain or lose financially from the publication of this manuscript, either now or in the future?
- Do you hold or are you currently applying for any patents relating to the content of the manuscript?
- Have you received reimbursements, fees, funding, or salary from an organization that holds or has applied for patents relating to the content of the manuscript?
- Do you have any other financial competing interests?

- Do you have any non-financial competing interests in relation to this paper?

If you can answer no to all of the above, write 'I declare that I have no competing interests' below. If your reply is yes to any, please give details below.

I declare that I have no competing interests

I agree to the open peer review policy of the journal. I understand that my name will be included on my report to the authors and, if the manuscript is accepted for publication, my named report including any attachments I upload will be posted on the website along with the authors' responses. I agree for my report to be made available under an Open Access Creative Commons CC-BY license (http://creativecommons.org/licenses/by/4.0/). I understand that any comments which I do not wish to be included in my named report can be included as confidential comments to the editors, which will not be published.

Choose an item.

To further support our reviewers, we have joined with Publons, where you can gain additional credit to further highlight your hard work (see: https://publons.com/journal/530/gigascience). On publication of this paper, your review will be automatically added to Publons, you can then choose whether or not to claim your Publons credit. I understand this statement.

Yes Choose an item.
