# Supplementary material for: The haplotype-resolved chromosome pairs of a heterozygous diploid African cassava cultivar reveal novel pan-genome and allele-specific transcriptome features
Source: Gigascience. 2022 Mar 24;11:giac028. doi: 10.1093/gigascience/giac028 (PMC8952263; doi:10.1093/gigascience/giac028)
Supplement: giac028_Reviewer_2_Report_Original_Submission — C Robin Buell -- 11/17/2021 Reviewed [file giac028_reviewer_2_report_original_submission.pdf]

Reviewer Report

Title: The haplotype-resolved chromosome pairs of a heterozygous diploid African cassava cultivar reveal novel pan-genome and allele-specific transcriptome features

Version: Original Submission **Date:** 11/17/2021

Reviewer name: C Robin Buell

Reviewer Comments to Author:

This manuscript describes the sequencing, assembly, annotation and analysis of a cassava genome. The cassava genome has already been published but this manuscript describes the genome of a heterozygous cultivar rather than the slightly inbred cultivar published previously. The authors performed the assembly using a number of assembler programs and benchmarked each assembly. Not surprisingly, they found that hifiasm worked the best with HiFi reads. The authors then did annotation of the genome and performed a set of analyses including allele specific expression and pan-genome analyses.

The manuscript and its genome will be of use to a range of users in the genomics field. I do feel that the manuscript is exceedingly long and reads more of a dissertation than a research article. A significant portion of the text could be deleted and not impact the take home messages in the manuscript. For example, the analysis of allele specific expression, alternative splice form expression and the pan-genome is extremely limited in depth and breadth. If these remain in the manuscript, the authors should perform more extended analyses including examining a wider range of tissues and genomes as there are extensive genomic resources available for cassava. It would be nice to tie this complete, phased assembly with the diversity analyses done previously with cassava that revealed the bases of genetic load.

De novo annotation of the assembly was not performed. Instead, the authors projected the reference annotation onto their assembly and then did alignments with transcript data derived from IsoSeq. The authors are misinterpreting the pseudogenes. As shown earlier by Gan et al. (2011) with Arabidopsis, projection reference annotation on other genome assemblies fails to capture alternative splice forms and thus, predictions of pseudogenes from projected annotation are grossly in accurate. De novo annotation using cognate transcript evidence should be performed to ensure artifacts are not introduced into the annotation. This also would allow the authors to more deeply investigate the dysfunctional/deleterious alleles that are present in casava, a vegetatively propagated crop.

Methods

Are the methods appropriate to the aims of the study, are they well described, and are necessary controls included? Choose an item.

Conclusions

Are the conclusions adequately supported by the data shown? Choose an item.

Reporting Standards

Does the manuscript adhere to the journal's guidelines on [minimum standards of reporting?]() Choose an item.

Choose an item.

Statistics

Are you able to assess all statistics in the manuscript, including the appropriateness of statistical tests used? Choose an item.

Quality of Written English

Please indicate the quality of language in the manuscript: Choose an item.

Declaration of Competing Interests

Please complete a declaration of competing interests, considering the following questions:

- Have you in the past five years received reimbursements, fees, funding, or salary from an organisation that may in any way gain or lose financially from the publication of this manuscript, either now or in the future?
- Do you hold any stocks or shares in an organisation that may in any way gain or lose financially from the publication of this manuscript, either now or in the future?
- Do you hold or are you currently applying for any patents relating to the content of the manuscript?
- Have you received reimbursements, fees, funding, or salary from an organization that holds or has applied for patents relating to the content of the manuscript?
- Do you have any other financial competing interests?
- Do you have any non-financial competing interests in relation to this paper?

If you can answer no to all of the above, write 'I declare that I have no competing interests' below. If your reply is yes to any, please give details below.

I declare that I have no competing interests below

I agree to the open peer review policy of the journal. I understand that my name will be included on my report to the authors and, if the manuscript is accepted for publication, my named report including any attachments I upload will be posted on the website along with the authors' responses. I agree for my report to be made available under an Open Access Creative Commons CC-BY license (http://creativecommons.org/licenses/by/4.0/). I understand that any comments which I do not wish to be included in my named report can be included as confidential comments to the editors, which will not be published.

Choose an item.

To further support our reviewers, we have joined with Publons, where you can gain additional credit to further highlight your hard work (see: https://publons.com/journal/530/gigascience). On publication of this paper, your review will be automatically added to Publons, you can then choose whether or not to claim your Publons credit. I understand this statement.

Yes Choose an item.
