# Supplementary material for: The haplotype-resolved chromosome pairs of a heterozygous diploid African cassava cultivar reveal novel pan-genome and allele-specific transcriptome features
Source: Gigascience. 2022 Mar 24;11:giac028. doi: 10.1093/gigascience/giac028 (PMC8952263; doi:10.1093/gigascience/giac028)
Supplement: giac028_Reviewer_2_Report_Revision_1 — C Robin Buell -- 1/16/2022 Reviewed [file giac028_reviewer_2_report_revision_1.pdf]

Reviewer Report

Title: The haplotype-resolved chromosome pairs of a heterozygous diploid African cassava cultivar reveal novel pan-genome and allele-specific transcriptome features

Version: Revision 1 **Date:** 1/16/2022

Reviewer name: C Robin Buell

Reviewer Comments to Author:

The revised manuscript is greatly improved; the authors addressed all of my previous concerns.
Abstract Line 3-4: The lack of genetic gain in cassava is due to many factors, not solely the lack of the ability to generate a reference genome due to its heterozygosity. Rephrase.
I think it would be better to use "variable" or "diverged" to refer to ASE rather than inconsistent which implies an experimental problem.
Line 80: Explain how being clonally propagated can increase the number of effective alleles. What is an effective allele? F1 hybrids are heterozygous so how is clonal propagation unique?
Clarify which version of hifiasm was used in Supp table 1 vs Table 2. The metrics for the alternate assembly in Supp Table 1 seem poor.
Line 298 and others: It should be non-mitochrondrial not none-mitochondrial
If the pseudochromsomes were assigned randomly to H1 and H2, how can you explain differential GO enrichment in the ASE? What would happen to the enrichment if the pseudochromsomes were reshuffled among the haplotypes?

Methods

Are the methods appropriate to the aims of the study, are they well described, and are necessary controls included? Choose an item.

Conclusions

Are the conclusions adequately supported by the data shown? Choose an item.

Reporting Standards

Does the manuscript adhere to the journal's guidelines on minimum standards of reporting? Choose an item.

Choose an item.

Statistics

Are you able to assess all statistics in the manuscript, including the appropriateness of statistical tests used? Choose an item.

Quality of Written English

Please indicate the quality of language in the manuscript: Choose an item.

Declaration of Competing Interests

Please complete a declaration of competing interests, considering the following questions:

- Have you in the past five years received reimbursements, fees, funding, or salary from an organisation that may in any way gain or lose financially from the publication of this manuscript, either now or in the future?
- Do you hold any stocks or shares in an organisation that may in any way gain or lose financially from the publication of this manuscript, either now or in the future?
- Do you hold or are you currently applying for any patents relating to the content of the manuscript?
- Have you received reimbursements, fees, funding, or salary from an organization that holds or has applied for patents relating to the content of the manuscript?
- Do you have any other financial competing interests?
- Do you have any non-financial competing interests in relation to this paper?

If you can answer no to all of the above, write 'I declare that I have no competing interests' below. If your reply is yes to any, please give details below.

I declare that I have no competing interests

I agree to the open peer review policy of the journal. I understand that my name will be included on my report to the authors and, if the manuscript is accepted for publication, my named report including any attachments I upload will be posted on the website along with the authors' responses. I agree for my report to be made available under an Open Access Creative Commons CC-BY license (http://creativecommons.org/licenses/by/4.0/). I understand that any comments which I do not wish to be included in my named report can be included as confidential comments to the editors, which will not be published.

Choose an item.

To further support our reviewers, we have joined with Publons, where you can gain additional credit to further highlight your hard work (see: https://publons.com/journal/530/gigascience). On publication of this paper, your review will be automatically added to Publons, you can then choose whether or not to claim your Publons credit. I understand this statement.

Yes Choose an item.
